# Treatment options in idiopathic subglottic stenosis: protocol for a prospective international multicentre pragmatic trial

**DOI:** 10.1136/bmjopen-2018-022243

**Published:** 2018-04-10

**Authors:** Alexander Gelbard, Yu Shyr, Lynne Berry, Alexander T Hillel, Dale C Ekbom, Eric S Edell, Jan L Kasperbauer, David G Lott, Donald T Donovan, C. Gaelyn Garrett, Guri Sandhu, James J Daniero, James L Netterville, Josh S Schindler, Marshall E Smith, Paul C Bryson, Robert R Lorenz, David O Francis

**Affiliations:** 1 Department of Otolaryngology, Vanderbilt University, Nashville, Tennessee, USA; 2 Department of Biostatistics, Vanderbilt University, Nashville, Tennessee, USA; 3 Department of Otolaryngology, Johns Hopkins University, Baltimore, Maryland, USA; 4 Department of Otolaryngology, Mayo Clinic, Rochester, Minnesota, USA; 5 Department of Pulmonology, Mayo Clinic, Rochester, Minnesota, USA; 6 Department of Otorhinolaryngology, Mayo Clinic Scottsdale, Scottsdale, Arizona, USA; 7 Department Otolaryngology, Baylor College of Medicine, Houston, Texas, USA; 8 Department of Otolaryngology, Imperial College Healthcare NHS, London, UK; 9 Department Otolaryngology, University of Virginia Health System, Charlottesville, Virginia, USA; 10 Department of Otolaryngology, Oregon Health and Science University, Portland, Oregon, USA; 11 Department of Otolaryngology, University of Utah, Salt Lake City, Utah, USA; 12 Department of Otolaryngology, The Cleveland Clinic, Cleveland, Ohio, USA; 13 Department of Surgery, University of Wisconsin School of Medicine and Public Health, Madison, Wisconsin, USA

**Keywords:** pragmatic trial, PR02, NoAAC, iSGS, subglottic stenosis

## Abstract

**Introduction:**

Idiopathic subglottic stenosis (iSGS) is an unexplained progressive obstruction of the upper airway that occurs almost exclusively in adult, Caucasian women. The disease is characterised by mucosal inflammation and localised fibrosis resulting in life-threatening blockage of the upper airway. Because of high recurrence rates, patients with iSGS will frequently require multiple procedures following their initial diagnosis. Both the disease and its therapies profoundly affect patients’ ability to breathe, communicate and swallow. A variety of treatments have been advanced to manage this condition. However, comparative data on effectiveness and side effects of the unique approaches have never been systematically evaluated. This study will create an international, multi-institutional prospective cohort of patients with iSGS. It will compare three surgical approaches to determine how well the most commonly used treatments in iSGS ‘work’ and what quality of life (QOL) trade-offs are associated with each approach.

**Methods and analysis:**

A prospective pragmatic trial comparing the ‘Standard of Care’ for iSGS at multiple international institutions. Patients with a diagnosis of iSGS without clinical or laboratory evidence of vasculitis or a history of endotracheal intubation 2 years prior to symptom onset will be included in the study. Prospective evaluation of disease recurrence requiring operative intervention, validated patient-reported outcome (PRO) measures as well as patient-generated health data (mobile peak flow recordings and daily steps taken) will be longitudinally tracked for 36 months. The primary endpoint is treatment effectiveness defined as time to recurrent operative procedure. Secondary endpoints relate to treatment side effects and include PRO measures in voice, swallowing, breathing and global QOL as well as patient-generated health data.

**Ethics and dissemination:**

This protocol was approved by the local IRB Committee of the Vanderbilt University Medical Center in July 2015. The findings of the trial will be disseminated through peer-reviewed journals, national and international conference presentations and directly to patient with iSGS via social media-based support groups.

**Trial registration number:**

NCT02481817.

**Strengths and limitations of this study**

- Unique international prospective cohort of the rare disease idiopathic subglottic stenosis (iSGS).
- Methodology evaluates three common surgical approaches in iSGS using objective clinical endpoints to enable assessment of their ‘real-world’ effectiveness.
- Study design also integrates longitudinal assessment of validated patient-reported outcome measures to compare patient-perceived functional outcomes of three common surgeries in iSGS.
- Non-randomised design.
- Adequate statistical power relies on sufficient recruitment which can be a challenge in rare disease.

## Introduction

### Background and rationale

**Figure 1:**
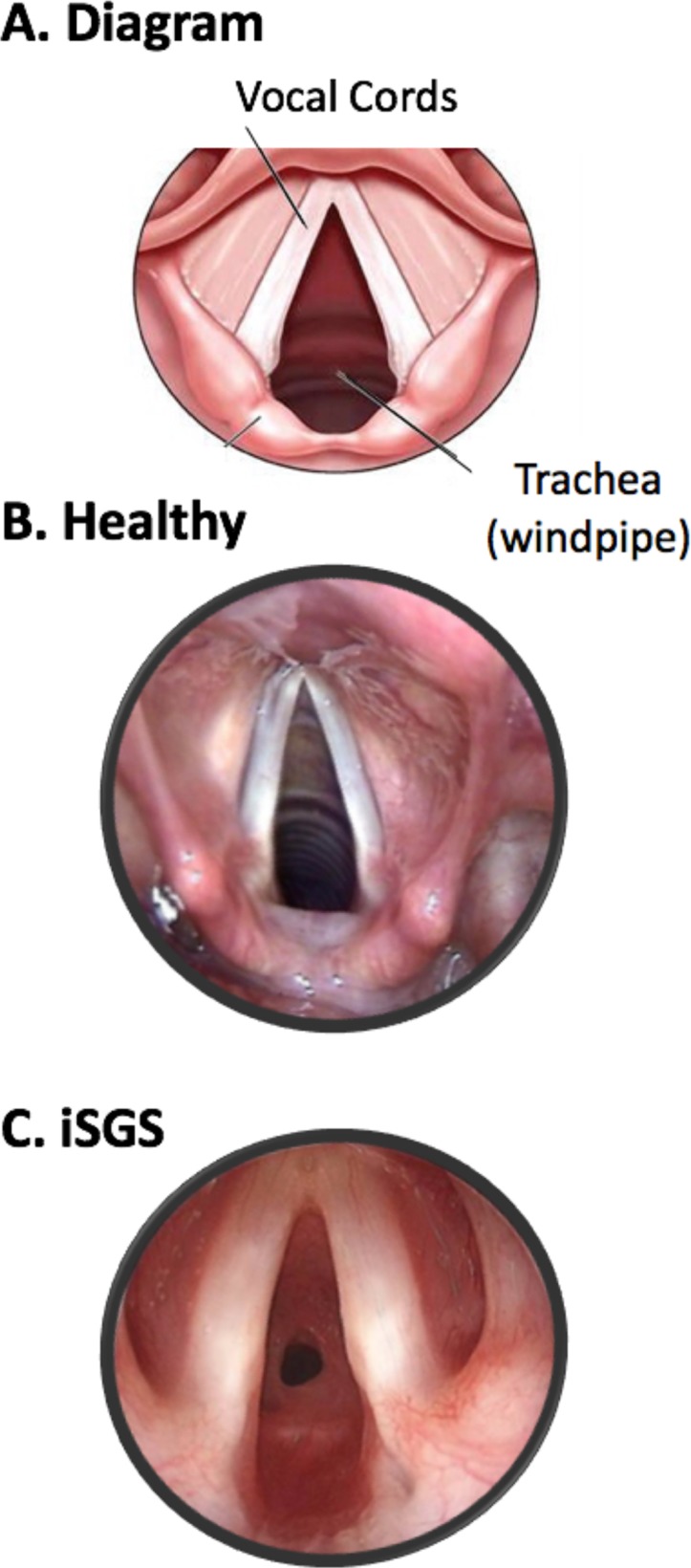
Diagram of normal trachea (A). Healthy trachea (B). Patient with iSGS with obstructive tracheal scar (C). iSGS, idiopathic subglottic stenosis.

Idiopathic subglottic stenosis (iSGS) is an unexplained progressive obstruction of the upper airway that occurs almost exclusively in adult, Caucasian women.^1 2^ The disease is characterised by mucosal inflammation and localised fibrosis resulting in life-threatening blockage of the upper airway. Although uncommon (with an estimated incidence of 1:400 000 persons per year^3^), both the disease and its therapies profoundly affect patients’ ability to breathe,^4^ communicate^5^ and swallow.^6^ Dyspnoea is the hallmark symptom and the primary cause of death and disability.^7^ However, patients can also experience debilitating voice changes^5 8 9^ and swallowing problems^10^ due to the condition (figure 1A–C) or its treatment.

**Figure 2:**
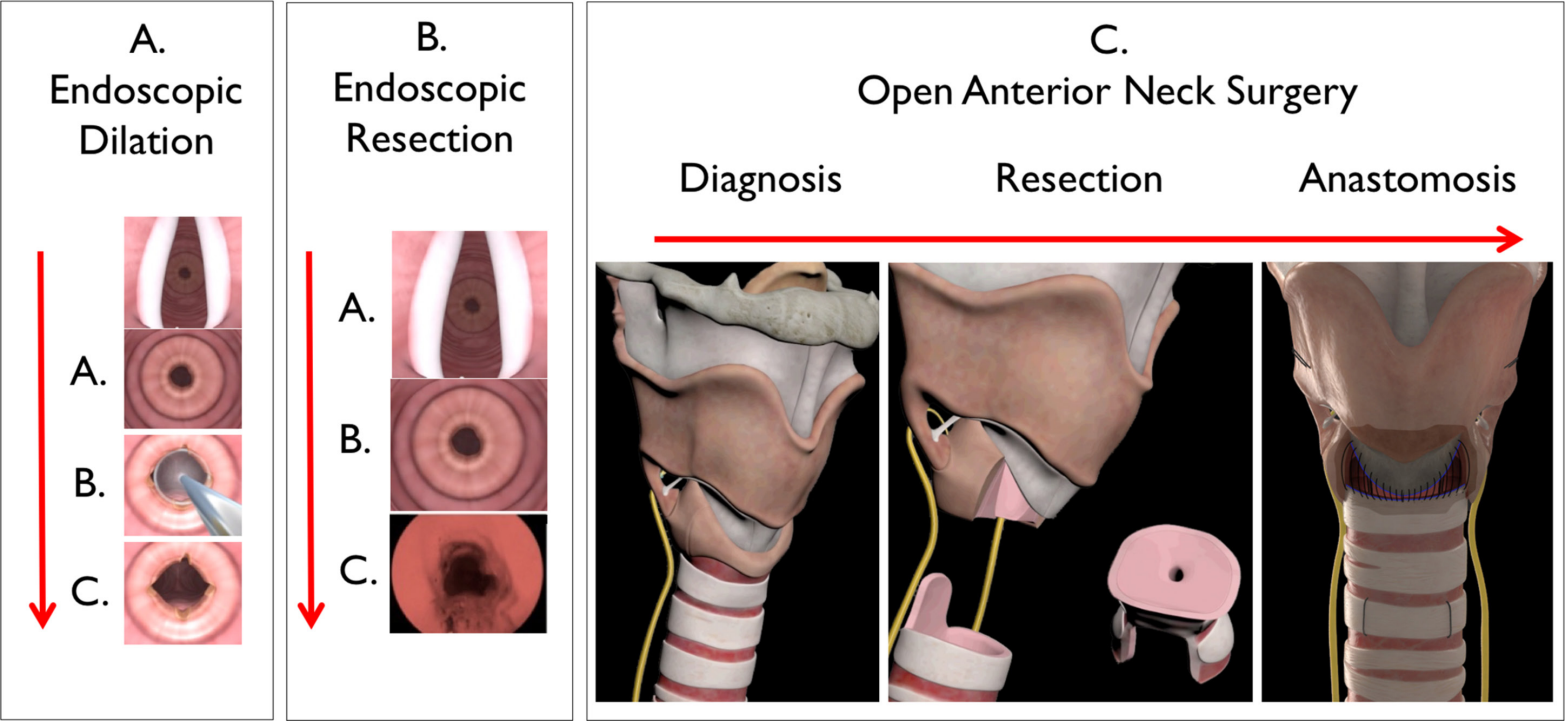
Treatment approaches for iSGS: (A) endoscopic dilation of the tracheal stenosis (accomplished with rigid instruments or inflatable balloons; (B) endoscopic resection of the stenosis (with prolonged medical therapy after surgery; (C) open surgery with resection of the affected tracheal segment with end-to-end anastomosis. iSGS, idiopathic subglottic stenosis.

**Figure 3:**
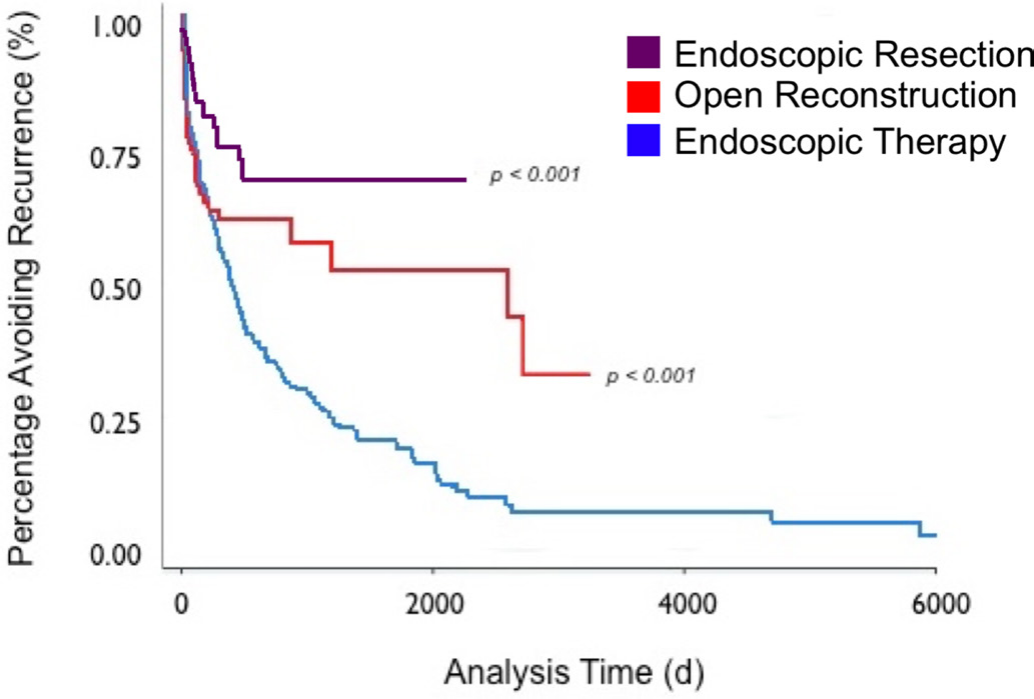
Kaplan-Meier analysis of NoAAC RP01 study results. Percentage of patients with iSGS avoiding disease recurrence, stratified by treatment type. iSGS, idiopathic subglottic stenosis; NoAAC RP01, North American Airway Collaborative RP-01.

People with this disease often require several surgeries per year.^11^ A variety of treatments have been advanced to manage this condition^3 4 11^ but are generally categorised into: (1) endoscopic dilation of the tracheal stenosis (accomplished with rigid instruments or inflatable balloons: figure 2A); (2) endoscopic resection of the stenosis (with prolonged medical therapy after surgery: figure 2B) or (3) open surgery with resection of the affected tracheal segment with end-to-end anastomosis (figure 2C). Each patient can require repeated surgeries to keep their trachea open, which increases odds of treatment side effects and complications. All approaches have unique associated side effects, which can significantly affect the patient’s quality of life (QOL). However, comparative data on trade-offs have never been systematically evaluated.

As a product of disease rarity, there is a lack of high-quality data to inform individual patient decision-making in iSGS. Limited evidence on treatment outcomes complicates patient decision-making as they try to balance survival, symptoms and QOL considerations. The proposed prospective study is designed to fill this void and leverages and expands on a previous retrospective multi-institutional investigation, the North American Airway Collaborative RP-01 (NoAAC RP-01).^1^ In that study, nearly 500 patients with iSGS from 10 expert centres were retrospectively examined for the need for repeat surgery and the time to recurrent disease (figure 3). Interestingly, the demographics of this condition were quite similar at the separate centres. iSGS appears to nearly universally affect otherwise healthy, 40–60-year-old Caucasian women (median age 50.3, 95% CI 49.1 to 51.5)^11^ The NoAAC RP-01 study also found variation in the standard of care for iSGS at expert centres. Three basic treatment approaches were used (ie, endoscopic dilation, endoscopic resection with adjuvant medical therapy and open surgery) despite an absence of randomised controlled trials or other rigorous comparative studies to assess their differential effectiveness at avoiding disease recurrence. In contrast to the retrospective nature of RP-01, the prospective design of the NoAAC PR-02 study will enable rigorous treatment comparisons to determine how well the most commonly used surgeries in iSGS ‘work’.

While evaluating treatment effectiveness is important, it is equally critical that the patient experience with the disease itself and its treatment be systematically characterised. This is imperative since patient and physician perspectives are often significantly discordant.^12^ To this end, NoAAC PR-02 will collect patient-reported outcomes (PROs) in the cohort at initial presentation and at a priori determined intervals thereafter (eg, immediately preintervention as well as 3, 6 and 12 months postintervention) and conduct qualitative patient interviews (labelled ‘engagement studios’) to better understand the health-related QOL (HRQOL) and functional outcomes of therapy in iSGS. These endpoints are important to patients and are arguably a primary determinant in decision-making. For example, results show that endoscopic dilation is associated with a higher rate of disease recurrence and thus need for repeated surgery. Meanwhile, open cricotracheal resection is a major surgery with significant immediate perioperative risks and has been associated with alterations in voice^5 8^ and swallowing.^6^ Open surgery appears to reduce the risk of disease recurrence, but the degree of benefit and the trade-offs associated with this invasive surgery are questions that demand prospective study.

### Explanation for choice of comparators

The most common contemporary treatments for iSGS will be compared. These treatments (described previously) were identified during the preliminary RP-01 study. Patients with this condition have scarring with their trachea and present to the healthcare system with symptoms of airway obstruction (stridor) and respiratory distress. They universally require expeditious intervention to survive. Thus, use of a placebo or ‘no intervention’ control is clinically unethical and inappropriate. The most widely employed treatment is endoscopic dilation of the tracheal scar (60% of patients in RP-01). Patients treated using this approach will be the primary comparator ‘control’ group for this study. Outcomes will be compared with two other interventions: (1) endoscopic resection with adjuvant medical therapy and (2) tracheal resection which comprise approximately 20% of iSGS treatments, respectively.

### Objectives

Specific hypotheses under investigation include:

1. There is variation in time to recurrence (TTR) between centres using similar surgical approaches for iSGS.
2. There is variation in TTR between centres using different surgical approaches for iSGS.
3. The different surgical approaches are associated with unique trade-offs in terms of voice, swallowing, breathing and global QOL.

### Trial design

Prospective Pragmatic Trial investigating treatment effectiveness in the care of patients with iSGS.

## Methods: participants, interventions and outcomes

### Study setting

International academic medical centres. NoAAC Participating Sites can be found at https://noaac.net/about/noaac-sites/. All are academic centres that are tertiary referral centres for iSGS and thus have significant experience treating this rare condition.^3 11 13–16^ Nearly all patients with iSGS are ultimately referred for care and cluster at such high-volume centres and thus we anticipate enrolling a representative patient cohort. Each research site has appropriate technological infrastructure for data collection and pathological specimen tracking. The trial will be registered at ClinicalTrials.gov using the WHO Trial Registration Data Set V.1.3 (online supplementary appendix 1).

10.1136/bmjopen-2018-022243.supp1 Supplementary data

### Eligibility criteria

#### Inclusion criteria

- >18 years of age.
- The obstructive airway lesion must involve the subglottis.

#### Exclusion criteria

- Patients without capacity to consent for themselves.
- History of endotracheal intubation or tracheotomy within 2 years of first symptoms.
- History of significant laryngotracheal traumatic injury.
- History of major anterior neck surgery.
- History of neck irradiation.
- History of caustic or thermal injury to the laryngotracheal complex.
- History of a clinically diagnosed vasculitis or collagen vascular disease.
- Positive antinuclear cytoplasmic antibody titres.

### Interventions

Once consented (online supplementary appendix 2) and enrolled, patients will receive standard of care treatment at the respective centre (online supplementary appendix 3). Employing our established digital trial infrastructure patients will be followed longitudinally for symptom changes, need for further treatment, complications and will have PROs administered at a priori determined intervals.

10.1136/bmjopen-2018-022243.supp2 Supplementary data

10.1136/bmjopen-2018-022243.supp3 Supplementary data

Three interventions being compared include: (1) endoscopic dilation, (2) endoscopic resection of the airway narrowing with subsequent long-term medical therapy and (3) open neck surgery (figure 2). In endoscopic dilation, the patient undergoes transoral exposure of the tracheal scar with dilation of the scar by either rigid instrument or controlled radial expansion device (ie, balloon dilation). Somewhat similarly, in endoscopic resection the patient undergoes transoral exposure of the tracheal scar; however, a CO_2_ laser is then used to resect a significant portion of the scar, followed by long-term adjuvant medical therapy (antireflux, antibacterial and inhaled corticosteroid). In open neck surgery (aka tracheal or cricotracheal resection), the trachea is approached via an external incision, the scarred segment of trachea is resected and the remaining ends sewn back together.

There is significant homogeneity in treatment approach at each participating study. This has been confirmed both through our preliminary RP-01 data and direct correspondence with treating surgeons at these centres. In fact, study sites use a singular surgical approach to treat 95% of their patients with iSGS.^3^ Personal communications with treating surgeons further indicate that the rationale for consistency in approach is largely experiential. To monitor and ensure consistency, each study site principal co-investigator will submit a formalised therapeutic protocol delineating their institutional ‘standard of care’ for patients with iSGS to ensure treatment and data homogeneity (online supplementary appendix 3). Centres are expected adhere to their internal ‘standard of care’ in the management of the patients with iSGS. After surgical treatment, national key study personel (KSP) will review the operative report for deviation from the formalised therapeutic protocol (describing how and why the procedure differed from local standard) and enter the results into the digital trial interface.

### Outcomes

Interviews with both participating providers and patients with iSGS were performed to reach consensus on the primary clinical outcomes to be compared across treatment modalities. Both groups were urged to select outcomes based on what data would be most critical and useful to patients and providers in decision-making. Clinical treatment outcomes identified were: (1) TTR or need for repeat procedure to open up their airway obstruction. The specific primary outcome to be measured is time to second treatment (second–first treatment date). This is an appropriate surrogate measure for recurrence since patient-reported symptoms of respiratory distress are the universal trigger for worsening symptoms, which is nearly perfectly correlated with additional treatment at these study sites. Measuring this outcome is feasible within the 3-year timeframe of the proposed. In our preliminary RP-01 study, recurrence occurred at a median 8 months (95% CI 7.2 to 11.3 months) following initial treatment. It was experienced by 80% of patients. The TTR varied both between patients with iSGS receiving the same treatment and also between the three treatments being prospectively compared in the proposed study (figure 3).

### Participant timeline

**Figure 4:**
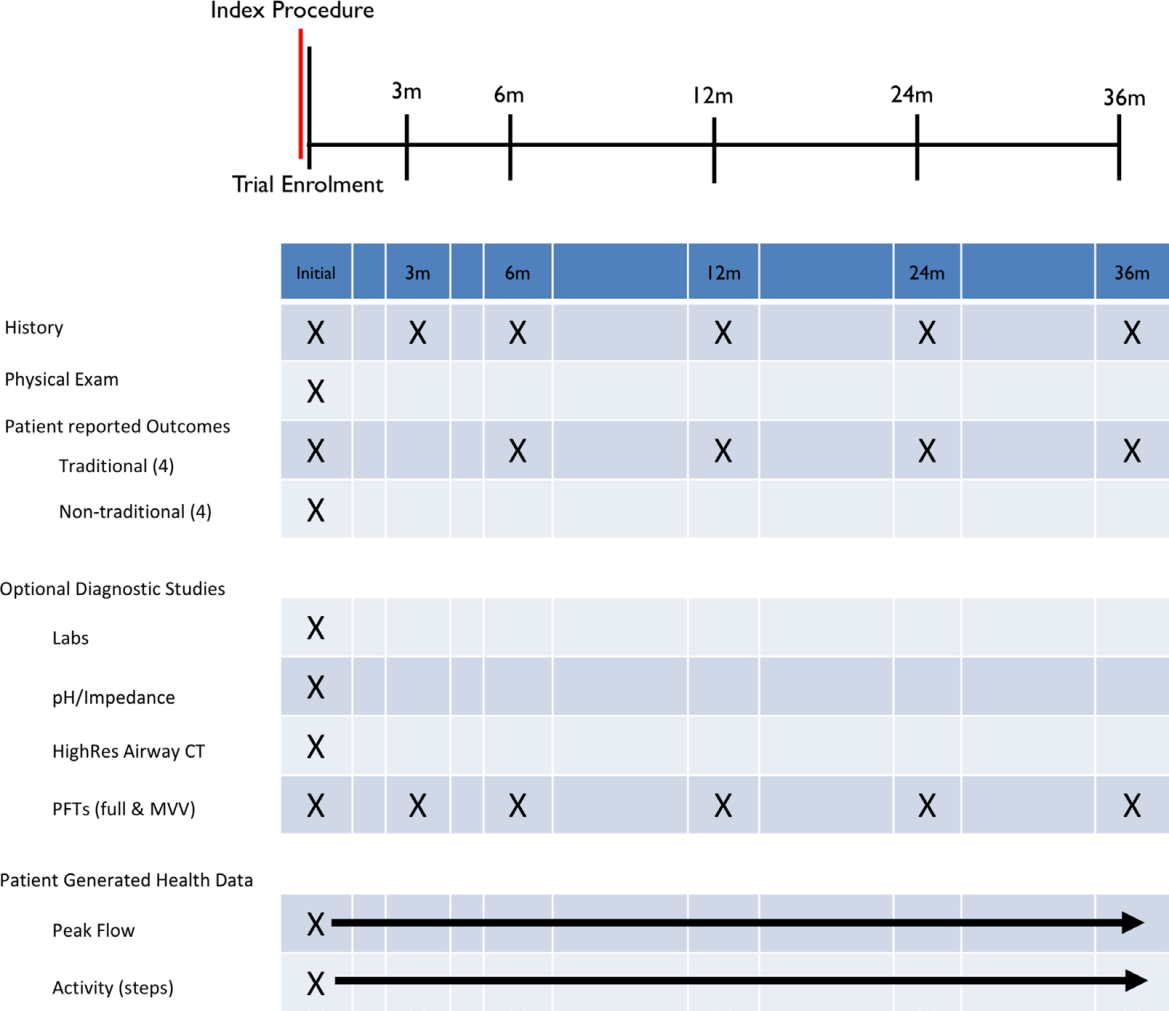
NoAAC PR02 time schedule of enrolment, interventions and assessments and visits for participants. NoAAC PR02, North American Airway Collaborative PR-02; PFT, pulmonary function test.

NoAAC PR02 time schedule of enrolment, interventions and assessments and visits for participants (schematic diagram: figure 4).

### Sample size

The primary endpoint of this trial is TTR. The sample size estimation is completed using the 95% CI method. With the proposed sample size of 300 (endoscopic therapy≈180, open surgery≈60 and endoscopic resection≈60), the half-width of the 95% CI for the TTR function will be less than 0.25 for endoscopic resection and open surgery groups and will be less than 0.15 for the endoscopic dilation group.

### Recruitment

Study participants will be recruited via several mechanisms. Direct recruitment by participating clinician providers that diagnose and treat these patients will occur at each of the participating centres. It is expected that the majority of patients will be recruited through this approach. Direct recruitment will also be accomplished via partnered social media-based advocacy groups in Facebook and Yahoo. Interested stakeholders can also identify this study through its registration with ClinicalTrials.gov and the National Organization for Rare Disorders (NORD) rare disease database. Once consented and enrolled, patients will undergo standard of care treatment at the respective centre and will be followed longitudinally for symptom changes, need for further treatment, complications and will have PROs administered at a priori determined intervals. Given the overall enthusiasm within the iSGS community for our study and the lack of any other clinical studies in iSGS (indicating an absence of study fatigue), we anticipated a 90%–95% response rate for all interval data collections.

## Methods: data collection, management and analysis

### Data collection methods

**Figure 5:**
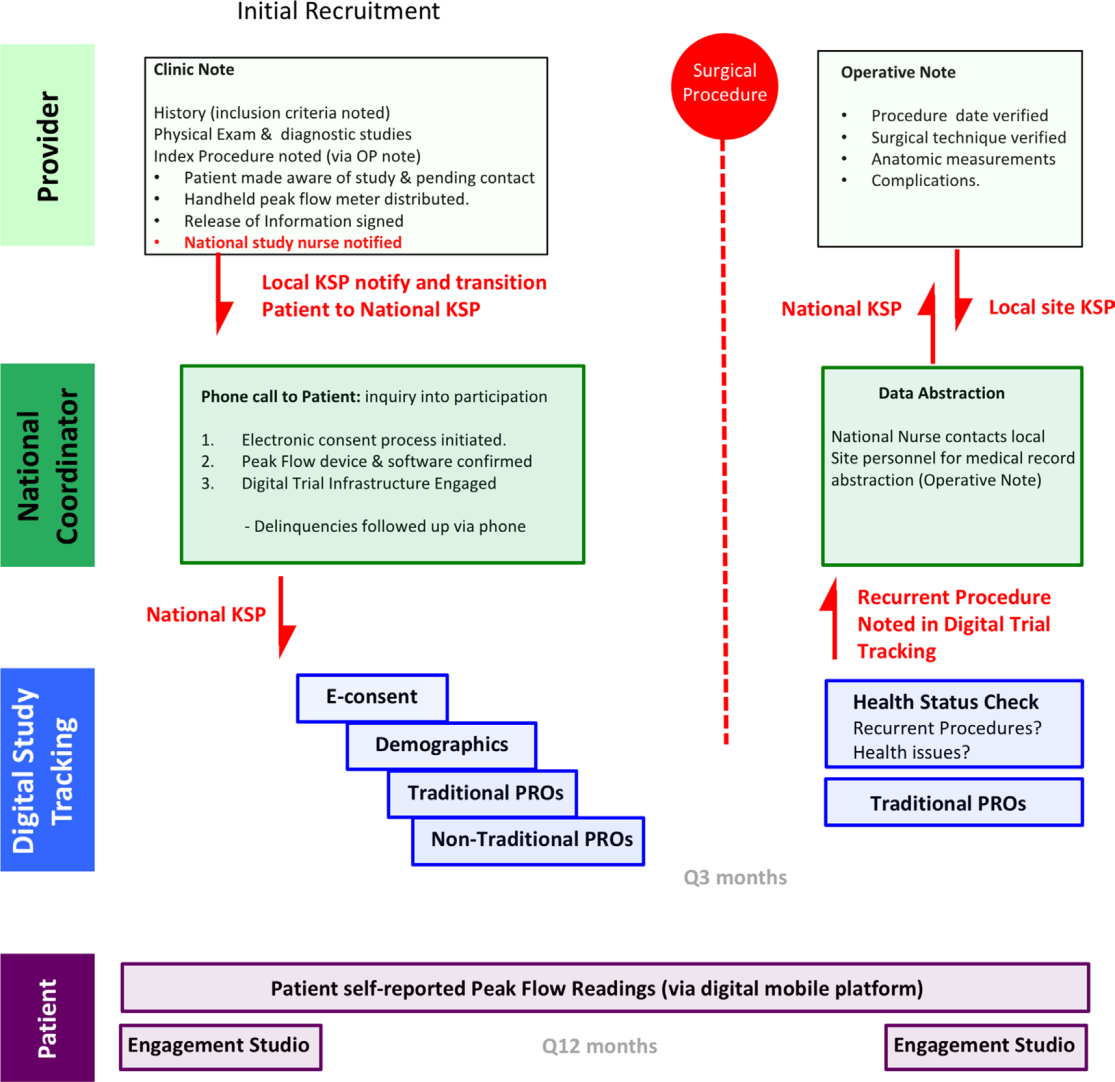
Schematic overview of NoAAC PR02 trial workflow. Data collection for the proposed clinical cohort study will include the CRFs, implemented in an EDC system. CRFs, case report forms; EDC, electronic data capture; NoAAC PR02, North American Airway Collaborative PR-02; PROs, patient-reported outcomes.

Data collection for the proposed clinical cohort study will include the following case report forms (CRFs), implemented in an electronic data capture (EDC) system. EDC development and implementation details are given below (figure 5).

- *Baseline:* At initial patient presentation, baseline data collected will include demographics, medical and surgical history, physical examination findings and relevant diagnostics (eg, CT scan findings, GI study values, laboratory values). Relevant to this study and this disease process will be reproductive, rheumatological and immunological history. These data will involve detailed medical record abstraction, which will be performed by the national nurse coordinator. Quality and accuracy will be ensured double-entering 5% of charts and reviewing results with the lead investigators to ensure data robustness.
- *Procedure:* Details of most recent surgical procedure (ie, *Index procedure*) will be captured in brief; data elements will include date of procedure (which can predate trial enrolment), institution at which procedure was performed, operative findings (eg, site and degree of narrowing within the trachea), degree of and indicator of any deviations from local site protocol (ie, local standard treatment approach).
- *Recurrence:* At patient recurrence (identified directly by patients on recurring scheduled automated follow-up questionnaire or by communication from local site investigators), a minimum subset of features captured at baseline will be captured again; in addition, the details of the repeat procedure(s) will be abstracted from local operative report into the EDC and captured as for the initial procedure.
- *Patient-reported outcomes (PROs):* Four ‘traditional’ validated PRO instruments will be used to assess patient’s QOL. These relate to voice (VHI-10), dysphagia (EAT-10), breathing (COPD dyspnoea) and general QOL (SF-12). Additionally, four ‘non-traditional’ PROs focused on 1) social support,^17^ participatory decision-making style,^18^ disease anxiety and burden,^19^ and fear of disease recurrence^20^ will also be administered at the initial visit. Patients will be asked to complete ‘traditional’ and ‘non-traditional PROs at baseline. The ‘traditional’ PROs will be repeated at routine intervals postprocedure (eg, at 6, 12, 18, 24 and 36 months). For patients who confirm reliable internet access and email connectivity, interval PRO completion will be done directly by the patient into the web-based data capture instrument, with automated email reminders to patients at each PRO interval. For patients without internet access, completion of PROs will be via mailed paper forms or over the phone with a nurse coordinator; the nurse coordinator then will transfer PRO data from paper to the EDC. In all cases, patient failure to complete a required PRO in a timely manner will prompt an automated email to the nurse coordinator for an escalation of follow-up to minimise missing data.

### Harmonisation of datasets and use of CDEs

All data will be collected prospectively; therefore, harmonisation of existing datasets will not be necessary. Instead, common data elements (CDEs) to be collected across participating sites will be developed; the following items of documentation will be developed to ensure clarity of CDEs and comparable data collection across sites.

- *Workflow document:* The workflow document provides a graphical representation of the timeline and logic of data collection as well as data relationships to be modelled in the EDC system (figure 5).
- *Data dictionary:* The data dictionary describes the database structure and details each data element, including variable name, label, graphical-user interface (GUI) format, value domain, definition and any custom notes for implementation.
- *GUI mock-ups:* Prior to initiating development of the web-based data capture application, mock-ups of the GUI are developed for review with system end-users and other appropriate stakeholders. Although the GUI layout of the EDC may be inferred from the data dictionary, the visual mock-up can provide a more accessible point of review for users and allow for fine-tuning of project features.
- *User manual:* A detailed user manual will be developed for the data capture application. The manual will include step-by-step screenshots to guide users through data entry procedures, with highlighted tips for most efficient use of the system.
- *Video training:* A screencast also will be developed, as a live demonstration of data entry procedures.
- *Auxiliary documentation:* Auxiliary documentation includes description of user roles/permissions, detailed descriptions of unusually complex functionalities or similar custom documentation. In addition, all system testing and debugging activity is documented on password-protected electronic boards accessible only to our development team.

### Data entry, collection and quality control

Data collection and management for the proposed project will be performed via a custom web-based EDC system, implementing each of the CRFs described above in electronic format (eCRFs) and built using the Ruby on Rails (RoR) platform. RoR is an open-source web application development framework for creating rich internet applications that model complex data and reinforce data integrity through custom validations. The RoR framework for web-based data entry will be linked to a MySQL database for the data storage component. All data entry interfaces will use dropdown menus, radio buttons/checkboxes and other structured variable formats whenever feasible, to enforce consistency of variable values in data entry. Also, to support data quality control, extensive validations will be developed; these validations check records for internal consistency, conformity to any prespecified data ranges as well as compliance with any known intervariable relationships. Support for longitudinal data will be provided through the use of a relational database architecture that models one-to-many relationships.

In addition to the use of structured variables and validations to prevent data entry errors, other mechanisms for data quality control include:

- *Testing:* The EDC will be tested extensively in both the development/staging and production environments. Automated testing is performed via scripted tests, to verify that observed behaviour of the system conforms to expected behaviour. Manual testing of all system features will also be employed via the user interface and performed by dedicated testing personnel in order to identify (1) bugs, (2) suboptimal feature performance and (3) other issues that may negatively impact the user experience and/or integrity of the data collected. Testing results are documented via a standard operating procedure, in which bugs or other comments are posted to a web-based electronic board; each bug is then tracked on this board through the process of debugging, retesting in the staging environment, deployment to production and final testing in the production environment; thus, a permanent audit trail of testing is maintained. Additional user-facing testing is accomplished through ad hoc testing sessions, in which a group of volunteers follow a ‘hack-a-thon’ model in a ‘mini-test-a-thon’ session, with the intent to identify any remaining system or data entry loopholes that may allow for entry of nonsensical data or loss of data integrity. All testing results are documented and followed through to resolution of the issue.
- *Access controls:* Access to the EDC will be restricted to authorised personnel. Data entry personnel and other users are provided secure access to the web-based application via standard internet technologies (ie, HTTPS), with tiered access permissions appropriate to their study role; such access is granted only when the following criteria are met: (1) request for access is authorised by a study principal investigator or other designated key study personnel and is accompanied by a designation of the user’s role, to ensure appropriate level of access and (2) the user completes training on system use, which may include a live training provided by our group and/or documented completion of a prerecorded video training. This training helps ensure that all users understand the system and the data elements, to minimise potential for data entry errors. All requests for user access, with documentation of fulfilment of the above criteria, are recorded in the permanent system documentation.
- *User-facing validation feature:* In addition to automated data validations run on submission of data, a user-facing validation feature may be implemented to allow for electronic capture of the completion of manual data review for data monitoring purposes. This feature provides a ‘Validate’ button for each patient record, on a per-form basis; a designated user with appropriate role permissions uses this button to indicate that a form has passed manual review of data entry; the form is then date/time-stamped with the date and time of validation review. If a form is edited postvalidation, the time-stamp of previous validation is retained, but the record returns to an unvalidated state. An accompanying validation report may be generated from the system user interface and provides for rapid identification of which forms have completed validation and which require validation or revalidation; links within the reports take the user directly to the annotated forms.
- *21 CFR part 11 compliance:* Systems designed for primary data capture of clinical trial data are 21 CFR part 11 compliant; all members of the database development team have completed training on the requirements of 21 CFR part 11 for EDC systems.
- *Review of datasets:* Datasets extracted from our data capture systems are subject to a final review at time of extraction for interim review or other analysis, with evaluation of data distributions, any outlier or unexpected values or other indicators that may suggest a need to review specific data points with the submitting user or institution to confirm data accuracy.

Additional key features of the EDC will include

- *Sophisticated query interface for extraction of data of interest for review or analysis:* Our query interface uses a two-part approach that allows authorised users to (1) set filters for extraction of cases that meet criteria of interest and (2) select the data elements to be extracted for all cases meeting the filter criteria. For setting filters, all user-facing database variables populate a dropdown menu for selection of the variable to be filtered once a variable is selected, the value domain for that variable populates a second dropdown menu; these two dropdown menus are linked by selection of the operator of interest (ie, ‘equals’, ‘does not equal’, ‘equals any’, ‘contains’, ‘does not contain’ and so on). Any number of variables may be set as filters, and the search may be set to return the union or intersection set of cases meeting the various criteria. In part 2 of the search interface, the user is presented with a list of all user-facing database variables and selects, via checkbox, all variables of interest. Search results are returned in a downloadable Excel workbook with each relevant database table represented in a separate worksheet, including foreign table keys as needed to link records across tables. This format preserves the relation among tables, while avoiding creation of a single unwieldy table complicated by potential multiplicity of 1-to-n relationships.
- *‘Canned’ reports:* Canned reports for frequently requested summary statistics offer a useful adjunct to the query interface. Canned reports pull data from across database tables and assemble the data into a single unified report, according to the prespecifications for the report. The reporting features calls an R-Sweave^21^ script to assemble the data and generate the summary statistics, then write and output the report to PDF.

### Patient-generated health data

**Figure 6:**
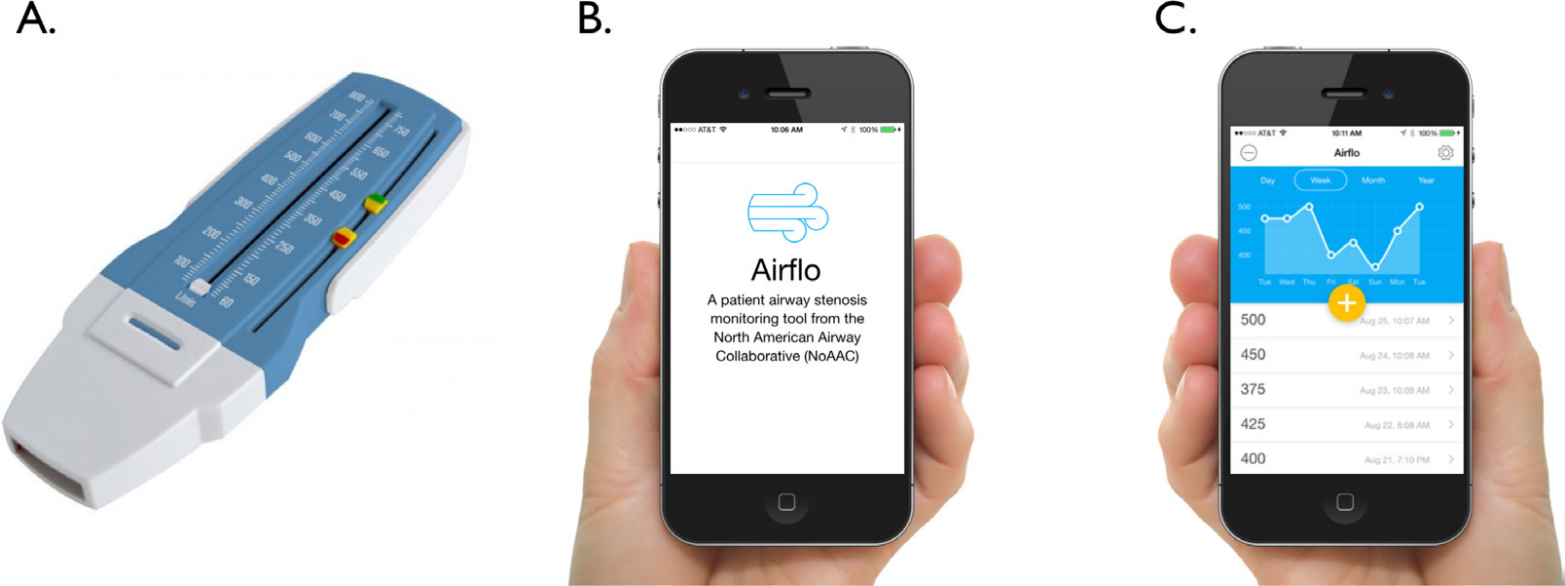
Handheld peak-flow metre (A) along with mobile device software (B) for tracking patient-generated health data (C).

One important element of research is an understanding the experiences of individual patients and one of the ways to learn about those experiences is by collecting patient-generated data. Such information can be obtained in a variety of ways, including during medical visits, through use of smartphones and other electronic devices and as part of research studies. These techniques will be employed during our study to capture patient activity (daily steps) and self-reported peak-flow measurements (figure 6A). An established REDCap-based software platform (figure 6B) will be used to allow patients to input hand-held peak flow data (figure 6C) and provide the ability to track these measurements longitudinally over time.

The REDCap servers are housed in a local data centre at Vanderbilt, and all web-based information transmission is encrypted. REDCap was developed specifically around HIPAA-Security guidelines and is recommended to Vanderbilt researchers by both our Privacy Office and Institutional Review Board. REDCap has been disseminated for local use at more than 940 other academic/non-profit consortium partners in 75 countries. Vanderbilt leads the REDCap Consortium, which currently supports more than 99 000 projects and 128 000 users. More information about the consortium and system security can be found at http://www.projectredcap.org/.

#### Statistical methods

##### The primary endpoint

The primary objective of this study is to evaluate the TTR among three possible treatment groups, that is, endoscopic dilation, endoscopic resection and open surgery.

##### Data analysis plan for the primary endpoint

Demographic information will be tabulated. Descriptive statistics, including means, SD and ranges for continuous parameters as well as per cents and frequencies for categorical parameters will be presented. For the primary objective analysis, that is, estimating the TTR with 95% CI, the TTR time will be estimated using the Kaplan-Meier method with the 95% CI. The Rothman CI, CI’s based on Greenwoods variance, Thomas and Grunkemeier CI and the simultaneous confidence bands by Nair and Hall and Wellner will be reported. The log-rank test will be used to compare the equality of survival curves. The generalised Wilcoxon and log-likelihood tests will also be examined, as these tests weight the survival function differently from the log-rank test, which gives more weight to later occurring events. The Cox proportional hazards model will be applied to investigate potential prognostic factors, such as age on the TTR data. The adjusted p values as well as the adjusted 95% CIs from the Cox model will be reported. The adjusted HRs and 95% CIs will be reported.

##### Secondary endpoints

Differential treatment QOL trade-offs will be prospectively and systematically assessed using both validated ‘traditional’ and ‘non-traditional’ PRO measures. Four ‘traditional’ PROs will measure disability related to voice: VHI-10,^22^ swallowing (EAT-10),^23^ breathing (COPD dyspnoea^24^) and global QOL (SF-12)^25^ (IR4). Four ‘non-traditional’ PROs focused on (1) social support,^17^ participatory decision-making style,^18^ disease anxiety and burden^19^ and fear of disease recurrence^20^ will also be administered at the initial visit. Responses to PROs tend to change in chronic disease states since severity of the measured concept is time-variable. This is particularly true for patients with iSGS whose symptoms markedly improve after treatment and revert and worsen before subsequent treatments. To better understand the breadth of patient experience with this condition, PROs will be employed at a priori determined intervals after diagnosis and treatment, (eg, immediately preintervention, 3, 6 and 12 postintervention), to obtain a more accurate portrait of the survivorship experience related to the different therapeutic modalities.

##### Data analysis plan for secondary endpoints

The secondary objective of this study is to evaluate the QOL scores, for example, SF12, Dyspnoea index, EAT-10 and VHI10, among three study groups. The 95% CI method based on the Normal distribution will be applied to estimate the QOL score among three study groups. The mixed effect model will be applied to examine the correlation between the QOL score and the study groups.

The strategy to be used for developing multivariable models for QOL involves the following steps: (1) Apply multiple imputation for missing covariate values to make good use of partial information. (2) Choose an appropriate statistical model based on the nature of the response variable. (3) Decide on the allowable complexity of the model (ie, the number of covariates) based on the effective sample size available. (4) Allow for non-linear predictor effects using regression splines. (5) Incorporate prespecified interactions. (6) Check distributional assumptions. (7) Adjust the variance–covariance matrix for multiple imputation. (8) Quantify the clinical utility (discrimination ability) of the model. (9) Internally validate the calibration and discrimination of the model using the bootstrap approach, for example, 632+ bootstrap, to estimate the model’s likely performance on a new set of subjects. The statistical analyses will be completed by either R V.3.1.1 or SAS V.9.4 statistical program in this project.

##### Statistical strategy for addressing missing data

Given the enthusiasm of the iSGS population for this trial, we estimate a 90%–95% response rate for the PRO measures in the cohort. Acknowledging this, it is possible that bias could be introduced due to missing data. To account for this, we will use two approaches in cases where participants are alive but are missing PRO data: the multiple imputation model based on the Markov chain Monte Carlo method described above^26^ and a hierarchical hot-deck imputation approach.^27 28^ We will perform sensitivity analyses to compare the two methods to assess the validity of the two approaches. It is necessary to assume that data are missing at random to perform these types of pattern mixture imputation analyses.^28^ In the hierarchical hot-deck imputation approach, participants with missing PRO data will be matched with at least 10 other participants with full data at the previous time point on the following variables: to voice (VHI-10), swallowing (EAT-10), breathing (COPD dyspnoea) and general QOL (SF-12), age, race, comorbid disease, education and income. The order of matching will be determined by the dependent variable being examined. For the purposes of statistical testing, 10 complete data sets will be formed employing the hot-deck imputation approach.^28^ Appropriate survey data analysis techniques will be performed using each data set, leading to 10 separate estimates of parameters and their covariances. We will use the mianalyze.relimp function in R to combine the parameter estimates and obtain covariance estimates which account for both within-imputation and between-imputation sources of variation (*MD-5*). Inferences will be based on the combined parameter estimates and appropriate covariance structure. Our group has used this successfully in prior studies.^29 30^

##### Statistical strategy to address heterogeneity of treatment effect

Subgroup analysis is the most commonly used analytic approach for examining HTE,^31^ and we will use an exploratory variant of this analysis in our approach. Definition of subgroups, endpoints, hypotheses and modelling parameters will be derived in response to the data. An example of this would be the use of a backward model selection approach to identify treatment by covariate interactions. Some of the important types of subgroup variables will be (1) demographic variables (eg, age), (2) pathophysiological variables (eg, timing after recurrence, disease grade), (3) comorbidities (eg, presence of diabetes) and (4) concomitant exposures (eg, hormone replacement therapy, proton pump inhibitors). Additionally, ‘non-traditional’ characteristics that affect patient decision-making (eg, social support, patient decision-making style, disease related anxiety, baseline QOL) will be collected to improve risk-adjustment and increase the individualisation of the results. Although it is extremely difficult to obtain the sampling properties of subgroup effect estimators (eg, SEs), posthoc exploratory subgroup analyses may identify promising hypotheses that will be subject to more rigorous future examination.

##### Statistical strategy to address confounding by selection bias

Observational studies (like our proposed investigation) that lack randomisation of subjects into treatment groups and must address selection bias to properly estimate the effect of treatment. We will apply a propensity scoring method and instrumental variable (IV) method to adjust for observed and unobserved confounding, respectively. Propensity scoring will be used to mitigate the expected biases from observed confounding in the proposed observational study. It is a balancing score that effectively makes the distribution of measured baseline covariates similar between treatment groups. This is important because the apparent difference in outcome between those treated with endoscopic dilation versus those treated with endoscopic resection or open anterior neck surgery may depend on characteristics that affected whether or not a patient received a given treatment instead of due to the actual effect of the treatment. This issue is relevant to our study Aims #2 and #3, but for illustrative purposes, the specific analytic approach will be described in the context of Aim #2. Its objective is to determine factors that affect time to stenosis recurrence in patients with iSGS. In this analysis, the dependent variable is TTR and the primary independent variable is treatment type: endoscopic dilation versus endoscopic excision or open anterior neck surgery of the affected portion of the trachea.

There are three basic techniques for propensity score method: matching, stratification and regression.^32^ We plan to use two-step process of stratification and regression. Stratification consists of grouping subjects into strata determined by observed background characteristics and then comparing subjects between treatment groups directly. Propensity scoring on strata is particularly useful when there are large numbers of covariates, as is the case in this study. Conventionally, creation of five strata has been shown to remove 90% of bias due to the stratified variable.^33 34^ The propensity score is then estimated using a logistic regression model, in which the treatment status is regressed on observed and stratified baseline characteristics. This allows for the formation of matched sets of patients who underwent endoscopic dilation, endoscopic resection or open anterior neck surgery for their iSGS based on similar propensity score values.^35^ In essence, all collected and known confounding covariates will be collectively replaced by a single function of these covariates—the propensity score. Thus, the collection of known predictors is collapsed into a single predictor. Since TTR is a continuous variable, the effect of treatment will be estimated as the difference between the mean time for patients in the endoscopic dilation versus endoscopic resection or open anterior neck surgery groups. Once treatment effect has been estimated using propensity scoring, variance of outcome effect and statistical significance can be estimated. Analysis of the propensity-matched treatment groups can be accomplished by directly comparing outcomes between the three treatment groups. Multivariate regression will be performed to reduce bias due to residual differences in observed baseline covariates between groups.

All known and measurable covariates and confounders will be collected and considered in the propensity score model. Incorporation to the multivariate model will be determined a priori based on their potential to confound or modify the association between treatment and TTR and include *demographics* (age, sex, race, socioeconomic status, geographic location, marital status), *health* (Charlson-Deyo score, body mass index), *endocrinological history* (age of first menses, number of pregnancies, onset of menopause, use of hormone replacement therapy), *inflammatory biomarkers* (high sensitivity CRP, mucosal atopic index), *anatomic* characteristics (degree of luminal obstruction), *physiological* (peak flow rate), *social/behavioural* (Social Support (FSSQ), QOL (SF12), Decision-making style (PDMstyle), Fear of Progression (FoP-Q-SF)). Provider-specific covariates will include the type of subspecialty training programme, training location, procedural volume and treatment criteria for open anterior neck surgery. In addition, the model will include interaction terms for the associations of age with endocrinological history, Charlson-Deyo score and facility regions based on statistical evidence of effect modification and theoretical plausibility.

Propensity scoring can only adjust for observed confounding variables such as those listed above not unobserved ones. Therefore, we will employ quantitative and limited variable (QLIM) analysis to adjust for unobserved confounding. The IV has to meet five specific assumptions: (1) potential outcomes for each patient are unrelated to treatment status of other patients, (2) instrument affects receipt of the treatment of interest, (3) this effect is always in the same direction, (4) instrument assigns treatment randomly and (5) instrument has an effect on the outcome only through the treatment assignment.^36^ The instrument that we plan to use in this analysis is distance from the patient’s residence to the treating facility.^37 38^ The assumption is that the association between distance to the hospital and TTR is due only to the effect of relative distance on treatment assignment after controlling for observed variables and not directly correlated with TTR. This process will involve a two-stage process that first uses the instrument variable and other covariates to predict the treatment. A second stage estimates the outcome by the predicted treatment (from the first model) and other covariates.^39^ Using a two-stage approach has the advantage of incorporating the predicted treatment into the outcome model as it represents the portion of treatment selection related to distance from the treating facility. We will also adjust, when possible, for any instrument-outcome confounding, as confounding can still occur even with the IV procedure.^36^ Other instruments will be considered if distance to treating facility is not found to meet the aforementioned assumptions or is found to significantly confound the outcome.

##### Patient and public involvement

- *Development of the research question and outcome measures*: This patient-motivated and centred investigation involves patient with systematic iSGS and clinician stakeholder engagement, which is required to comprehensively assess iSGS treatment options and their health and QOL trade-offs. We have engaged participants with a lived understanding of the disease to help define its greatest impact on the patient health and QOL. Perspective of patients is often quite discrete from that presumed by their clinicians. Patient and clinician stakeholders have been equally represented in formulating research questions, determining important characteristics affecting patient decisions, defining outcomes, identifying treatments and their potential side-effects and trade-offs. A critical patient partner and co-investigator on this proposal, Catherine Anderson (herself an iSGS patient), has performed groundbreaking work to engage the iSGS community. She founded the a social media based iSGS support group and has used that group as a platform to help understand patient needs and the patient perspective on therapy and subsequent side effects. Her community of patients with iSGS has played an integral role in identifying outcomes that matter to patients: survival, tracheostomy, voice, swallowing and breathing function and HRQOL.
- *Study design:* All member institutions and their associated patient partners have been equal partners in planning the proposed work. Dr Gelbard (PI) from Vanderbilt University (the coordinating institution) has been in frequent personal correspondence with each group at meetings in May and September of 2014. These discussions informed study design, logistics and eligibility requirements. A formal trial-planning meeting was conducted in London England in August 2014 with Catherine in attendance. She re-enforced the need for PRO measures in order to more precisely understand the trade-offs inherent in each therapy (endoscopic dilation, endoscopic resection and open anterior neck surgery).
- *Recruitment:* NoAAC Steering Committee member and co-investigator Catherin Anderson founded and directs the largest social media-based iSGS support group. This resource will provide rapid and inexpensive access to a large audience of patients with potential interest in trial participation and will be used for trial recruitment.
- *Conducting the study:* Several formal and informal channels will be available to patients who wish to provide input to move to the highest level of trial management. This ensures patient engagement with the protocol to optimise patient experience and information gleaned. Specifically, regional patient advocates will report directly to NoAAC steering committee members and through direct feedback via the engaged social media support group (via co-investigator and support group director Catherine Anderson). Furthermore, open communication in expected and encouraged between patient partners and each study site clinician co-investigators to enable clear and consistent mechanism for patient participation and feedback regarding the process.
- *Disseminating study results:* Approximately 2 months before the end of the study timeline, we will have a final team meeting with members the study team and any interested patient partners. The focus will be a discussion of overall study results and on additional strategies to disseminate study results to appropriate scientific, community and stakeholder groups. Results will be published within academic journals and presented at national medical meetings to broaden the scope of dissemination to clinician providers that encounter iSGS. They will also be made publically available through open access publication choices and through patient advocacy sources (eg, Facebook). We plan to use this resource (along with other complementary outlets) for effectively disseminating information about the intervention to the national audience of patients with iSGS.

## Methods: monitoring

### Data monitoring

This is a non-intervention study and there is no data safety monitoring board (DSMB). The principal investigator is responsible for monitoring protocol conduct and reporting any adverse events (AEs) related to study procedures. Given the observational nature of the trial, AEs reporting will include the reporting of any unanticipated patient confidentiality or data security events if it they are deemed probably or definitely related to being in this study. Although AEs are not anticipated on this study, if they should occur, they will be reported to the participating sites IRB board as well as the Vanderbilt Institutional Review Board by the principal investigator.

### Harms

Minimal risk is anticipated. There are no investigational treatments under study in this project. Study patients will be asked to answer questions on self-administered hard copy, electronic, or telephone surveys and self-reported health data (ie, peak flow breathing measurements). Additionally, limited information from their routine medical care will be obtained. Patients will be informed that they can refuse to answer any of the questions. They will also be told that refusal to participate in the study will not in any way change or alter the care they will receive. Patients will be told that survey data are to be obtained for research purposes only and that no individual results will be reported. The data will be kept strictly confidential. No data of any sort will be released to anyone outside the study for any reason. Individual patients are never identified in publications.

Risks to the participants will be minimised by proper screening of potential candidates and strict adherence to confidentiality rules. In addition, access to the EDC system will be strictly restricted to authorised personnel. Data entry personnel and other users are provided secure access to the web-based application via standard internet technologies (ie, HTTPS), with tiered access permissions appropriate to their study role; such access is granted only when the following criteria are met: (1) request for access is authorised by a study principal investigator or other designated key study personnel and is accompanied by a designation of the user’s role, to ensure appropriate level of access and (2) the user completes training on system use, which may include a live training provided by our group and/or documented completion of a prerecorded video training. (3) The user has appropriate training for protection of human subjects. All requests for user access, with documentation of fulfilment of the above criteria, are recorded in the permanent system documentation.

### Auditing

The principle investigator will perform site visits at participating institution during the trial. During these visits, he will interview local site KSP for trial conduct and progress. Additionally, biannual internal audits of data integrity and completeness will be performed and the data coordinating centre. An interim analysis will be performed at 12 months to verify robust data collection.

### Protocol amendments

We plan to communicate important protocol modifications (eg, changes to eligibility criteria, outcomes or analyses) to all relevant parties directly (eg, investigators and trial participants).

### Consent

Local site KSP or the National Nurse Coordinator will obtain informed consent from potential trial participants (online supplementary appendix 2). Additional consent provisions will be explicitly articulated for collection and use of participant data and biological specimens in ancillary studies.

### Confidentiality

How personal information about potential and enrolled participants will be collected, shared and maintained in order to protect confidentiality before, during and after the trial.

### Access to data

The NoAAC study team is committed to promoting the principles of transparency, replication and reproducibility in research. Study documentation (eg, database design, programming code and data definitions) would be shared with requesting researchers and any requesting peer-reviewed journals after PI approval. We will also share all a complete, cleaned, deidentified copy of the final data set with the funding agency (PCORI) and create an access-controlled website for deidentified data abstraction. Access to this resource will require investigator IRB approval along with NoAAC steering committee project approval.

### Ancillary and post-trial care

Given the observational nature of this prospective pragmatic trial, there are not provisions for ancillary and post-trial care.

### Dissemination policy

Team composition was deliberately designed to include individual experts in affected scientific, community and stakeholder groups. Results will be published within academic journals and presented at national medical meetings to broaden the scope of dissemination to clinician providers that encounter iSGS. They will also be made publically available through open access sources and direct delivery to patient advocacy sources (eg, Facebook). As noted by the chief information officer of one of our partnering patient advocacy organisations (the National Organization for Rare Disease: NORD), their organisation will help disseminate results through their website and rare disease database.

### Authorship eligibility guidelines

The formal authorship guidelines of the NoAAC will be followed for this proposal. There is no planned use of professional writers.

## Supplementary Material

Reviewer comments

Author's manuscript

## References

[1] Gelbard A, Donovan DT, Ongkasuwan J, et al Disease homogeneity and treatment heterogeneity in idiopathic subglottic stenosis. Laryngoscope 2016;126:1390–6. 10.1002/lary.25708 26536285 PMC6198250

[2] Gelbard A, Francis DO, Sandulache VC, et al Causes and consequences of adult laryngotracheal stenosis. Laryngoscope 2015;125:1137–43. 10.1002/lary.24956 25290987 PMC4562418

[3] Maldonado F, Loiselle A, Depew ZS, et al Idiopathic subglottic stenosis: an evolving therapeutic algorithm. Laryngoscope 2014;124:498–503. 10.1002/lary.24287 23818139

[4] Ashiku SK, Kuzucu A, Grillo HC, et al Idiopathic laryngotracheal stenosis: effective definitive treatment with laryngotracheal resection. J Thorac Cardiovasc Surg 2004;127:99–107. 10.1016/j.jtcvs.2002.11.001 14752419

[5] Bryans L, Palmer AD, Schindler JS, et al Subjective and objective parameters of the adult female voice after cricotracheal resection and dilation. Ann Otol Rhinol Laryngol 2013;122:707–16. 10.1177/000348941312201108 24358632

[6] Miller CK, Linck J, Willging JP Duration and extent of dysphagia following pediatric airway reconstruction. Int J Pediatr Otorhinolaryngol 2009;73:573–9. 10.1016/j.ijporl.2008.12.024 19203802

[7] Nouraei SA, Sandhu GS Outcome of a multimodality approach to the management of idiopathic subglottic stenosis. Laryngoscope 2013;123:2474–84. 10.1002/lary.23949 23918219

[8] Ettema SL, Tolejano CJ, Thielke RJ, et al Perceptual voice analysis of patients with subglottic stenosis. Otolaryngol Head Neck Surg 2006;135:730–5. 10.1016/j.otohns.2006.06.1249 17071303

[9] Hatcher JL, Dao AM, Simpson CB Voice outcomes after endoscopic treatment of laryngotracheal stenosis. Ann Otol Rhinol Laryngol 2015;124 10.1177/0003489414551980 25301833

[10] Miller CK, Kelchner LN, de Alarcon A, et al Compensatory laryngeal function and airway protection in children following airway reconstruction. Ann Otol Rhinol Laryngol 2014;123:305–13. 10.1177/0003489414525920 24642589

[11] Gelbard A, Francis DO, Sandulache VC, et al Causes and consequences of adult laryngotracheal stenosis. Laryngoscope 2015;125 10.1002/lary.24956 PMC4562418 25290987

[12] Suarez-Almazor ME, Conner-Spady B, Kendall CJ, et al Lack of congruence in the ratings of patients' health status by patients and their physicians. Med Decis Making 2001;21:113–21. 10.1177/02729890122062361 11310944

[13] Hseu AF, Benninger MS, Haffey TM, et al Subglottic stenosis: a ten-year review of treatment outcomes. Laryngoscope 2014;124:736–41. 10.1002/lary.24410 24122779

[14] Dedo HH, Catten MD Idiopathic progressive subglottic stenosis: findings and treatment in 52 patients. Ann Otol Rhinol Laryngol 2001;110:305–11. 10.1177/000348940111000403 11307904

[15] Smith ME, Elstad M Mitomycin C and the endoscopic treatment of laryngotracheal stenosis: are two applications better than one? Laryngoscope 2009;119:272–83. 10.1002/lary.20056 19160408

[16] Taylor SC, Clayburgh DR, Rosenbaum JT, et al Clinical manifestations and treatment of idiopathic and Wegener granulomatosis-associated subglottic stenosis. JAMA Otolaryngol Head Neck Surg 2013;139:76–81. 10.1001/jamaoto.2013.1135 23329095

[17] Sherbourne CD, Stewart AL The MOS social support survey. Soc Sci Med 1991;32:705–14. 10.1016/0277-9536(91)90150-B 2035047

[18] Kaplan SH, Greenfield S, Ware JE Assessing the effects of physician-patient interactions on the outcomes of chronic disease. Med Care 1989;27(3 Suppl):S110–27. 10.1097/00005650-198903001-00010 2646486

[19] Broadbent E, Petrie KJ, Main J, et al The brief illness perception questionnaire. J Psychosom Res 2006;60:631–7. 10.1016/j.jpsychores.2005.10.020 16731240

[20] Mehnert A, Herschbach P, Berg P, et al [Fear of progression in breast cancer patients--validation of the short form of the Fear of Progression Questionnaire (FoP-Q-SF)]. Z Psychosom Med Psychother 2006;52:274–88. 17156600 10.13109/zptm.2006.52.3.274

[21] Sweave FL Dynamic generation of statistical reports using literate data analysis WHaB R, Compstat 2002 - proceedings in computational statistics. Heidelberg: Physica Verlag, 2002:575–80.

[22] Rosen CA, Lee AS, Osborne J, et al Development and validation of the voice handicap index-10. Laryngoscope 2004;114:1549–56. 10.1097/00005537-200409000-00009 15475780

[23] Belafsky PC, Mouadeb DA, Rees CJ, et al Validity and reliability of the Eating Assessment Tool (EAT-10). Ann Otol Rhinol Laryngol 2008;117:919–24. 10.1177/000348940811701210 19140539

[24] Nouraei SAR, Randhawa PS, Koury EF, et al Validation of the Clinical COPD Questionnaire as a psychophysical outcome measure in adult laryngotracheal stenosis. Clinical Otolaryngology 2009;34:343–8. 10.1111/j.1749-4486.2009.01969.x 19673982

[25] Gandek B, Ware JE, Aaronson NK, et al Cross-validation of item selection and scoring for the SF-12 Health Survey in nine countries: results from the IQOLA Project. International Quality of Life Assessment. J Clin Epidemiol 1998;51:1171–8. 9817135 10.1016/s0895-4356(98)00109-7

[26] Fe H Regression modeling strategies, with applications to linear models, survival analysis and logistic regression. New York: Springer, 2001.

[27] Little RJA, Wang Y Pattern-mixture models for multivariate incomplete data with covariates. Biometrics 1996;52:98–111. 10.2307/2533148 8934587

[28] DB.R. Multiple imputation for nonresponse in surveys. New York: John Wiley & Sons, 1987.

[29] Penson DF, McLerran D, Feng Z, et al 5-Year urinary and sexual outcomes after radical prostatectomy: results from the Prostate Cancer Outcomes Study. J Urol 2008;179(5):S40–S44. 10.1016/j.juro.2008.03.136 18405749

[30] PENSON D, McLerran D, Feng Z, et al 5-Year urinary and sexual outcomes after radical prostatectomy: results from the prostate cancer outcomes study. J Urol 2005;173:1701–5. 10.1097/01.ju.0000154637.38262.3a 15821561

[31] Dorresteijn JAN, Visseren FLJ, Ridker PM, et al Estimating treatment effects for individual patients based on the results of randomised clinical trials. BMJ 2011;343:d5888 10.1136/bmj.d5888 21968126 PMC3184644

[32] D’Agostino RB Propensity score methods for bias reduction in the comparison of a treatment to a non-randomized control group. Stat Med 1998;17:2265–81. 10.1002/(SICI)1097-0258(19981015)17:19<2265::AID-SIM918>3.0.CO;2-B 9802183

[33] Rosenbaum PR, Rubin DB Difficulties with regression analyses of age-adjusted rates. Biometrics 1984;40:437–43. 10.2307/2531396 6487727

[34] Cochran WG The effectiveness of adjustment by subclassification in removing bias in observational studies. Biometrics 1968;24:295–313. 10.2307/2528036 5683871

[35] Rosenbaum PR, Rubin DB The bias due to incomplete matching. Biometrics 1985;41:103–16. 10.2307/2530647 4005368

[36] Garabedian LF, Zaslavsky AM, Soumerai SB Instrumental variable analyses for observational comparative effectiveness research: the paired availability design. Ann Intern Med 2014;161:841 10.7326/L14-5029-2 25437418

[37] Rassen JA, Brookhart MA, Glynn RJ, et al Instrumental variables I: instrumental variables exploit natural variation in nonexperimental data to estimate causal relationships. J Clin Epidemiol 2009;62:1226–32. 10.1016/j.jclinepi.2008.12.005 19356901 PMC2905668

[38] Rassen JA, Brookhart MA, Glynn RJ, et al Instrumental variables II: instrumental variable application—in 25 variations, the physician prescribing preference generally was strong and reduced covariate imbalance. J Clin Epidemiol 2009;62:1233–41. 10.1016/j.jclinepi.2008.12.006 19345561 PMC2886011

[39] Angrist JD, Imbens GW, Rubin DB Identification of causal effects using instrumental variables. J Am Stat Assoc 1996;91:444–55. 10.1080/01621459.1996.10476902

