# Supplementary material for: Treatment options in idiopathic subglottic stenosis: protocol for a prospective international multicentre pragmatic trial
Source: BMJ Open. 2018 Apr 10;8(4):e022243. doi: 10.1136/bmjopen-2018-022243 (PMC5898326; doi:10.1136/bmjopen-2018-022243)
Supplement: Supplementary data [file bmjopen-2018-022243supp001.pdf]

Appendix 1.

Data Category	Information
Trial Identifying Number	http://www.clinicalstrials.gov identifier NCT02481817.
Date of Registration	June 25, 2015
Secondary Identifying Numbers	NoAAC PR02
Source of Monetary Support	Patient Centered Outcomes Research Institute (PCORI), Grant ID: 1409-22214
Primary Sponsor	Patient Centered Outcomes Research Institute (PCORI)
Secondary Sponsor	North American Airway Collaborative (NoAAC)
Contact for Public Queries	NoAAC National Trial Coordiator. email: coordinator@noaac.net
Contact for Scientific Queries	NoAAC Director Dr. Alexander Gelbard. email: director@noaac.net
Public Title	Treatment Alternatives in Adult Rare Disease; Assessment of Options in Idiopathic Subglottic Stenosis
Scientific Title	Treatment Alternatives in Adult Rare Disease; Assessment of Options in Idiopathic Subglottic Stenosis North American Airway Collaborative PR-02 Study (NoAAC PR-02 Study)
Countries of Recruitment	International
Health Condition Studied	Idiopatic Subglottic Stenosis (iSGS)
Intervention(s)	Comparison of 3 main surigcal techniques to improve breathing.<br>1. Endoscopic dilation, 2. Endoscopic resection, and 3. Open surgery (tracheal/cricotracheal resection)
Key Inclusion and Exclusion Criteria	Ages eligible for study: ≥18years; Sexes eligible for study:both; Accepts healthy volunteers: no
	Inclusion criteria: Adult patients (≥ 18 years), obstructive airway lesion must involve the subglottis.
	Exclusion criteria: Patients without capacity to consent for themselves. Patients with history endotracheal intubation or tracheotomy within 2 years of first symptoms. Patients with a history of significant laryngotracheal trauma, neck irradiation, caustic or thermal injury to the laryngotracheal complex, or major anterior neck surgery. Patients with a clinical diagnosis of vasculitis or collagen vascular disease, patients with a positive antinuclear cytoplasmic antibody (ANCA) titer.
Study Type	Observational
	Allocation: non-randomized; Intervention model: Pragmatic
	Purpose: Treatment Efficacy
Date of First Enrollment	July 1, 2015
Sample Size	Proposed sample size of 300 (endo dilation ≈ 180, endo resection ≈ 60, open surgery ≈ 60)
Primary Outcome(s)	Treatment effectiveness defined as time to recurrent operative procedure.
Key Secondary Outcomes	Secondary endpoints relate to treatment side effects and include patient reported outcome measures in voice, swallowing, breathing, and global quality of life, as well as patient generated health data.
Ethics Review	This protocol was approved by the IRB Committee of the Vanderbilt University July 2015
