# Supplementary material for: Treatment options in idiopathic subglottic stenosis: protocol for a prospective international multicentre pragmatic trial
Source: BMJ Open. 2018 Apr 10;8(4):e022243. doi: 10.1136/bmjopen-2018-022243 (PMC5898326; doi:10.1136/bmjopen-2018-022243)
Supplement: Supplementary data [file bmjopen-2018-022243supp002.pdf]

NoAAC PR02 - ECONSENT

Study Title: Treatment Alternatives in Adult Rare Disease; Assessment of Options in Idiopathic Subglottic Stenosis (NoAAC PR-02 Study) Version Date 09-15-17

Name of Participant:

____
(First Last)

Age of Participant:

____

1.) What is the purpose of this study?

You are being asked to take part in this research study because you have been diagnosed with Idiopathic Subglottic Stenosis (iSGS). iSGS is a narrowing of your trachea which is the breathing tube from your mouth to your lungs. Your doctor will decide the best course of treatment for you, and we would like to gather information from your chart while you are being treated for this disease. We hope to learn more about common treatments for iSGS, how well they work, and how this disease impacts the quality of your life.

We would also like to collect blood and tissue samples from study patients to help us better understand the causes of iSGS. Our goal is to provide ways to prevent and better manage this disease.

We plan to enroll about 800 people in this study at Vanderbilt. There will be about a total of 1100 people in this study at about 45 sites.

2.) What will happen and how long will you be in the study?

All of the visits and procedures that you have done to treat your disease are part of your regular medical care. They will take place if you are in this study or not. If you agree to be in this study, we will:

** review your medical record to get data about your medical history and health. We will look at information about your diagnosis, tests, and treatments done for it. We will also ask you some questions to try to understand why iSGS occurs.

** ask you to use a handheld asthma monitoring device at home to record your peak flow measurements each day. We will give you one to use during the study.

** ask you to complete eight surveys at several times during the study. If you agree, you will complete these when you first enter the study, after a procedure, at routine follow-up visits (about every 3 months), and if your disease recurs. These will be done for research only and may take less than one hour of your time. We will pay you for each set of surveys that you complete. The surveys will ask questions about your voice, eating and swallowing, overall health status, breathing, etc. We will talk to you about whether you want to complete these in person, by mail, email, or electronically into the research database.

** contact you to discuss your health status and let you know about any other research studies you might be eligible for.

** ask you to take part in 3 focus group interviews with other study participants. These group interviews will be done one time each year and will take about one hour. A focus group is a small group discussion led by a group leader. The leader will ask questions about what it is like to have iSGS and how it affects your life and give the group time to answer them. We encourage everyone to take part in the discussion. The interview will be tape recorded. We will transfer the audio to written notes and study these notes. We will keep the tapes notes in protected computers at Vanderbilt and only the study doctor and his research team will have access to these files. We will talk to you about this more as we get closer to scheduling these interviews.

** ask if we can collect and run tests on some of the tissue from your airway. This would only be done when you are having a biopsy or surgical procedure for your disease. At that time, we would get a small piece of excess tissue study it in the lab.

** ask if we can collect and run tests on your blood. If you agree, we will take about 2.5 tablespoons of blood from a vein in your arm using a needle, at a time when you are having blood drawn for other routine reasons. A research lab will process the blood.

We will record all of this information in a protected database at Vanderbilt, and only the study doctor and his research staff will have access to this database. You will be in the study for about one year.

Genetic/Specimen information

One purpose of this study is to look at genes (DNA) and how they affect health and disease. Genes are the instruction manual for your body. The genes you get from your parents decide what you look like and how your body behaves. They can also tell us a persons risk for certain diseases and how they will respond to treatment.

You are being asked to give blood and tissue samples for genetic research. What we learn about you from these samples will not be put in your health record. Your test results will not be shared with you or your doctor. No one else (like a relative, boss, or insurance company) will be given your test results. Health insurance companies and group health plans may not request your genetic information that comes from this research. Collecting these samples will take about less than 5 minutes of your time.

One risk of giving samples for this research may be the release of your name that could link you to the stored samples and/or the results of the tests run on your samples. This may cause problems with insurance or getting a job.

Health insurance companies and group health plans may not use your genetic information when making decisions regarding your eligibility or premiums. Employers with 15 or more employees may not use your genetic information that comes from this research when making a decision to hire, promote, or fire you or when setting the terms of your employment.

To prevent this, these samples will be given a code. Only the study staff will know the code. The name that belongs to the code will be kept in a locked file or in a computer with a password. Only Dr. Gelbard and his research team will have access to your name.

Your samples will be used to make DNA that will be kept for an unknown length of time (maybe years) for future research. The samples will be destroyed when they are no longer needed.

Your samples may be used to make new products or tests. These may have value and may be developed and owned by the study staff, Vanderbilt University, and/or others. If this happens, there are no plans to provide money to you.

Your samples and information about you may be made available to others to use for research. To protect your privacy, we will not release your name. You will not receive any benefit as a result of the tests done on your samples. These tests may help us learn more about the causes, risks, treatments, or how to prevent this and other health problems. Giving samples for research is your free choice and you may be in the study even if you do not want your samples used or stored for gene research. Giving samples for research is your free choice and you may be in the study even if you do not want your samples used or stored for future research.

At any time, you may ask to have your sample destroyed. You should contact Dr. Gelbard at pager 615-835-9624 to have your sampled destroyed and no longer used for research. We will not be able to destroy research data that has already been gathered using your samples. Also, if your identity was removed from the samples, we will not be able to locate and destroy them.

There will be no costs to you for any of the tests done on your samples. You will not be paid for the use of your samples.

Please check Yes or No to the questions below:

My blood/tissue sample may be used for gene research.	◯ Yes<br>◯ No
My blood/tissue sample may be stored/shared for future gene research in laryngotracheal stenosis disorders.	◯ Yes<br>◯ No
My blood/tissue sample may be stored/shared for future gene research in other health problems (such as cancer, heart disease, etc.)	◯ Yes<br>◯ No

3.) Costs to you if you take part in this study:

There is no cost to you for taking part in this study.

4.) Side effects and risks that you can expect if you take part in this study:

One possible risk to you is the release of information from health records. The chance that this data will be given to someone else by mistake is very small. Ask your doctor for more information about the risks and side effects of procedures that are part of your routine medical care.

Blood samples: You may feel bothered or pained from the needle stick. You may have a bruise or the site may get infected. It is rare, but some people faint.

Additional Information:

5.) Risks that are not known: While we do not know of any risks involved in this study, there may be unknown risks with being in any research study. You will be told of any important findings we learn about related to this study if we think it may change your mind about being in the study.

6.) Payment in case you are injured because of this research study:
If you are injured because you are in this study, you can get reasonable, immediate, and necessary medical care for your injury at Vanderbilt without charge to you. There are no plans for Vanderbilt to pay for the costs of care beyond your injury, or to give you money for such injury.

7.) Good effects that might result from this study:a) The benefits to science and humankind that might result from this study. We hope that we will learn more about the causes for iSGS and better ways to treat people in the future. b) The benefits you might get from being in this study. There is no direct benefit to you from being in this study.

8.) Other treatments you could get if you decide not to be in this study:
This is not a treatment study. If you decide not to be part of the study, it will not affect your regular medical care.

9.) Payments for your time spent taking part in this study or expenses: You will be paid $40 for each set of surveys you complete for being in this study.

10.) Reasons why the study doctor may take you out of this study: There is no reason that the study doctor would take you out of this study.

11.) What will happen if you decide to stop being in this study? Taking part in this study is voluntary. If you decide to stop being part of the study, you should tell the study doctor.

12.) Who to call for any questions or in case you are injured: If you should have any questions about this research study or if you feel you have been hurt by being a part of this study, please feel free to contact Dr. Gelbard at 615-835-9624. If you cannot reach the research staff, please page the study doctor at by calling 615-322-5000 and ask the operator to page him.

For additional information about giving consent or your rights as a person in this study, to discuss problems, concerns, and questions, or to offer input, please feel free to call the Vanderbilt University Institutional Review Board Office at (615) 322-2918 or toll free at (866) 224-8273.

13.) Clinical Trials Registry: A description of this clinical trial will be available on www.clinicaltrials.gov, as required by U.S. Law. This Web site will not include information that can identify you. At most, the Web site will include a summary of the results. You can search this Web site at any time.

Information on Confidentiality, Authorization to Use/Disclose Protected Health Information, and Agreement to Participate in Study.

14.) Confidentiality: You will be assigned a study ID number that will be attached to your records to keep the information confidential. Your records will be kept in a password-protected database. Only the study doctor and his research team will have access to this database.

Vanderbilt may share your information, without identifiers, to others or use it for other research projects not listed in this form. Vanderbilt, Dr. Gelbard and his staff will comply with any and all laws regarding the privacy of such information. There are no plans to pay you for the use or transfer of this de-identified information.

Proceed to review of confidentiality, use/disclosure of protected health information authorization and agreement to participate in study?	◯ Yes<br>◯ No

15.) Authorization to Use/Disclose Protected Health Information

All efforts, within reason, will be made to keep your protected health information (PHI) private. PHI is your health information that is, or has been gathered or kept by Vanderbilt as a result of your healthcare. This includes data gathered for research studies that can be traced back to you. Using or sharing ("disclosure") such data must follow federal privacy rules. By signing the consent for this study, you are agreeing ("authorization") to the uses and likely sharing of your PHI. If you decide to be in this research study, you are also agreeing to let the study team use and share your PHI as described below.

As part of the study, Dr. Gelbard and his study team may share the results of your study and/or non-study linked diagnostic tests, physical exams, operative reports, etc., as well as parts of your medical record, to the groups named below. These groups may include people from the Federal Government Office for Human Research Protections and the Vanderbilt University Institutional Review Board. Federal privacy rules may not apply to these groups; they have their own rules and codes to assure that all efforts, within reason, will be made to keep your PHI private.

The study results will be kept in your research record for at least six years after the study is finished. At that time, the research data that has not been put in your medical record will be kept for an unknown length of time. Any research data that has been put into your medical record will be kept for an unknown length of time.

Unless told otherwise, your consent to use or share your PHI does not expire. If you change your mind, we ask that you contact Dr. Gelbard in writing and let him know that you withdraw your consent. His mailing address is:

Dr. Alexander Gelbard
Vanderbilt Voice Center
Vanderbilt University Medical Center
1215 21st Ave. South
7209 Medical Center East, South Tower
Nashville, TN 37232-8605

At that time, we will stop getting any more data about you. But, the health data we stored before you withdrew your consent may still be used for reporting and research quality.

If you decide not to take part in this research study, it will not affect your treatment, payment or enrollment in any health plans or affect your ability to get benefits. You will get a copy of this form after it is signed.

Statement by person agreeing to be in this study:	◯ I have read this consent form and the research study has been explained to me verbally. All my questions have been answered, and I freely and voluntarily choose to take part in this study.
Patient/Volunteer Name:	______________________________<br>(First Last)

Please provide your signature by choosing one of the two methods below - typing your name in the box, or providing a 'wet' signature.

Participant/Volunteer signature - typed name in box

____
(please type your full name in this box confirming your signature)

Participant/Volunteer 'wet' signature

____
(please provide your signature using your cursor or finger)

Date:

____

Consent obtained by (please enter full name and title):

____
(Completed by study personnel at time of consent.)

Date:

____
