# Supplementary material for: Treatment options in idiopathic subglottic stenosis: protocol for a prospective international multicentre pragmatic trial
Source: BMJ Open. 2018 Apr 10;8(4):e022243. doi: 10.1136/bmjopen-2018-022243 (PMC5898326; doi:10.1136/bmjopen-2018-022243)
Supplement: Supplementary data [file bmjopen-2018-022243supp003.pdf]

Treatment Alternatives in Adult Rare Disease; Assessment of Options in Idiopathic Subglottic Stenosis. North American Airway Collaborative PR-02 Study, A Prospective Pragmatic Trial.

Local Site Investigator Surgical Protocol

NoAAC
the north american airway collaborative

Provider
Name
Academic Rank (*circle one*):
- Instructor
- Assistant Professor
- Associate Professor
- Professor

Institution
Fellowship training (*circle one*):
- Pediatric Oto,
- Laryngology
- Head & Neck
- None

Years out of training (*circle one*):
- 1-5
- 5-10
- >10

Support Staff
Administrative Assistant:

Research Coordinator:

iSGS: Provider Specific Management
Initial Work-up (*circle all that apply*):
- Labs
- PFTs
- GI (pH, impedance and/or endoscopy)
- CT scan

Criteria for intervention (*circle all that apply*):
- Patient Symptoms,
- Physical Exam
- Objective measures
  - (ie. PFTs or Peak Flow meter)

Open Technique:
Number of Open Cases/year ____
Criteria for recommendation for open surgery
Describe:

Surgical Technique (*circle one*):
- Cricotracheal Resection
- Laryngotracheoplasty
- Other

Endoscopic Technique:
Anesthesia (*circle one*):
- Spontaneous Ventilation
- Jet Ventilation
- LMA
- Ventilating Bronchoscope

Scar management (*circle one*):
- CO2 laser incisions
- cold knife
- none
- CO2 laser resection of scar
- other (explain)

Dilation Technique (*circle one*):
- Balloon
- Bougie
- Pediatric Bronchoscope
- No dilation

Adjuvant Therapy (*circle one*):
- PPI
- Inhaled Corticosteroid
- Antibiotics (ie Bactrim or Azithro)
- Other - describe
- Transcutaneous Steroids
- None
