# Supplementary material for: Treatment options in idiopathic subglottic stenosis: protocol for a prospective international multicentre pragmatic trial
Source: BMJ Open. 2018 Apr 10;8(4):e022243. doi: 10.1136/bmjopen-2018-022243 (PMC5898326; doi:10.1136/bmjopen-2018-022243)
Supplement: Author's manuscript [file bmjopen-2018-022243.draft_revisions.pdf]

BMJ Open is committed to open peer review. As part of this commitment we make the peer review history of every article we publish publicly available.

When an article is published we post the peer reviewers' comments and the authors' responses online. We also post the versions of the paper that were used during peer review. These are the versions that the peer review comments apply to.

The versions of the paper that follow are the versions that were submitted during the peer review process. They are not the versions of record or the final published versions. They should not be cited or distributed as the published version of this manuscript.

BMJ Open is an open access journal and the full, final, typeset and author-corrected version of record of the manuscript is available on our site with no access controls, subscription charges or pay-per-view fees (http://bmjopen.bmj.com).

If you have any questions on BMJ Open's open peer review process please email info.bmjopen@bmj.com

BMJ Open

Treatment Options in Idiopathic Subglottic Stenosis: Protocol for a Prospective International Multicenter Pragmatic Trial

Journal:	*BMJ Open*
Manuscript ID	bmjopen-2018-022243
Article Type:	Protocol
Date Submitted by the Author:	12-Feb-2018

Complete List of Authors:	Gelbard, Alexander; Vanderbilt University, Otolaryngology<br>Shyr, Yu; Vanderbilt University,<br>Berry, Lynne; Vanderbilt University, Biostatistics<br>Hillel, Alexander ; Johns Hopkins University, Otolaryngology<br>Ekbom, Dale; Mayo Clinic Department of Otorhinolaryngology<br>Edell, Eric; Mayo Clinic Department of Pulmonology<br>Kasperbauer, Jan; Mayo Clinic Department of Otorhinolaryngology<br>Lott, David; Mayo Clinic Scottsdale, Otorhinolaryngology<br>Donovan, Donald; Baylor College of Medicine Department Otolaryngology<br>Garrett, C. Gaelyn; Vanderbilt University, Otolaryngology<br>Sandhu, Guri; Imperial College Healthcare NHS, Otolaryngology<br>Daniero, James; University of Virginia Health System, Otolaryngology<br>Netterville, James; Vanderbilt University, Otolaryngology<br>Schindler, Josh; Oregon Health and Science University, Otolaryngology<br>Smith, Marshall; University of Utah, Otolaryngology<br>Bryson, Paul; The Cleveland Clinic, Otolaryngology<br>Lorenz, Robert; The Cleveland Clinic, Otolaryngology<br>Francis, David; University of Wisconsin School of Medicine and Public Health, Surgery

Keywords:	Pragmatic Trial, PR02, NoAAC, iSGS, Subglottic Stenosis

SCHOLARONE™
Manuscripts

Treatment Options in Idiopathic Subglottic Stenosis: Protocol for a Prospective International Multicenter Pragmatic Trial

Gelbard A[1], Shyr Y[2], Berry L[2], Hillel AT[3], Ekbom DC[4], Edell ES[4], Kasperbauer JL[4], Lott DG[5], Donovan DT[6], Garrett CG[1], Sandhu G[7], Daniero JJ[8], Netterville JL[1], Schindler JS[9], Smith ME[10], Bryson PC[11], Lorenz RR. [11], Francis DO[12].

Keywords: Pragmatic Trial, PR02, NoAAC, iSGS, Subglottic Stenosis

Word count: 6872

Corresponding Author:
Alexander Gelbard, M.D.
Associate Professor, Department of Otolaryngology, Vanderbilt School of Medicine
Medical Center East, S. Tower.
1215 21st Ave. South, Suite 7302, Nashville, TN 37232-8783.
(alexander.gelbard@vanderbilt.edu)
615.936.4737

Authors:

Alexander Gelbard	[1]Otolaryngology	Vanderbilt University Medical Center	Nashville, TN
Yu Shyr	[2]Biostatistics	Vanderbilt University Medical Center	Nashville, TN
Lynne Berry	[2]Biostatistics	Vanderbilt University Medical Center	Nashville, TN
Alexander Hillel	[3]Otolaryngology	The Johns Hopkins Hospital	Baltimore, MD
Dale Ekbom	[4]Otolaryngology	Mayo Clinic Rochester	Rochester, MN
Eric Edell	[4]Pulmonology	Mayo Clinic Rochester	Rochester, MN
Jan Kasperbauer	[4]Otolaryngology	Mayo Clinic Rochester	Rochester, MN
David Lott	[5]Otolaryngology	Mayo Clinic Scottsdale	Scottsdale, AZ
Donald Donovan	[6]Otolaryngology	Baylor College of Medicine	Houston, TX
Gaelyn Garrett	[1]Otolaryngology	Vanderbilt University Medical Center	Nashville, TN
Guri Sandhu	[7]Otolaryngology	Imperial College Healthcare NHS	London, England
James Daniero	[8]Otolaryngology	University of Virginia Health System	Charlottesville, VA
James Netterville	[1]Otolaryngology	Vanderbilt University Medical Center	Nashville, TN
Joshua Schindler	[9]Otolaryngology	Oregon Health & Science University	Portland, OR
Marshall Smith	[10]Otolaryngology	The University of Utah	Salt Lake City, UT
Paul Bryson	[11]Otolaryngology	Cleveland Clinic	Cleveland, OH
Robert Lorenz	[11]Otolaryngology	Cleveland Clinic	Cleveland, OH
David Francis	[12]Otolaryngology	University of Wisconsin	Madison, WI

For peer review only

Title

Treatment Alternatives in Adult Rare Disease; Assessment of Options in Idiopathic Subglottic Stenosis. North American Airway Collaborative PR-02 Study, A Prospective Pragmatic Trial.

Abstract

Introduction. Idiopathic subglottic stenosis (iSGS) is an unexplained progressive obstruction of the upper airway that occurs almost exclusively in adult, Caucasian women. The disease is characterized by mucosal inflammation and localized fibrosis resulting in life-threatening blockage of the upper airway. Because of high recurrence rates, iSGS patients will frequently require multiple procedures following their initial diagnosis. Both the disease and its therapies profoundly affect patients' ability to breathe, communicate and swallow. A variety of treatments have been advanced to manage this condition. However, comparative data on effectiveness and side effects of the unique approaches have never been systematically evaluated. This study will create an international, multi-institutional prospective cohort of iSGS patients. It will compare 3 surgical approaches to determine how well the most commonly used treatments in iSGS "work"; and what quality of life trade-offs are associated with each approach.

Methods and analysis. A prospective pragmatic trial comparing the "Standard of Care" for iSGS at multiple international institutions. Patients with a diagnosis of iSGS without clinical or laboratory evidence of vasculitis, or a history of endotracheal intubation 2 years prior to symptom onset will be included in the study. Prospective evaluation of disease recurrence requiring operative intervention, validated patient reported outcome measures, as well as patient generated health data (mobile peak flow recordings and daily steps taken) will be longitudinally tracked for 36 months. The primary endpoint is treatment effectiveness defined as time to recurrent operative procedure. Secondary endpoints relate to treatment side effects and include patient reported outcome measures in voice, swallowing, breathing, and global quality of life, as well as patient generated health data.

Ethics and dissemination. This protocol was approved by the local IRB Committee of the Vanderbilt University Medical Center in July 2015. The findings of the trial will be disseminated through peer-reviewed journals, national and international conference presentations and directly to iSGS patient via social media-based support groups.

Registration details. http://www.clinicalstrials.gov identifier NCT02481817.

<u>Strengths of this study</u>

- Unique international prospective cohort of the rare disease Idiopathic Subglottic Stenosis (iSGS)
- Methodology evaluates 3 common surgical approaches in iSGS using objective clinical endpoints to enable assessment of their "real-world" effectiveness.
- Study design also integrates longitudinal assessment of validated patient reported outcome measures to compare patient-perceived functional outcomes of 3 common surgeries in iSGS.

<u>Limitations of this study</u>

- Nonrandomized design
- Adequate statistical power relies on sufficient recruitment which can be a challenge in rare disease.

For peer review only - http://bmjopen.bmj.com/site/about/guidelines.xhtml

Protocol Title: Treatment Alternatives in Adult Rare Disease; Assessment of Options in Idiopathic Subglottic Stenosis. North American Airway Collaborative PR-02 Study, A Prospective Pragmatic Trial.

Trial registration: http://www.clinicalstrials.gov identifier NCT02481817.
World Health Organization Trial Registration Data Set v1.3 (**Appendix 1).**

Protocol version: 05.23.2015, version 2.

Funding: This work was supported by the Patient Centered Outcomes Research Institute (PCORI): Grant ID: 1409-22214

Roles and responsibilities:
Principle Investigators AG & DOF drafted the protocol with methodologic input from AH, JD, DE & PB. YS, and LB were responsible for statistical planning. EE, JK, DD, RL participated in endpoint selection for primary endpoints. DL, GG, JN, JS, GS & MS participated in selection of secondary endpoints and manuscript revision.

Patient Center Outcomes Research Institute (PCORI).www.pcori.com
Dr. Layla Lavasani Ph.D.
Senior Program Officer, Clinical Effectiveness and Decision Science
Patient-Centered Outcomes Research Institute
1919 M St. NW Washington, DC 20036
Office: 202-370-9408
llavasani@pcori.org

Study sponsor and funder had no role (and no ultimate authority) in study design; collection, management, analysis, and interpretation of data; writing of the report; the decision to submit the report for publication, including whether they will have ultimate authority over any of these activities

The composition, roles, and responsibilities of the coordinating center, steering committee, data management team can be found at (https://noaac.net/about/leadership-team/)

For peer review only

A. Introduction.

Background and rationale. Idiopathic subglottic stenosis (iSGS) is an unexplained progressive obstruction of the upper airway that occurs almost exclusively in adult, Caucasian women[1 2]. The disease is characterized by mucosal inflammation and localized fibrosis resulting in life-threatening blockage of the upper airway. Although uncommon (with an estimated incidence of 1:400,000 persons per year[3]), both the disease and its therapies profoundly affect patients' ability to breathe[4], communicate[5] and swallow[6]. Dyspnea is the hallmark symptom and the primary cause of death and disability[7]. However, patients can also experience debilitating voice changes[5 8 9] and swallowing problems[10] due to the condition (**Figures 1A, 1B, & 1C**) or its treatment.

People with this disease often require several surgeries per year[11]. A variety of treatments have been advanced to manage this condition[3 4 11] but are generally categorized into: 1) endoscopic dilation of the tracheal stenosis (accomplished with rigid instruments or inflatable balloons(**Figure 2A**). 2) endoscopic resection of the stenosis (with prolonged medical therapy after surgery: **Figure 2B**), or 3) open surgery with resection of the affected tracheal segment with end-to-end anastomosis (**Figure 2C**). Each patient can require repeated surgeries to keep their trachea open, which increases odds of treatment side effects and complications. All approaches have unique associated side effects, which can significantly affect the patient's quality of life. However, comparative data on trade-offs have never been systematically evaluated.

As a product of disease rarity, there is a lack of high-quality data to inform individual patient decision-making in iSGS. Limited evidence on treatment outcomes complicates patient decision-making as they try to balance survival, symptoms, and quality of life considerations. The proposed prospective study is designed to fill this void and leverages and expands upon a previous retrospective multi-institutional investigation, the North American Airway Collaborative RP-01 (NoAAC RP-01)[1]. In that study, nearly 500 iSGS patients from ten expert centers were retrospectively examined for the need for repeat surgery, and the time to recurrent disease (**Figure 3**). Interestingly, the demographics of this condition were quite similar at the separate centers. iSGS appears to nearly universally affect otherwise healthy, 40 – 60 year old Caucasian women (median age 50.3, 95% CI 49.1 – 51.5)[11] The NoAAC RP-01 study also found variation in the standard of care for iSGS at expert centers. Three basic treatment approaches were used (i.e. endoscopic dilation, endoscopic resection with adjuvant medical therapy, and open surgery) despite an absence of randomized controlled trials (RCTs) or other rigorous comparative studies to assess their differential effectiveness at avoiding disease recurrence. In contrast to the retrospective nature of RP-01, the prospective design of the NoAAC PR-02 study will enable rigorous treatment comparisons to determine how well the most commonly used surgeries in iSGS "work".

While evaluating treatment effectiveness is important, it is equally critical that the patient experience with the disease itself and its treatment be systematically characterized. This is imperative since patient and physician perspectives are often significantly discordant[12]. To this end, NoAAC PR-02 will collect patient-reported outcomes (PROs) in the cohort at initial presentation and at *a priori* determined intervals thereafter, (e.g. immediately pre-intervention, as well as 3, 6, and 12 months post-intervention) and conduct qualitative patient interviews (labeled "engagement studios") to better understand the health-related quality of life (HRQOL) and functional outcomes of therapy in iSGS. These endpoints are important to patients and are arguably a primary determinant in decision-making. For example, results show that endoscopic dilation is associated with a higher rate of disease recurrence and thus need for repeated surgery. Meanwhile, open cricotracheal resection is a major surgery with significant immediate perioperative risks and has been associated with alterations in voice[5 8] and swallowing[6]. Open surgery appears to reduce the risk of disease recurrence, but the degree of benefit, and the trade-offs associated with this invasive surgery are questions that demand prospective study.

Explanation for choice of comparators. The most common contemporary treatments for iSGS will be compared. These treatments (*described previously*) were identified during the preliminary RP-01 study. Patients with this condition have scarring with their trachea and present to the healthcare system with symptoms of airway obstruction (stridor) and respiratory distress. They universally require expeditious intervention to survive. Thus, use of a placebo or "no intervention" control is clinically unethical and inappropriate. The most widely employed treatment is endoscopic dilation of the tracheal scar (60% of patients in RP-01). Patients treated using this approach will be the primary comparator "control" group for this study. Outcomes will be compared to two other interventions: 1) endoscopic resection with adjuvant medical therapy and 2) tracheal resection which comprise approximately 20% of iSGS treatments, respectively.

For peer review only - http://bmjopen.bmj.com/site/about/guidelines.xhtml

Objectives. Specific hypotheses under investigation include:

1. There is variation in time to recurrence (TTR) between centers using similar surgical approaches for iSGS

2. There is variation in TTR between centers using different surgical approaches for iSGS.

3. The different surgical approaches are associated with unique tradeoffs in terms of voice, swallowing, breathing, and global quality of life.

Trial design. Prospective Pragmatic Trial investigating treatment effectiveness in the care of idiopathic subglottic stenosis patients

B. Methods: Participants, interventions, and outcomes

Study setting: International academic medical centers. North American Airway Collaborative Participating Sites can be found at https://noaac.net/about/noaac-sites/. All are academic centers that are tertiary referral centers for iSGS and thus have significant experience treating this rare condition[3 11 13-16]. Nearly all iSGS patients are ultimately referred for care and cluster at such high-volume centers, and thus we anticipate enrolling a representative patient cohort. Each research site has appropriate technological infrastructure for data collection and pathologic specimen tracking.

Eligibility criteria:

Inclusion Criteria:

- >18 years of age.
- The obstructive airway lesion must involve the subglottis.

Exclusion Criteria

- Patients without capacity to consent for themselves
- History of endotracheal intubation or tracheotomy within 2 years of first symptoms.
- History of significant laryngotracheal traumatic injury.
- History of major anterior neck surgery.
- History of neck irradiation.
- History of caustic or thermal injury to the laryngotracheal complex.
- History of a clinically diagnosed vasculitis or collagen vascular disease.
- Positive antinuclear cytoplasmic antibody (ANCA) titers.

Interventions. Once consented (**Appendix 2**) and enrolled, patients will receive standard of care treatment at the respective center (**Appendix 3**). Employing our established digital trial infrastructure patients will be followed longitudinally for symptom changes, need for further treatment, complications, and will have PROs administered at *a priori* determined intervals.

Three interventions being compared include: 1) endoscopic dilation, 2) endoscopic resection of the airway narrowing with subsequent long-term medical therapy, and 3) open neck surgery (**Figure 2**). In endoscopic dilation, the patient undergoes transoral exposure of the tracheal scar with dilation of the scar by either rigid instrument, or controlled radial expansion device (CRE, i.e. balloon dilation). Somewhat similarly, in endoscopic resection the patient undergoes transoral exposure of the tracheal scar, however a CO_2 laser is then utilized to resect a significant portion of the scar, followed by long-term adjuvant medical therapy (Anti-reflux, antibacterial, and inhaled corticosteroid). In open neck surgery (a.k.a tracheal or cricotracheal resection), the trachea is approached via an external incision, the scarred segment of trachea is resected, and the remaining ends sewn back together.

There is significant homogeneity in treatment approach at each participating study. This has been confirmed both through our preliminary RP-01 data and direct correspondence with treating surgeons at these centers. In fact, study sites use a singular surgical approach to treat 95% of their iSGS patients[3]. Personal communications with treating surgeons further indicate that the rationale for consistency in approach is largely experiential. To monitor and ensure consistency, each study site principal co-investigator will submit a

For peer review only - http://bmjopen.bmj.com/site/about/guidelines.xhtml

formalized therapeutic protocol delineating their institutional “standard of care” for iSGS patients to ensure treatment and data homogeneity (**Appendix 3**). Centers are expected adhere to their internal “standard of care” in the management of the iSGS patients. After surgical treatment, national KSP will review the operative report for deviation from the formalized therapeutic protocol (describing how and why the procedure differed from local standard) and enter the results into the digital trial interface.

Outcomes. Interviews with both participating providers and iSGS patients were performed to reach consensus on the primary clinical outcomes to be compared across treatment modalities. Both groups were urged to select outcomes based on what data would be most critical and useful to patients and providers in decision-making. Clinical treatment outcomes identified were: 1) time to recurrence (TTR) or need for repeat procedure to open up their airway obstruction. The specific primary outcome to be measured is time to second treatment (2nd– 1st treatment date). This is an appropriate surrogate measure for recurrence since patient-reported symptoms of respiratory distress are the universal trigger for worsening symptoms, which is nearly perfectly correlated with additional treatment at these study sites. Measuring this outcome is feasible within the 3-year timeframe of the proposed. In our preliminary RP-01 study, recurrence occurred a median 8 months (95% CI 7.2-11.3 months) following initial treatment. It was experienced by 80% of patients. The time to recurrence varied both between iSGS patients receiving the same treatment, and also between the three treatments being prospectively compared in the proposed study (**Figure 3**).

Participant timeline: NoAAC PR02 time schedule of enrolment, interventions, and assessments, and visits for participants. (Schematic diagram: **Figure 4**)

Sample size. The primary endpoint of this trial is time to recurrence. The sample size estimation is completed using the 95% confidence interval (CI) method. With the proposed sample size of 300 (endoscopic therapy ≈ 180, open surgery ≈ 60 and endoscopic resection ≈ 60), the half-width of the 95% confidence interval for the time to recurrence function will be less than .25 for endoscopic resection and open surgery groups, and will be less than .15 for the endoscopic dilation group.

Recruitment. Study participants will be recruited via several mechanisms. Direct recruitment by participating clinician providers that diagnose and treat these patients will occur at each of the participating centers. It is expected that the majority of patients will be recruited through this approach. Direct recruitment will also be accomplished via partnered social media based advocacy groups in Facebook® and Yahoo®. Interested stakeholders can also identify this study through its registration with ClinicalTrials.gov, and the National Organization for Rare Disorders (NORD) rare disease database. Once consented and enrolled, patients will undergo standard of care treatment at the respective center and will be followed longitudinally for symptom changes, need for further treatment, complications, and will have PROs administered at *a priori* determined intervals. Given the overall enthusiasm within the iSGS community for our study, and the lack of any other clinical studies in iSGS (indicating an absence of study fatigue), we anticipated a 90-95% response rate for all interval data collections.

C. Methods: Data collection, management, and analysis

Data collection methods. Data collection for the proposed clinical cohort study will include the following case report forms (CRFs), implemented in an electronic data capture (EDC) system. EDC development and implementation details below (**Figure 5**).

- Baseline. At initial patient presentation, baseline data collected will include demographics, medical and surgical history, physical exam findings, and relevant diagnostics (e.g., CT scan findings, GI study values, laboratory values). Relevant to this study and this disease process will be reproductive, rheumatologic, and immunologic history. These data will involve detailed medical record abstraction, which will be performed by the national nurse coordinator. Quality and accuracy will be ensured double-entering 5% of charts and reviewing results with the lead investigators to ensure data robustness.
- Procedure. Details of most recent surgical procedure (ie. *Index procedure*) will be captured in brief; data elements will include date of procedure (which can predate trial enrollment), institution at which procedure was performed, operative findings (e.g. site and degree of narrowing within the trachea), degree of and indicator of any deviations from local site protocol (ie. local standard treatment approach).
- Recurrence. At patient recurrence (identified directly by patients on recurring scheduled automated follow-up questionnaire, or by communication from local site investigators), a minimum subset of features captured at baseline will be captured again; in addition, the details of the repeat procedure(s) will be abstracted from local operative report into the EDC and captured as for the initial procedure.

For peer review only - http://bmjopen.bmj.com/site/about/guidelines.xhtml

- Patient-reported outcomes (PROs). Four "traditional" validated PRO instruments will be utilized to assess patient quality of life. These relate to voice (VHI-10), dysphagia (EAT-10), breathing (COPD dyspnea), and general quality of life (SF-12). Additionally 4 "Non-traditional" PROs focused on 1) social support[17], participatory decision-making style[18], disease anxiety and burden[19] and fear of disease recurrence[20] will also be administered at the initial visit. Patients will be asked to complete "traditional" and "non-traditional PROs at baseline. The "traditional" PROs will be repeated at routine intervals post-procedure (e.g., at 6, 12, 18, 24 & 36 months). For patients who confirm reliable Internet access and email connectivity, interval PRO completion will be done directly by the patient into the web-based data capture instrument, with automated email reminders to patients at each PRO interval. For patients without Internet access, completion of PROs will be via mailed paper forms, or over the phone with a nurse coordinator; the nurse coordinator then will transfer PRO data from paper to the EDC. In all cases, patient failure to complete a required PRO in a timely manner will prompt an automated email to the nurse coordinator for an escalation of follow-up to minimize missing data.

Harmonization of datasets and use of CDEs: *All data will be collected prospectively; therefore, harmonization of existing datasets will not be necessary. Instead, common data elements (CDEs) to be collected across participating sites will be developed; the following items of documentation will be developed to ensure clarity of CDEs and comparable data collection across sites.*

- Workflow document. The workflow document provides a graphical representation of the timeline and logic of data collection, as well as data relationships to be modeled in the electronic data capture system (Figure 5).
- Data dictionary. The data dictionary describes the database structure, and details each data element, including variable name, label, graphical-user interface (GUI) format, value domain, definition, and any custom notes for implementation.
- GUI mock-ups. Prior to initiating development of the web-based data capture application; mock-ups of the graphical user interface are developed for review with system end-users and other appropriate stakeholders. Although the GUI layout of the EDC may be inferred from the data dictionary, the visual mock-up can provide a more accessible point of review for users, and allow for fine-tuning of project features.
- User manual. A detailed user manual will be developed for the data capture application. The manual will include step-by-step screenshots to guide users through data entry procedures, with highlighted tips for most efficient use of the system.
- Video training. A screencast also will be developed, as a live demonstration of data entry procedures.
- Auxiliary documentation. Auxiliary documentation includes description of user roles/permissions, detailed descriptions of unusually complex functionalities, or similar custom documentation. In addition, all system testing and debugging activity is documented on password-protected electronic boards accessible only to our development team.

Data entry, collection, and quality control

Data collection and management for the proposed project will be performed via a custom web-based electronic data capture system (EDC), implementing each of the CRFs described above in electronic format (eCRFs), and built using the Ruby on Rails (RoR) platform. RoR is an open-source web application development framework for creating rich Internet applications that model complex data and reinforce data integrity through custom validations. The RoR framework for web-based data entry will be linked to a MySQL database for the data storage component. All data entry interfaces will utilize dropdown menus, radio buttons/checkboxes, and other structured variable formats whenever feasible, to enforce consistency of variable values in data entry. Also to support data quality control, extensive validations will be developed; these validations check records for internal consistency, conformity to any pre-specified data ranges, as well as compliance with any known inter-variable relationships. Support for longitudinal data will be provided through the use of a relational database architecture that models one-to-many relationships.

In addition to the use of structured variables and validations to prevent data entry errors, other mechanisms for data quality control include:

- Testing. The EDC will be tested extensively in both the development/staging and production environments. Automated testing is performed via scripted tests, to verify that observed behavior of the system conforms to expected behavior. Manual testing of all system features will also be employed via the user interface and performed by dedicated testing personnel in order to identify 1) bugs, 2) sub-optimal feature performance, and 3) other issues that may negatively impact the user experience and/or integrity of the data collected. Testing results are documented via a standard operating procedure, in which bugs or other comments are posted to a web-based electronic board; each bug is then tracked on this board through the process of debugging, re-testing in the staging environment, deployment to production, and final testing in the

For peer review only - http://bmjopen.bmj.com/site/about/guidelines.xhtml

production environment; thus, a permanent audit trail of testing is maintained. Additional user-facing testing is accomplished through ad hoc testing sessions, in which a group of volunteers follow a 'hack-a-thon' model in a 'mini-test-a-thon' session, with the intent to identify any remaining system or data entry loopholes that may allow for entry of nonsensical data or loss of data integrity. All testing results are documented and followed through to resolution of the issue.

- Access controls. Access to the EDC will be restricted to authorized personnel. Data entry personnel and other users are provided secure access to the web-based application via standard Internet technologies (i.e., HTTPS), with tiered access permissions appropriate to their study role; such access is granted only when the following criteria are met: 1) request for access is authorized by a study principal investigator or other designated key study personnel and is accompanied by a designation of the user's role, to ensure appropriate level of access; and 2) the user completes training on system use, which may include a live training provided by our group and/or documented completion of a pre-recorded video training. This training helps ensure that all users understand the system and the data elements, to minimize potential for data entry errors. All requests for user access, with documentation of fulfillment of the above criteria, are recorded in the permanent system documentation.
- User-facing validation feature. In addition to automated data validations run upon submission of data, a user-facing validation feature may be implemented to allow for electronic capture of the completion of manual data review for data monitoring purposes. This feature provides a 'Validate' button for each patient record, on a per-form basis; a designated user with appropriate role permissions uses this button to indicate that a form has passed manual review of data entry; the form is then date/time-stamped with the date and time of validation review. If a form is edited post-validation, the time-stamp of previous validation is retained, but the record returns to an un-validated state. An accompanying validation report may be generated from the system user interface, and provides for rapid identification of which forms have completed validation, and which require validation or re-validation; links within the reports take the user directly to the annotated forms.
- 21 CFR part 11 compliance. Systems designed for primary data capture of clinical trial data are 21 CFR part 11 compliant; all members of the database development team have completed training on the requirements of 21 CFR part 11 for electronic data capture systems.
- Review of datasets. Datasets extracted from our data capture systems are subject to a final review at time of extraction for interim review or other analysis, with evaluation of data distributions, any outlier or unexpected values, or other indicators that may suggest a need to review specific data points with the submitting user or institution to confirm data accuracy.

Additional key features of the EDC will include:

- Sophisticated query interface for extraction of data of interest for review or analysis. Our query interface uses a two-part approach that allows authorized users to (1) set filters for extraction of cases that meet criteria of interest and (2) select the data elements to be extracted for all cases meeting the filter criteria. For setting filters, all user-facing database variables populate a dropdown menu for selection of the variable to be filtered once a variable is selected, the value domain for that variable populates a second dropdown menu; these two dropdown menus are linked by selection of the operator of interest (i.e., 'equals', 'does not equal', 'equals any', 'contains', 'does not contain', etc.). Any number of variables may be set as filters, and the search may be set to return the union or intersection set of cases meeting the various criteria. In part 2 of the search interface, the user is presented with a list of all user-facing database variables, and selects, via checkbox, all variables of interest. Search results are returned in a downloadable Excel workbook with each relevant database table represented in a separate worksheet, including foreign table keys as needed to link records across tables. This format preserves the relation among tables, while avoiding creation of a single unwieldy table complicated by potential multiplicity of 1-to-n relationships.
- "Canned" reports. Canned reports for frequently requested summary statistics offer a useful adjunct to the query interface. Canned reports pull data from across database tables and assemble the data into a single unified report, according to the pre-specifications for the report. The reporting features calls an R-Sweave[21] script to assemble the data and generate the summary statistics, then write and output the report to PDF.

Patient Generated Health Data.
One important element of research is an understanding the experiences of individual patients, and one of the ways to learn about those experiences is by collecting patient-generated data. Such information can be obtained in a variety of ways, including during medical visits, through use of smartphones and other electronic devices, and as part of research studies. These techniques will be employed during our study to capture patient activity (daily steps) and self-reported peak-flow measurements (**Figure 6A**). An established REDCap-based software platform (**Figure 6B**) will be used to allow patients to input hand-held peak flow data (**Figure 6C**) and provide the ability to track these measurements longitudinally over time.

For peer review only - http://bmjopen.bmj.com/site/about/guidelines.xhtml

The REDCap servers are housed in a local data center at Vanderbilt, and all web-based information transmission is encrypted. REDCap was developed specifically around HIPAA-Security guidelines and is recommended to Vanderbilt researchers by both our Privacy Office and Institutional Review Board. REDCap has been disseminated for local use at more than 940 other academic/non-profit consortium partners in 75 countries. Vanderbilt leads the REDCap Consortium, which currently supports more than 99,000 projects and 128,000 users. More information about the consortium and system security can be found at http://www.projectredcap.org/.

Statistical methods

The primary endpoint: The primary objective of this study is to evaluate the time to recurrence (TTR) among three possible treatment groups, i.e., endoscopic dilation, endoscopic resection, and open surgery.

Data analysis plan for the primary endpoint: Demographic information will be tabulated. Descriptive statistics, including means, standard deviations, and ranges for continuous parameters, as well as percents and frequencies for categorical parameters, will be presented. For the primary objective analysis, i.e., estimating the TTR with 95% confidence interval, the TTR time will be estimated using the Kaplan-Meier method with the 95% confidence interval (CI). The Rothman CI, CI's based on Greenwoods variance, Thomas and Grunkemeier CI and the simultaneous confidence bands by Nair and Hall and Wellner will be reported. The logrank test will be used to compare the equality of survival curves. The generalized Wilcoxon and log-likelihood tests will also be examined, as these tests weight the survival function differently from the logrank test, which gives more weight to later occurring events. The Cox proportional hazards model will be applied to investigate potential prognostic factors, such as age on the TTR data. The adjusted p-values as well as the adjusted 95% confidence intervals from the Cox model will be reported. The adjusted hazard ratios and 95% CIs will be reported.

Secondary endpoints: differential treatment quality of life trade-offs will be prospectively and systematically assessed using both validated “traditional” and “non-traditional” patient reported outcome measures (PRO). 4 “Traditional” PRO will measure disability related to voice (VHI-10[22]), swallowing (EAT-10[23]), breathing (COPD dyspnea[24]), and global quality of life (SF-12[25]) (IR4). 4 “Non-traditional” PROs focused on 1) social support[17], participatory decision-making style[18], disease anxiety and burden[19] and fear of disease recurrence[20] will also be administered at the initial visit. Responses to PROs tend to change in chronic disease states since severity of the measured concept is time-variable. This is particularly true for iSGS patients whose symptoms markedly improve after treatment and revert and worsen before subsequent treatments. To better understand the breadth of patient experience with this condition, PROs will employed at a priori determined intervals after diagnosis and treatment, (e.g. immediately pre-intervention, 3, 6, and 12, post-intervention), to obtain a more accurate portrait of the survivorship experience related to the different therapeutic modalities.

Data analysis plan for secondary endpoints: The secondary objective of this study is to evaluate the quality of life (QOL) scores, e.g., SF12, Dyspnea index, EAT-10, and VHI10, among three study groups. The 95% confidence interval (CI) method based on the Normal distribution will be applied to estimate the quality of life score among three study groups. The mixed effect model will be applied to exam the correlation between the quality of life score and the study groups.

The strategy to be used for developing multivariable models for QOL involves the following steps: (1) Apply multiple imputation for missing covariate values to make good use of partial information. (2) Choose an appropriate statistical model based on the nature of the response variable. (3) Decide on the allowable complexity of the model (i.e., the number of covariates) based on the effective sample size available. (4) Allow for nonlinear predictor effects using regression splines. (5) Incorporate pre-specified interactions. (6) Check distributional assumptions. (7) Adjust the variance-covariance matrix for multiple imputation. (8) Quantify the clinical utility (discrimination ability) of the model. (9) Internally validate the calibration and discrimination of the model using the bootstrap approach, e.g., .632+ bootstrap, to estimate the model's likely performance on a new set of subjects. The statistical analyses will be completed by either R 3.1.1 or SAS 9.4 statistical program in this project

Statistical strategy for addressing missing data: Given the enthusiasm of the iSGS population for this trial, we estimate a 90-95% response rate for the patient reported outcome measures in the cohort. Acknowledging this, it is possible that bias could be introduced due to missing data. To account for this, we will use two approaches in cases where participants are alive but are missing PRO data: the multiple imputation model

For peer review only - http://bmjopen.bmj.com/site/about/guidelines.xhtml

based on the Markov chain Monte Carlo method described above[26] and a hierarchical hot-deck imputation approach[27 28]. We will perform sensitivity analyses compare the two methods to assess the validity of the two approaches. It is necessary to assume data are missing at random (MAR) to perform these types of pattern mixture imputation analyses[28]. In the hierarchical hot-deck imputation approach, participants with missing PRO data will be matched with at least 10 other participants with full data at the previous time point on the following variables: to voice (VHI-10), swallowing (EAT-10), breathing (COPD dyspnea), and general quality of life (SF-12), age, race, co-morbid disease, education and income. The order of matching will be determined by the dependent variable being examined. For the purposes of statistical testing, 10 complete data sets will be formed employing the hot-deck imputation approach[28]. Appropriate survey data analysis techniques will be performed using each data set, leading to 10 separate estimates of parameters and their covariances. We will use the mianalyze.relimp function in R to combine the parameter estimates and obtain covariance estimates which account for both within- and between-imputation sources of variation (*MD-5*). Inferences will be based on the combined parameter estimates and appropriate covariance structure. Our group has used this successfully in prior studies[29 30].

Statistical strategy to address heterogeneity of treatment effect: Subgroup analysis is the most commonly used analytic approach for examining HTE[31], and we will utilize an exploratory variant of this analysis in our approach. Definition of subgroups, endpoints, hypotheses, and modeling parameters will be derived in response to the data. An example of this would be the use of a backward model selection approach to identify treatment by covariate interactions. Some of the important types of subgroup variables will be: (1) demographic variables (e.g., age); (2) pathophysiologic variables (e.g., timing after recurrence, disease grade); (3) comorbidities (e.g., presence of diabetes); and (4) concomitant exposures (e.g., hormone replacement therapy, proton pump inhibitors). Additionally, “non-traditional” characteristics that affect patient decision-making (e.g. social support, patient decision-making style, disease related anxiety, baseline quality of life) will be collected to improve risk-adjustment and increase the individualization of the results. Although it is extremely difficult to obtain the sampling properties of subgroup effect estimators (e.g., standard errors), post hoc exploratory subgroup analyses may identify promising hypotheses that will be subject to more rigorous future examination.

Statistical strategy to address confounding by selection bias: Observational studies (like our proposed investigation) that lack randomization of subjects into treatment groups and must address selection bias to properly estimate the effect of treatment. We will apply a propensity scoring method (PSM) and instrumental variable (IV) method to adjust for observed and unobserved confounding, respectively. Propensity scoring will be used to mitigate the expected biases from observed confounding in the proposed observational study. It is a balancing score that effectively makes the distribution of measured baseline covariates similar between treatment groups. This is important because the apparent difference in outcome between those treated with endoscopic dilation versus those treated with endoscopic resection or open anterior neck surgery may depend on characteristics that affected whether or not a patient received a given treatment instead of due to the actual effect of the treatment. This issue is relevant to our study Aims #2 and #3, but for illustrative purposes, the specific analytic approach will be described in the context of Aim #2. Its objective is to determine factors that affect time to stenosis recurrence in patients with idiopathic subglottic stenosis. In this analysis, the dependent variable is time to recurrence and the primary independent variable is treatment type: endoscopic dilation versus endoscopic excision or open anterior neck surgery of the affected portion of the trachea.

There are three basic techniques for propensity score method: matching, stratification, and regression[32]. We plan to use two-step process of stratification and regression. Stratification consists of grouping subjects into strata determined by observed background characteristics; then comparing subjects between treatment groups directly. Propensity scoring on strata is particularly useful when there are large numbers of covariates, as is the case in this study. Conventionally, creation of five strata has been shown to remove 90% of bias due to the stratified variable[33 34]. The propensity score is then estimated using a logistic regression model, in which the treatment status is regressed on observed and stratified baseline characteristics. This allows for the formation of matched sets of patients who underwent endoscopic dilation, endoscopic resection or open anterior neck surgery for their iSGS based on similar propensity score values[35]. In essence, all collected and known confounding covariates will be collectively replaced by a single function of these covariates – the propensity score. Thus, the collection of known predictors is collapsed into a single predictor. Since time to recurrence is a continuous variable, the effect of treatment will be estimated as the difference between the mean time for patients in the endoscopic dilation versus endoscopic resection or open anterior neck surgery groups. Once treatment effect has been estimated using propensity scoring, variance of outcome effect and statistical significance can be estimated. Analysis of the propensity matched treatment groups can be accomplished by directly comparing outcomes between the three treatment groups. Multivariate regression will be performed to reduce bias due to residual differences in observed baseline covariates between groups.

For peer review only - http://bmjopen.bmj.com/site/about/guidelines.xhtml

All known and measurable covariates and confounders will be collected and considered in the propensity score model. Incorporation to the multivariate model will be determined *a priori* based on their potential to confound or modify the association between treatment and time to recurrence, and include **demographics (**age, sex, race, socioeconomic status, geographic location, marital status), **health (**Charlson-Deyo score, body mass index), **endocrinologic history (**age of first menses, number of pregnancies, onset of menopause, use of hormone replacement therapy), **Inflammatory biomarkers** (high sensitivity CRP, mucosal atopic index), **anatomic** characteristics (degree of luminal obstruction), **physiologic** (peak flow rate), **social/behavioral** [Social Support (FSSQ), Quality of life (SF12), Decision-making style (PDMstyle), Fear of Progression (FoP-Q-SF)]. Provider-specific covariates will include the type of subspecialty training program, training location, procedural volume, and treatment criteria for open anterior neck surgery. In addition, the model will include interaction terms for the associations of age with endocrinologic history, Charlson-Deyo score, and facility regions based on statistical evidence of effect modification and theoretical plausibility.

Propensity scoring can only adjust for observed confounding variables such as those listed above not unobserved ones. Therefore, we will employ quantitative and limited variable (QLIM) analysis to adjust for unobserved confounding. The instrumental variable has to meet five specific assumptions: 1) potential outcomes for each patient are unrelated to treatment status of other patients, 2) instrument affects receipt of the treatment of interest, 3) this effect is always in the same direction, 4) instrument assigns treatment randomly, and 5) instrument has an effect on the outcome only through the treatment assignment[36] . The instrument that we plan to use in this analysis is distance from the patient's residence to the treating facility[37 38]. The assumption is that the association between distance to the hospital and time to recurrence is due only to the effect of relative distance on treatment assignment after controlling for observed variables and not directly correlated with time to recurrence. This process will involve a two-stage process that first uses the instrument variable and other covariates to predict the treatment. A second stage estimates the outcome by the predicted treatment (from the first model) and other covariates[39]. Using a two-stage approach has the advantage of incorporating the predicted treatment into the outcome model as it represents the portion of treatment selection related to distance from the treating facility. We will also adjust, when possible, for any instrument-outcome confounding, as confounding can still occur even with the instrumental variable procedure[36]. Other instruments will be considered if distance to treating facility is not found to meet the aforementioned assumptions or is found to significantly confound the outcome.

D. **Methods: Monitoring**

Data monitoring: This is a non-intervention study and there is no DSMB. The Principal Investigator is responsible for monitoring protocol conduct and reporting any adverse events related to study procedures. Given the observational nature of the trial, Adverse Events (AE) reporting will include the reporting of any unanticipated patient confidentiality or data security events if it they are deemed probably or definitely related to being in this study. Although AEs are not anticipated on this study, if they should occur, they will be reported to the participating sites IRB board as well as the Vanderbilt Institutional Review Board by the Principal Investigator.

Harms: Minimal risk is anticipated. There are no investigational treatments under study in this project. Study patients will be asked to answer questions on self-administered hard copy electronic, mailed, or telephone surveys, and self-reported health data (i.e. peak flow breathing measurements). Additionally, limited information from their routine medical care will be obtained. Patients will be informed that they can refuse to answer any of the questions. They will also be told that refusal to participate in the study will not in any way change or alter the care they will receive. Patients will be told that survey data are to be obtained for research purposes only and that no individual results will be reported. The data will be kept strictly confidential. No data of any sort will be released to anyone outside the study for any reason. Individual patients are never identified in publications.

Risks to the participants will be minimized by proper screening of potential candidates and strict adherence to confidentiality rules. In addition, access to the Electronic Data Capture (EDC) system will be strictly restricted to authorized personnel. Data entry personnel and other users are provided secure access to the web-based application via standard Internet technologies (i.e., HTTPS), with tiered access permissions appropriate to their study role; such access is granted only when the following criteria are met: 1) request for access is authorized by a study principal investigator or other designated key study personnel and is accompanied by a designation of the user's role, to ensure appropriate level of access; and 2) the user completes training on system use, which may include a live training provided by our group and/or documented completion of a pre-recorded video training. 3) The user has appropriate training for protection of human subjects. All requests for user access, with documentation of fulfillment of the above criteria, are recorded in the permanent system documentation.

For peer review only - http://bmjopen.bmj.com/site/about/guidelines.xhtml

Auditing: The principle Investigator will perform site visits at participating institution during the trial During these visit, he will interview local site KSP for trial conduct and progress. Additionally, bi-annual internal audits of data integrity and completeness will be performed and the data coordinating center.
An interim analysis will be performed at 12 months to verify robust data collection

E. Ethics and dissemination

Research ethics approval: Central institutional review board (REC/IRB) approval will be obtained from the Vanderbilt University Medical Center.

Protocol amendments: We plan to communicate important protocol modifications (eg, changes to eligibility criteria, outcomes, or analyses) to all relevant parties directly (eg, investigators, and trial participants).

Consent: Local site KSP or the National Nurse Coordinator will obtain informed consent from potential trial participants (**Appendix 2**). Additional consent provisions will be explicitly articulated for collection and use of participant data and biological specimens in ancillary studies.

Confidentiality: How personal information about potential and enrolled participants will be collected, shared, and maintained in order to protect confidentiality before, during, and after the trial

Declaration of interests: Neither the principal investigators, nor the individual site investigators have financial or other competing interests for the proposed trial.

Access to data: The NoAAC study team is committed to promoting the principles of transparency, replication and reproducibility in research. Study documentation (e.g., database design, programming code, and data definitions) would be shared with requesting researchers, and any requesting peer-reviewed journals after PI approval. We will also share all a complete, cleaned, de-identified copy of the final data set with the funding agency (PCORI), and create an access-controlled website for de-identified data abstraction. Access to this resource will require investigator IRB approval along with NoAAC steering committee project approval.

Ancillary and post-trial care: Given the observational nature of this prospective pragmatic trial, there are not provisions for ancillary and post-trial care.

Dissemination policy: Team composition was deliberately designed to include individual experts in affected scientific, community, and stakeholder groups. Results will be published within academic journals and presented at national medical meetings to broaden the scope of dissemination to clinician providers that encounter iSGS. They will also be made publically available through open access sources and direct delivery to patient advocacy sources (e.g. Facebook). As noted by the chief information officer of one of our partnering patient advocacy organizations (the National Organization for Rare Disease: NORD), their organization will help disseminate results through their website and rare disease database. Additionally, one of our NoAAC Steering Committee members (an iSGS patient and co-investigator, Catherin Anderson) founded and directs the largest social media based iSGS support group. This resource allows bi-directional communication, quickly and inexpensively to provide an open channel for patient partner input. We plan to use this strategic resource (along with other complementary outlets) for effectively disseminating information about the intervention to the national audience of iSGS patients.

Authorship eligibility guidelines: The formal authorship guidelines of the North American Airway Collaborative (NoAAC) will be followed for this proposal. There is no planned use of professional writers.

F. Appendices

For peer review only - http://bmjopen.bmj.com/site/about/guidelines.xhtml

Appendix 1: World Health Organization Trial Registration Data Set

Appendix 2: Informed consent materials: Model consent form and other related documentation given to participants.

Appendix 3: Local Site Surgical Protocol: Model protocol form for local participating site surgeons.

Figure Legends:

Figure 1. Diagram of normal trachea (A.), Healthy trachea (B.), & iSGS patient with obstructive tracheal scar (C.).

Figure 2. Treatment approaches for iSGS: 1) endoscopic dilation of the tracheal stenosis (accomplished with rigid instruments or inflatable balloons: A); 2) endoscopic resection of the stenosis (with prolonged medical therapy after surgery: B); or 3) open surgery with resection of the affected tracheal segment with end-to-end anastomosis (C.).

Figure 3. Kaplan Meier Analysis of NoAAC RP01 study results. Percentage of iSGS patients avoiding disease recurrence, stratified by treatment type.

Figure 4. NoAAC PR02 time schedule of enrolment, interventions, and assessments, and visits for participants.

Figure 5. Schematic overview of NoAAC PR02 trial workflow. Data collection for the proposed clinical cohort study will include the case report forms (CRFs), implemented in an electronic data capture (EDC) system.

Figure 6. Handheld peak-flow meter (A.), along with mobile device software (B.), for tracking patient-generated health data (C.).

For peer review only

References:

1. Gelbard A, Donovan DT, Ongkasuwan J, et al. Disease homogeneity and treatment heterogeneity in idiopathic subglottic stenosis. *The Laryngoscope* 2016;126(6):1390-6. doi: 10.1002/lary.25708
2. Gelbard A, Francis DO, Sandulache VC, et al. Causes and consequences of adult laryngotracheal stenosis. *The Laryngoscope* 2015;125(5):1137-43. doi: 10.1002/lary.24956
3. Maldonado F, Loiselle A, Depew ZS, et al. Idiopathic subglottic stenosis: An evolving therapeutic algorithm. *The Laryngoscope* 2014;124(2):498-503. doi: 10.1002/lary.24287 [published Online First: 2013/07/03]
4. Ashiku SK, Kuzucu A, Grillo HC, et al. Idiopathic laryngotracheal stenosis: effective definitive treatment with laryngotracheal resection. *The Journal of thoracic and cardiovascular surgery* 2004;127(1):99-107. doi: 10.1016/j.jtcvs.2002.11.001
5. Bryans L, Palmer AD, Schindler JS, et al. Subjective and objective parameters of the adult female voice after cricotracheal resection and dilation. *The Annals of otology, rhinology, and laryngology* 2013;122(11):707-16. [published Online First: 2013/12/24]
6. Miller CK, Linck J, Willging JP. Duration and extent of dysphagia following pediatric airway reconstruction. *International journal of pediatric otorhinolaryngology* 2009;73(4):573-9. doi: 10.1016/j.ijporl.2008.12.024
7. Nouraei SA, Sandhu GS. Outcome of a multimodality approach to the management of idiopathic subglottic stenosis. *The Laryngoscope* 2013;123(10):2474-84. doi: 10.1002/lary.23949
8. Ettema SL, Tolejano CJ, Thielke RJ, et al. Perceptual voice analysis of patients with subglottic stenosis. *Otolaryngology--head and neck surgery : official journal of American Academy of Otolaryngology-Head and Neck Surgery* 2006;135(5):730-5. doi: 10.1016/j.otohns.2006.06.1249
9. Hatcher JL, Dao AM, Simpson CB. Voice Outcomes After Endoscopic Treatment of Laryngotracheal Stenosis. *The Annals of otology, rhinology, and laryngology* 2014 doi: 10.1177/0003489414551980
10. Miller CK, Kelchner LN, de Alarcon A, et al. Compensatory laryngeal function and airway protection in children following airway reconstruction. *The Annals of otology, rhinology, and laryngology* 2014;123(5):305-13. doi: 10.1177/0003489414525920
11. Gelbard A, Francis DO, Sandulache VC, et al. Causes and consequences of adult laryngotracheal stenosis. *The Laryngoscope* 2014 doi: 10.1002/lary.24956
12. Suarez-Almazor ME, Conner-Spady B, Kendall CJ, et al. Lack of congruence in the ratings of patients' health status by patients and their physicians. *Medical decision making : an international journal of the Society for Medical Decision Making* 2001;21(2):113-21.
13. Hseu AF, Benninger MS, Haffey TM, et al. Subglottic stenosis: a ten-year review of treatment outcomes. *The Laryngoscope* 2014;124(3):736-41. doi: 10.1002/lary.24410
14. Dedo HH, Catten MD. Idiopathic progressive subglottic stenosis: findings and treatment in 52 patients. *The Annals of otology, rhinology, and laryngology* 2001;110(4):305-11.
15. Smith ME, Elstad M. Mitomycin C and the endoscopic treatment of laryngotracheal stenosis: are two applications better than one? *The Laryngoscope* 2009;119(2):272-83. doi: 10.1002/lary.20056

For peer review only

16. Taylor SC, Clayburgh DR, Rosenbaum JT, et al. Clinical manifestations and treatment of idiopathic and Wegener granulomatosis-associated subglottic stenosis. *JAMA otolaryngology-- head & neck surgery* 2013;139(1):76-81. doi: 10.1001/jamaoto.2013.1135
17. Sherbourne CD, Stewart AL. The MOS social support survey. *Social science & medicine* 1991;32(6):705-14.
18. Kaplan SH, Greenfield S, Ware JE, Jr. Assessing the effects of physician-patient interactions on the outcomes of chronic disease. *Medical care* 1989;27(3 Suppl):S110-27.
19. Broadbent E, Petrie KJ, Main J, et al. The brief illness perception questionnaire. *Journal of psychosomatic research* 2006;60(6):631-7. doi: 10.1016/j.jpsychores.2005.10.020
20. Mehnert A, Herschbach P, Berg P, et al. [Fear of progression in breast cancer patients--validation of the short form of the Fear of Progression Questionnaire (FoP-Q-SF)]. *Zeitschrift fur Psychosomatische Medizin und Psychotherapie* 2006;52(3):274-88.
21. F. L. Sweave: Dynamic generation of statistical reports using literate data analysis. . In: Rönz WHaB, ed. Compstat 2002 - Proceedings in Computational Statistics, pages 575-580. Heidelberg: Physica Verlag 2002:pages 575-80.
22. Rosen CA, Lee AS, Osborne J, et al. Development and validation of the voice handicap index-10. *The Laryngoscope* 2004;114(9):1549-56. doi: 10.1097/00005537-200409000-00009
23. Belafsky PC, Mouadeb DA, Rees CJ, et al. Validity and reliability of the Eating Assessment Tool (EAT-10). *The Annals of otology, rhinology, and laryngology* 2008;117(12):919-24.
24. Nouraei SA, Randhawa PS, Koury EF, et al. Validation of the Clinical COPD Questionnaire as a psychophysical outcome measure in adult laryngotracheal stenosis. *Clinical otolaryngology : official journal of ENT-UK ; official journal of Netherlands Society for Oto-Rhino-Laryngology & Cervico-Facial Surgery* 2009;34(4):343-8. doi: 10.1111/j.1749-4486.2009.01969.x
25. Gandek B, Ware JE, Aaronson NK, et al. Cross-validation of item selection and scoring for the SF-12 Health Survey in nine countries: results from the IQOLA Project. International Quality of Life Assessment. *Journal of clinical epidemiology* 1998;51(11):1171-8.
26. FE H. Regression Modeling Strategies, with Applications to Linear Models, Survival Analysis and Logistic Regression. . New York: Springer 2001.
27. Little RJ, Wang Y. Pattern-mixture models for multivariate incomplete data with covariates. *Biometrics* 1996;52(1):98-111.
28. DB. R. Multiple imputation for nonresponse in surveys. . New York: John Wiley & Sons 1987.
29. Penson DF, McLerran D, Feng Z, et al. 5-year urinary and sexual outcomes after radical prostatectomy: results from the Prostate Cancer Outcomes Study. *The Journal of urology* 2008;179(5 Suppl):S40-4. doi: 10.1016/j.juro.2008.03.136
30. Penson DF, McLerran D, Feng Z, et al. 5-year urinary and sexual outcomes after radical prostatectomy: results from the prostate cancer outcomes study. *The Journal of urology* 2005;173(5):1701-5. doi: 10.1097/01.ju.0000154637.38262.3a
31. Dorresteijn JA, Visseren FL, Ridker PM, et al. Estimating treatment effects for individual patients based on the results of randomised clinical trials. *Bmj* 2011;343:d5888. doi: 10.1136/bmj.d5888
32. D'Agostino RB, Jr. Propensity score methods for bias reduction in the comparison of a treatment to a non-randomized control group. *Statistics in medicine* 1998;17(19):2265-81.
33. Rosenbaum PR, Rubin DB. Difficulties with regression analyses of age-adjusted rates. *Biometrics* 1984;40(2):437-43.
34. Cochran WG. The effectiveness of adjustment by subclassification in removing bias in observational studies. *Biometrics* 1968;24(2):295-313.
35. Rosenbaum PR, Rubin DB. The bias due to incomplete matching. *Biometrics* 1985;41(1):103-16.
36. Garabedian LF, Zaslavsky AM, Soumerai SB. Instrumental variable analyses for observational comparative effectiveness research: the paired availability design. *Annals of internal medicine* 2014;161(11):841. doi: 10.7326/L14-5029-2
37. Rassen JA, Brookhart MA, Glynn RJ, et al. Instrumental variables I: instrumental variables exploit natural variation in nonexperimental data to estimate causal relationships. *Journal of clinical epidemiology* 2009;62(12):1226-32. doi: 10.1016/j.jclinepi.2008.12.005

For peer review only

38. Rassen JA, Brookhart MA, Glynn RJ, et al. Instrumental variables II: instrumental variable application-in 25 variations, the physician prescribing preference generally was strong and reduced covariate imbalance. *Journal of clinical epidemiology* 2009;62(12):1233-41. doi: 10.1016/j.jclinepi.2008.12.006
39. Angrist J, G. Imbens, and D. Rubin. Identification of Causal Effects Using Instrumental Variables. *Journal of the American Statistical Association* 1996;91:444-55.

For peer review only

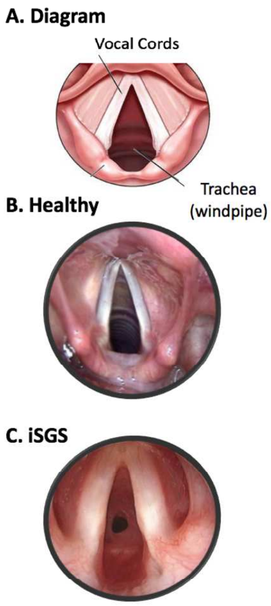

Figure 1. Diagram of normal trachea (A.), Healthy trachea (B.), & iSGS patient with obstructive tracheal scar (C.).

254x190mm (300 x 300 DPI)

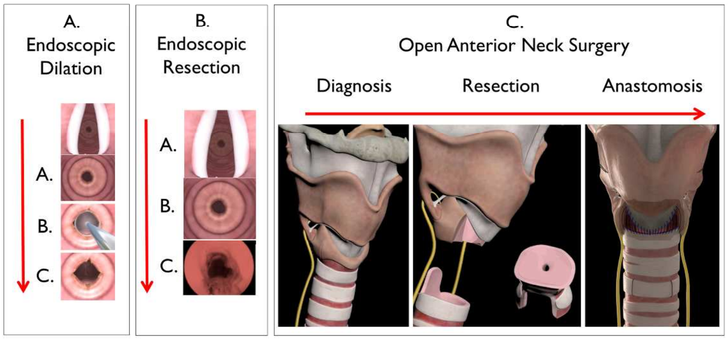

Figure 2. Treatment approaches for iSGS: 1) endoscopic dilation of the tracheal stenosis (accomplished with rigid instruments or inflatable balloons: A); 2) endoscopic resection of the stenosis (with prolonged medical therapy after surgery: B); or 3) open surgery with resection of the affected tracheal segment with end-to-end anastomosis (C.).

254x190mm (300 x 300 DPI)

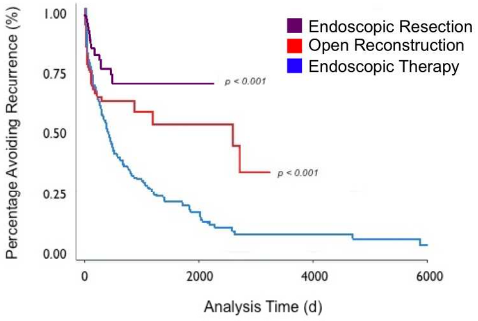

Figure 3. Kaplan Meier Analysis of NoAAC RP01 study results. Percentage of iSGS patients avoiding disease recurrence, stratified by treatment type.

254x190mm (300 x 300 DPI)

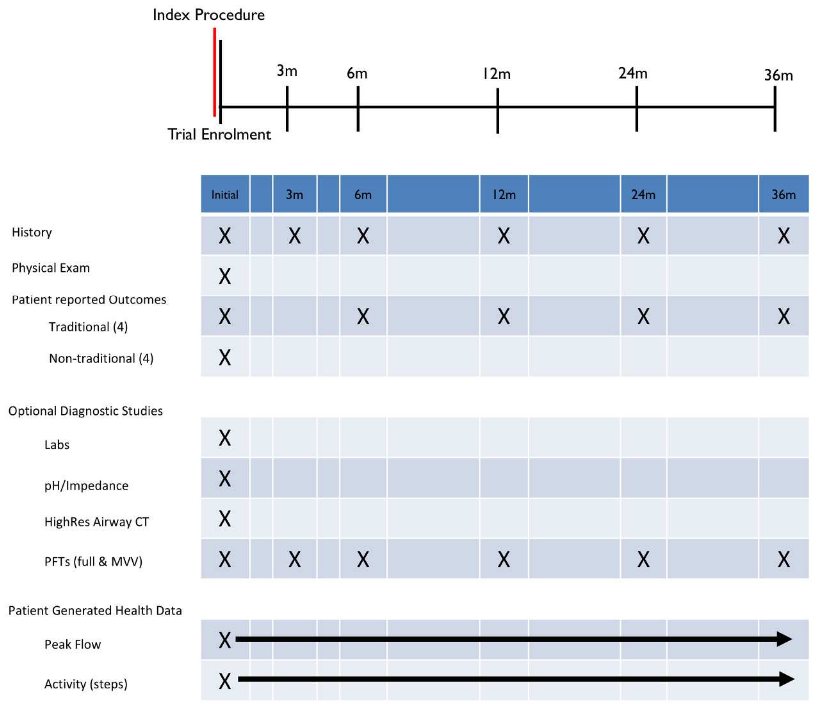

Figure 4. NoAAC PR02 time schedule of enrolment, interventions, and assessments, and visits for participants.

254x190mm (300 x 300 DPI)

w only

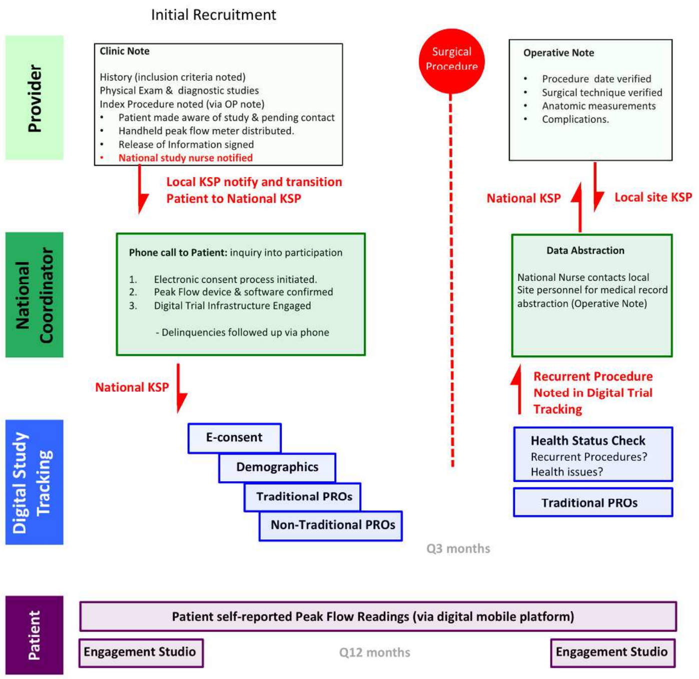

Figure 5. Schematic overview of NoAAC PR02 trial workflow. Data collection for the proposed clinical cohort study will include the case report forms (CRFs), implemented in an electronic data capture (EDC) system.

254x190mm (300 x 300 DPI)

For peer review only - http://bmjopen.bmj.com/site/about/guidelines.xhtml

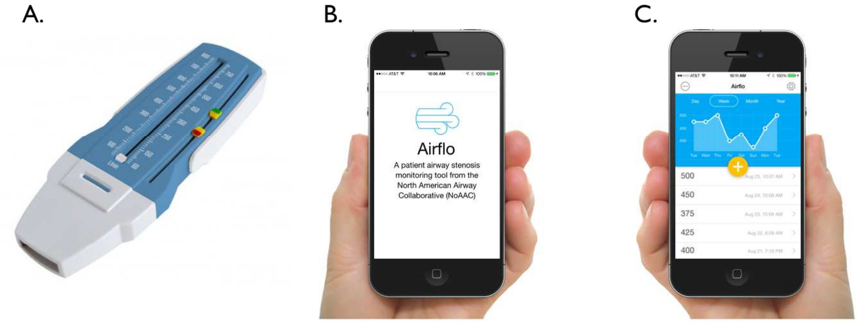

Figure 6. Handheld peak-flow meter (A.), along with mobile device software (B.), for tracking patient-generated health data (C.).

254x190mm (300 x 300 DPI)

w only

Appendix 1.

Data Category	Information
Trial Identifying Number	http://www.clinicalstrials.gov identifier NCT02481817.
Date of Registration	June 25, 2015
Secondary Identifying Numbers	NoAAC PR02
Source of Monetary Support	Patient Centered Outcomes Research Institute (PCORI), Grant ID: 1409-22214
Primary Sponsor	Patient Centered Outcomes Research Institute (PCORI)
Secondary Sponsor	North American Airway Collaborative (NoAAC)
Contact for Public Queries	NoAAC National Trial Coordiator. email: coordinator@noaac.net
Contact for Scientific Queries	NoAAC Director Dr. Alexander Gelbard. email: director@noaac.net
Public Title	Treatment Alternatives in Adult Rare Disease; Assessment of Options in Idiopathic Subglottic Stenosis
Scientific Title	Treatment Alternatives in Adult Rare Disease; Assessment of Options in Idiopathic Subglottic Stenosis North American Airway Collaborative PR-02 Study (NoAAC PR-02 Study)
Countries of Recruitment	International
Health Condition Studied	Idiopatic Subglottic Stenosis (iSGS)
Intervention(s)	Comparison of 3 main surigcal techniques to improve breathing.<br>1. Endoscopic dilation, 2. Endoscopic resection, and 3. Open surgery (tracheal/cricotracheal resection)
Key Inclusion and Exclusion Criteria	Ages eligible for study: ≥18years; Sexes eligible for study:both; Accepts healthy volunteers: no
	Inclusion criteria: Adult patients (≥ 18 years), obstructive airway lesion must involve the subglottis.
	Exclusion criteria: Patients without capacity to consent for themselves. Patients with history endotracheal intubation or tracheotomy within 2 years of first symptoms. Patients with a history of significant laryngotracheal trauma, neck irradiation, caustic or thermal injury to the laryngotracheal complex, or major anterior neck surgery. Patients with a clinical diagnosis of vasculitis or collagen vascular disease, patients with a positive antinuclear cytoplasmic antibody (ANCA) titer.
Study Type	Observational
	Allocation: non-randomized; Intervention model: Pragmatic
	Purpose: Treatment Efficacy
Date of First Enrollment	July 1, 2015
Sample Size	Proposed sample size of 300 (endo dilation ≈ 180, endo resection ≈ 60, open surgery ≈ 60)
Primary Outcome(s)	Treatment effectiveness defined as time to recurrent operative procedure.
Key Secondary Outcomes	Secondary endpoints relate to treatment side effects and include patient reported outcome measures in voice, swallowing, breathing, and global quality of life, as well as patient generated health data.
Ethics Review	This protocol was approved by the IRB Committee of the Vanderbilt University July 2015

NoAAC PR02 - ECONSENT

Study Title: Treatment Alternatives in Adult Rare Disease; Assessment of Options in Idiopathic Subglottic Stenosis (NoAAC PR-02 Study) Version Date 09-15-17

Name of Participant:

____
(First Last)

Age of Participant:

____

1.) What is the purpose of this study?

You are being asked to take part in this research study because you have been diagnosed with Idiopathic Subglottic Stenosis (iSGS). iSGS is a narrowing of your trachea which is the breathing tube from your mouth to your lungs. Your doctor will decide the best course of treatment for you, and we would like to gather information from your chart while you are being treated for this disease. We hope to learn more about common treatments for iSGS, how well they work, and how this disease impacts the quality of your life.

We would also like to collect blood and tissue samples from study patients to help us better understand the causes of iSGS. Our goal is to provide ways to prevent and better manage this disease.

We plan to enroll about 800 people in this study at Vanderbilt. There will be about a total of 1100 people in this study at about 45 sites.

For peer review only

2.) What will happen and how long will you be in the study?

All of the visits and procedures that you have done to treat your disease are part of your regular medical care. They will take place if you are in this study or not. If you agree to be in this study, we will:

** review your medical record to get data about your medical history and health. We will look at information about your diagnosis, tests, and treatments done for it. We will also ask you some questions to try to understand why iSGS occurs.

** ask you to use a handheld asthma monitoring device at home to record your peak flow measurements each day. We will give you one to use during the study.

** ask you to complete eight surveys at several times during the study. If you agree, you will complete these when you first enter the study, after a procedure, at routine follow-up visits (about every 3 months), and if your disease recurs. These will be done for research only and may take less than one hour of your time. We will pay you for each set of surveys that you complete. The surveys will ask questions about your voice, eating and swallowing, overall health status, breathing, etc. We will talk to you about whether you want to complete these in person, by mail, email, or electronically into the research database.

** contact you to discuss your health status and let you know about any other research studies you might be eligible for.

** ask you to take part in 3 focus group interviews with other study participants. These group interviews will be done one time each year and will take about one hour. A focus group is a small group discussion led by a group leader. The leader will ask questions about what it is like to have iSGS and how it affects your life and give the group time to answer them. We encourage everyone to take part in the discussion. The interview will be tape recorded. We will transfer the audio to written notes and study these notes. We will keep the tapes notes in protected computers at Vanderbilt and only the study doctor and his research team will have access to these files. We will talk to you about this more as we get closer to scheduling these interviews.

** ask if we can collect and run tests on some of the tissue from your airway. This would only be done when you are having a biopsy or surgical procedure for your disease. At that time, we would get a small piece of excess tissue study it in the lab.

** ask if we can collect and run tests on your blood. If you agree, we will take about 2.5 tablespoons of blood from a vein in your arm using a needle, at a time when you are having blood drawn for other routine reasons. A research lab will process the blood.

We will record all of this information in a protected database at Vanderbilt, and only the study doctor and his research staff will have access to this database. You will be in the study for about one year.

Genetic/Specimen information

One purpose of this study is to look at genes (DNA) and how they affect health and disease. Genes are the instruction manual for your body. The genes you get from your parents decide what you look like and how your body behaves. They can also tell us a persons risk for certain diseases and how they will respond to treatment.

You are being asked to give blood and tissue samples for genetic research. What we learn about you from these samples will not be put in your health record. Your test results will not be shared with you or your doctor. No one else (like a relative, boss, or insurance company) will be given your test results. Health insurance companies and group health plans may not request your genetic information that comes from this research. Collecting these samples will take about less than 5 minutes of your time.

One risk of giving samples for this research may be the release of your name that could link you to the stored samples and/or the results of the tests run on your samples. This may cause problems with insurance or getting a job.

Health insurance companies and group health plans may not use your genetic information when making decisions regarding your eligibility or premiums. Employers with 15 or more employees may not use your genetic information that comes from this research when making a decision to hire, promote, or fire you or when setting the terms of your employment.

To prevent this, these samples will be given a code. Only the study staff will know the code. The name that belongs to the code will be kept in a locked file or in a computer with a password. Only Dr. Gelbard and his research team will have access to your name.

Your samples will be used to make DNA that will be kept for an unknown length of time (maybe years) for future research. The samples will be destroyed when they are no longer needed.

Your samples may be used to make new products or tests. These may have value and may be developed and owned by the study staff, Vanderbilt University, and/or others. If this happens, there are no plans to provide money to you.

Your samples and information about you may be made available to others to use for research. To protect your privacy, we will not release your name. You will not receive any benefit as a result of the tests done on your samples. These tests may help us learn more about the causes, risks, treatments, or how to prevent this and other health problems. Giving samples for research is your free choice and you may be in the study even if you do not want your samples used or stored for gene research. Giving samples for research is your free choice and you may be in the study even if you do not want your samples used or stored for future research.

At any time, you may ask to have your sample destroyed. You should contact Dr. Gelbard at pager 615-835-9624 to have your sampled destroyed and no longer used for research. We will not be able to destroy research data that has already been gathered using your samples. Also, if your identity was removed from the samples, we will not be able to locate and destroy them.

There will be no costs to you for any of the tests done on your samples. You will not be paid for the use of your samples.

Please check Yes or No to the questions below:

My blood/tissue sample may be used for gene research.	◯ Yes<br>◯ No
My blood/tissue sample may be stored/shared for future gene research in laryngotracheal stenosis disorders.	◯ Yes<br>◯ No
My blood/tissue sample may be stored/shared for future gene research in other health problems (such as cancer, heart disease, etc.)	◯ Yes<br>◯ No

For peer review only

3.) Costs to you if you take part in this study:

There is no cost to you for taking part in this study.

4.) Side effects and risks that you can expect if you take part in this study:

One possible risk to you is the release of information from health records. The chance that this data will be given to someone else by mistake is very small. Ask your doctor for more information about the risks and side effects of procedures that are part of your routine medical care.

Blood samples: You may feel bothered or pained from the needle stick. You may have a bruise or the site may get infected. It is rare, but some people faint.

Additional Information:

5.) Risks that are not known: While we do not know of any risks involved in this study, there may be unknown risks with being in any research study. You will be told of any important findings we learn about related to this study if we think it may change your mind about being in the study.

6.) Payment in case you are injured because of this research study:
If you are injured because you are in this study, you can get reasonable, immediate, and necessary medical care for your injury at Vanderbilt without charge to you. There are no plans for Vanderbilt to pay for the costs of care beyond your injury, or to give you money for such injury.

7.) Good effects that might result from this study:a) The benefits to science and humankind that might result from this study. We hope that we will learn more about the causes for iSGS and better ways to treat people in the future. b) The benefits you might get from being in this study. There is no direct benefit to you from being in this study.

8.) Other treatments you could get if you decide not to be in this study:
This is not a treatment study. If you decide not to be part of the study, it will not affect your regular medical care.

9.) Payments for your time spent taking part in this study or expenses: You will be paid $40 for each set of surveys you complete for being in this study.

10.) Reasons why the study doctor may take you out of this study: There is no reason that the study doctor would take you out of this study.

11.) What will happen if you decide to stop being in this study? Taking part in this study is voluntary. If you decide to stop being part of the study, you should tell the study doctor.

12.) Who to call for any questions or in case you are injured: If you should have any questions about this research study or if you feel you have been hurt by being a part of this study, please feel free to contact Dr. Gelbard at 615-835-9624. If you cannot reach the research staff, please page the study doctor at by calling 615-322-5000 and ask the operator to page him.

For additional information about giving consent or your rights as a person in this study, to discuss problems, concerns, and questions, or to offer input, please feel free to call the Vanderbilt University Institutional Review Board Office at (615) 322-2918 or toll free at (866) 224-8273.

13.) Clinical Trials Registry: A description of this clinical trial will be available on www.clinicaltrials.gov, as required by U.S. Law. This Web site will not include information that can identify you. At most, the Web site will include a summary of the results. You can search this Web site at any time.

For peer review only - http://bmjopen.bmj.com/site/about/guidelines.xhtml

Information on Confidentiality, Authorization to Use/Disclose Protected Health Information, and Agreement to Participate in Study.

14.) Confidentiality: You will be assigned a study ID number that will be attached to your records to keep the information confidential. Your records will be kept in a password-protected database. Only the study doctor and his research team will have access to this database.

Vanderbilt may share your information, without identifiers, to others or use it for other research projects not listed in this form. Vanderbilt, Dr. Gelbard and his staff will comply with any and all laws regarding the privacy of such information. There are no plans to pay you for the use or transfer of this de-identified information.

Proceed to review of confidentiality, use/disclosure of protected health information authorization and agreement to participate in study?

◯ Yes
◯ No

15.) Authorization to Use/Disclose Protected Health Information

All efforts, within reason, will be made to keep your protected health information (PHI) private. PHI is your health information that is, or has been gathered or kept by Vanderbilt as a result of your healthcare. This includes data gathered for research studies that can be traced back to you. Using or sharing ("disclosure") such data must follow federal privacy rules. By signing the consent for this study, you are agreeing ("authorization") to the uses and likely sharing of your PHI. If you decide to be in this research study, you are also agreeing to let the study team use and share your PHI as described below.

As part of the study, Dr. Gelbard and his study team may share the results of your study and/or non-study linked diagnostic tests, physical exams, operative reports, etc., as well as parts of your medical record, to the groups named below. These groups may include people from the Federal Government Office for Human Research Protections and the Vanderbilt University Institutional Review Board. Federal privacy rules may not apply to these groups; they have their own rules and codes to assure that all efforts, within reason, will be made to keep your PHI private.

The study results will be kept in your research record for at least six years after the study is finished. At that time, the research data that has not been put in your medical record will be kept for an unknown length of time. Any research data that has been put into your medical record will be kept for an unknown length of time.

Unless told otherwise, your consent to use or share your PHI does not expire. If you change your mind, we ask that you contact Dr. Gelbard in writing and let him know that you withdraw your consent. His mailing address is:

Dr. Alexander Gelbard
Vanderbilt Voice Center
Vanderbilt University Medical Center
1215 21st Ave. South
7209 Medical Center East, South Tower
Nashville, TN 37232-8605

At that time, we will stop getting any more data about you. But, the health data we stored before you withdrew your consent may still be used for reporting and research quality.

If you decide not to take part in this research study, it will not affect your treatment, payment or enrollment in any health plans or affect your ability to get benefits. You will get a copy of this form after it is signed.

Statement by person agreeing to be in this study:

◯ I have read this consent form and the research study has been explained to me verbally. All my questions have been answered, and I freely and voluntarily choose to take part in this study.

Patient/Volunteer Name:

____
(First Last)

For peer review only

Please provide your signature by choosing one of the two methods below - typing your name in the box, or providing a 'wet' signature.

Participant/Volunteer signature - typed name in box

____
(please type your full name in this box confirming your signature)

Participant/Volunteer 'wet' signature

____
(please provide your signature using your cursor or finger)

Date:

____

Consent obtained by (please enter full name and title):

____
(Completed by study personnel at time of consent.)

Date:

____

For peer review only

Treatment Alternatives in Adult Rare Disease; Assessment of Options in Idiopathic Subglottic Stenosis. North American Airway Collaborative PR-02 Study, A Prospective Pragmatic Trial.

Local Site Investigator Surgical Protocol

NoAAC
the north american airway collaborative

Provider
Name
Academic Rank (*circle one*):
- Instructor
- Assistant Professor
- Associate Professor
- Professor

Institution
Fellowship training (*circle one*):
- Pediatric Oto,
- Laryngology
- Head & Neck
- None

Years out of training (*circle one*):
- 1-5
- 5-10
- >10

Support Staff
Administrative Assistant:

Research Coordinator:

iSGS: Provider Specific Management
Initial Work-up (*circle all that apply*):
- Labs
- PFTs
- GI (pH, impedance and/or endoscopy)
- CT scan

Criteria for intervention (*circle all that apply*):
- Patient Symptoms,
- Physical Exam
- Objective measures
  - (ie. PFTs or Peak Flow meter)

Open Technique:
Number of Open Cases/year ____
Criteria for recommendation for open surgery
Describe:

Surgical Technique (*circle one*):
- Cricotracheal Resection
- Laryngotracheoplasty
- Other

Endoscopic Technique:
Anesthesia (*circle one*):
- Spontaneous Ventilation
- Jet Ventilation
- LMA
- Ventilating Bronchoscope

Scar management (*circle one*):
- CO2 laser incisions
- cold knife
- none
- CO2 laser resection of scar
- other (explain)

Dilation Technique (*circle one*):
- Balloon
- Bougie
- Pediatric Bronchoscope
- No dilation

Adjuvant Therapy (*circle one*):
- PPI
- Inhaled Corticosteroid
- Antibiotics (ie Bactrim or Azithro)
- Other - describe
- Transcutaneous Steroids
- None

For peer review only

For peer review only - http://bmjopen.bmj.com/site/about/guidelines.xhtml

Reporting checklist for protocol of a clinical trial.

Based on the SPIRIT guidelines.

Instructions to authors

Complete this checklist by entering the page numbers from your manuscript where readers will find each of the items listed below.

If your work does not currently contain an item consider adding it. If you are certain that an item does not apply to your work, write 'n/a'. If you like, you can provide a short reason.

Upload this checklist as an extra file when you submit to a journal.

In your methods section, say that you used the SPIRIT reporting guidelines, and cite them as:

Chan A-W, Tetzlaff JM, Altman DG, Laupacis A, Gøtzsche PC, Krleža-Jerić K, Hróbjartsson A, Mann H, Dickersin K, Berlin J, Doré C, Parulekar W, Summerskill W, Groves T, Schulz K, Sox H, Rockhold FW, Rennie D, Moher D. SPIRIT 2013 Statement: Defining standard protocol items for clinical trials. Ann Intern Med. 2013;158(3):200-207

		Reporting Item	Page Number
Title	#1	Descriptive title identifying the study design, population, interventions, and, if applicable, trial acronym	3
Trial registration	#2a	Trial identifier and registry name. If not yet registered, name of intended registry	3
Trial registration: data set	#2b	All items from the World Health Organization Trial Registration Data Set	3
Protocol version	#3	Date and version identifier	3
Funding	#4	Sources and types of financial, material, and other support	3
Roles and responsibilities: contributorship	#5a	Names, affiliations, and roles of protocol contributors	3

For peer review only

Roles and responsibilities: sponsor contact information	#5b	Name and contact information for the trial sponsor	3
Roles and responsibilities: sponsor and funder	#5c	Role of study sponsor and funders, if any, in study design; collection, management, analysis, and interpretation of data; writing of the report; and the decision to submit the report for publication, including whether they will have ultimate authority over any of these activities	3
Roles and responsibilities: committees	#5d	Composition, roles, and responsibilities of the coordinating centre, steering committee, endpoint adjudication committee, data management team, and other individuals or groups overseeing the trial, if applicable (see Item 21a for data monitoring committee)	3
Background and rationale	#6a	Description of research question and justification for undertaking the trial, including summary of relevant studies (published and unpublished) examining benefits and harms for each intervention	4
Background and rationale: choice of comparators	#6b	Explanation for choice of comparators	4
Objectives	#7	Specific objectives or hypotheses	5
Trial design	#8	Description of trial design including type of trial (eg, parallel group, crossover, factorial, single group), allocation ratio, and framework (eg, superiority, equivalence, non-inferiority, exploratory)	5
Study setting	#9	Description of study settings (eg, community clinic, academic hospital) and list of countries where data will be collected. Reference to where list of study sites can be obtained	5
Eligibility criteria	#10	Inclusion and exclusion criteria for participants. If applicable, eligibility criteria for study centres and	5

For peer review only

		individuals who will perform the interventions (eg, surgeons, psychotherapists)	
Interventions: description	#11a	Interventions for each group with sufficient detail to allow replication, including how and when they will be administered	5
Interventions: modifications	#11b	Criteria for discontinuing or modifying allocated interventions for a given trial participant (eg, drug dose change in response to harms, participant request, or improving / worsening disease)	5
Interventions: adherance	#11c	Strategies to improve adherence to intervention protocols, and any procedures for monitoring adherence (eg, drug tablet return; laboratory tests)	6
Interventions: concomitant care	#11d	Relevant concomitant care and interventions that are permitted or prohibited during the trial	6
Outcomes	#12	Primary, secondary, and other outcomes, including the specific measurement variable (eg, systolic blood pressure), analysis metric (eg, change from baseline, final value, time to event), method of aggregation (eg, median, proportion), and time point for each outcome. Explanation of the clinical relevance of chosen efficacy and harm outcomes is strongly recommended	6
Participant timeline	#13	Time schedule of enrolment, interventions (including any run-ins and washouts), assessments, and visits for participants. A schematic diagram is highly recommended (see Figure)	6
Sample size	#14	Estimated number of participants needed to achieve study objectives and how it was determined, including clinical and statistical assumptions supporting any sample size calculations	6
Recruitment	#15	Strategies for achieving adequate participant enrolment to reach target sample size	6
Allocation: sequence generation	#16a	Method of generating the allocation sequence (eg, computer-generated random numbers), and list of any	n/a

For peer review only

		factors for stratification. To reduce predictability of a random sequence, details of any planned restriction (eg, blocking) should be provided in a separate document that is unavailable to those who enrol participants or assign interventions	
Allocation concealment mechanism	#16b	Mechanism of implementing the allocation sequence (eg, central telephone; sequentially numbered, opaque, sealed envelopes), describing any steps to conceal the sequence until interventions are assigned	n/a
Allocation: implementation	#16c	Who will generate the allocation sequence, who will enrol participants, and who will assign participants to interventions	n/a
Blinding (masking)	#17a	Who will be blinded after assignment to interventions (eg, trial participants, care providers, outcome assessors, data analysts), and how	n/a
Blinding (masking): emergency unblinding	#17b	If blinded, circumstances under which unblinding is permissible, and procedure for revealing a participant's allocated intervention during the trial	n/a
Data collection plan	#18a	Plans for assessment and collection of outcome, baseline, and other trial data, including any related processes to promote data quality (eg, duplicate measurements, training of assessors) and a description of study instruments (eg, questionnaires, laboratory tests) along with their reliability and validity, if known. Reference to where data collection forms can be found, if not in the protocol	6
Data collection plan: retention	#18b	Plans to promote participant retention and complete follow-up, including list of any outcome data to be collected for participants who discontinue or deviate from intervention protocols	6
Data management	#19	Plans for data entry, coding, security, and storage, including any related processes to promote data quality (eg, double data entry; range checks for data values).	7

For peer review only

		Reference to where details of data management procedures can be found, if not in the protocol	
Statistics: outcomes	#20a	Statistical methods for analysing primary and secondary outcomes. Reference to where other details of the statistical analysis plan can be found, if not in the protocol	9
Statistics: additional analyses	#20b	Methods for any additional analyses (eg, subgroup and adjusted analyses)	9
Statistics: analysis population and missing data	#20c	Definition of analysis population relating to protocol non-adherence (eg, as randomised analysis), and any statistical methods to handle missing data (eg, multiple imputation)	9
Data monitoring: formal committee	#21a	Composition of data monitoring committee (DMC); summary of its role and reporting structure; statement of whether it is independent from the sponsor and competing interests; and reference to where further details about its charter can be found, if not in the protocol. Alternatively, an explanation of why a DMC is not needed	11
Data monitoring: interim analysis	#21b	Description of any interim analyses and stopping guidelines, including who will have access to these interim results and make the final decision to terminate the trial	11
Harms	#22	Plans for collecting, assessing, reporting, and managing solicited and spontaneously reported adverse events and other unintended effects of trial interventions or trial conduct	11
Auditing	#23	Frequency and procedures for auditing trial conduct, if any, and whether the process will be independent from investigators and the sponsor	11
Research ethics approval	#24	Plans for seeking research ethics committee / institutional review board (REC / IRB) approval	12

For peer review only

Protocol amendments	#25	Plans for communicating important protocol modifications (eg, changes to eligibility criteria, outcomes, analyses) to relevant parties (eg, investigators, REC / IRBs, trial participants, trial registries, journals, regulators)	12
Consent or assent	#26a	Who will obtain informed consent or assent from potential trial participants or authorised surrogates, and how (see Item 32)	12
Consent or assent: ancillary studies	#26b	Additional consent provisions for collection and use of participant data and biological specimens in ancillary studies, if applicable	12
Confidentiality	#27	How personal information about potential and enrolled participants will be collected, shared, and maintained in order to protect confidentiality before, during, and after the trial	12
Declaration of interests	#28	Financial and other competing interests for principal investigators for the overall trial and each study site	12
Data access	#29	Statement of who will have access to the final trial dataset, and disclosure of contractual agreements that limit such access for investigators	12
Ancillary and post trial care	#30	Provisions, if any, for ancillary and post-trial care, and for compensation to those who suffer harm from trial participation	12
Dissemination policy: trial results	#31a	Plans for investigators and sponsor to communicate trial results to participants, healthcare professionals, the public, and other relevant groups (eg, via publication, reporting in results databases, or other data sharing arrangements), including any publication restrictions	12
Dissemination policy: authorship	#31b	Authorship eligibility guidelines and any intended use of professional writers	12
Dissemination policy: reproducible research	#31c	Plans, if any, for granting public access to the full protocol, participant-level dataset, and statistical code	12

For peer review only

Informed consent materials	#32	Model consent form and other related documentation given to participants and authorised surrogates	Appendix 2
Biological specimens	#33	Plans for collection, laboratory evaluation, and storage of biological specimens for genetic or molecular analysis in the current trial and for future use in ancillary studies, if applicable	Appendix 2

The SPIRIT checklist is distributed under the terms of the Creative Commons Attribution License CC-BY-ND 3.0. This checklist was completed on 07. February 2018 using http://www.goodreports.org/, a tool made by the EQUATOR Network in collaboration with Penelope.ai

For peer review only

BMJ Open

Treatment Options in Idiopathic Subglottic Stenosis: Protocol for a Prospective International Multicenter Pragmatic Trial

Journal:	*BMJ Open*
Manuscript ID	bmjopen-2018-022243.R1
Article Type:	Protocol
Date Submitted by the Author:	28-Feb-2018
Complete List of Authors:	Gelbard, Alexander; Vanderbilt University, Otolaryngology<br>Shyr, Yu; Vanderbilt University,<br>Berry, Lynne; Vanderbilt University, Biostatistics<br>Hillel, Alexander ; Johns Hopkins University, Otolaryngology<br>Ekbom, Dale; Mayo Clinic Department of Otorhinolaryngology<br>Edell, Eric; Mayo Clinic Department of Pulmonology<br>Kasperbauer, Jan; Mayo Clinic Department of Otorhinolaryngology<br>Lott, David; Mayo Clinic Scottsdale, Otorhinolaryngology<br>Donovan, Donald; Baylor College of Medicine Department Otolaryngology<br>Garrett, C. Gaelyn; Vanderbilt University, Otolaryngology<br>Sandhu, Guri; Imperial College Healthcare NHS, Otolaryngology<br>Daniero, James; University of Virginia Health System, Otolaryngology<br>Netterville, James; Vanderbilt University, Otolaryngology<br>Schindler, Josh; Oregon Health and Science University, Otolaryngology<br>Smith, Marshall; University of Utah, Otolaryngology<br>Bryson, Paul; The Cleveland Clinic, Otolaryngology<br>Lorenz, Robert; The Cleveland Clinic, Otolaryngology<br>Francis, David; University of Wisconsin School of Medicine and Public Health, Surgery
**Primary Subject Heading**:	Ear, nose and throat/otolaryngology
Secondary Subject Heading:	Patient-centred medicine, Research methods, Respiratory medicine, Surgery
Keywords:	Pragmatic Trial, PR02, NoAAC, iSGS, Subglottic Stenosis

SCHOLARONE™
Manuscripts

Treatment Options in Idiopathic Subglottic Stenosis: Protocol for a Prospective International Multicenter Pragmatic Trial

Gelbard A[1], Shyr Y[2], Berry L[2], Hillel AT[3], Ekbom DC[4], Edell ES[4], Kasperbauer JL[4], Lott DG[5], Donovan DT[6], Garrett CG[1], Sandhu G[7], Daniero JJ[8], Netterville JL[1], Schindler JS[9], Smith ME[10], Bryson PC[11], Lorenz RR. [11], Francis DO[12].

Keywords: Pragmatic Trial, PR02, NoAAC, iSGS, Subglottic Stenosis

Word count: 6872

For peer review only

Corresponding Author:
Alexander Gelbard, M.D.
Associate Professor, Department of Otolaryngology, Vanderbilt School of Medicine
Medical Center East, S. Tower.
1215 21st Ave. South, Suite 7302, Nashville, TN 37232-8783.
(alexander.gelbard@vanderbilt.edu)
615.936.4737

Authors:

Alexander Gelbard	[1]Otolaryngology	Vanderbilt University Medical Center	Nashville, TN
Yu Shyr	[2]Biostatistics	Vanderbilt University Medical Center	Nashville, TN
Lynne Berry	[2]Biostatistics	Vanderbilt University Medical Center	Nashville, TN
Alexander Hillel	[3]Otolaryngology	The Johns Hopkins Hospital	Baltimore, MD
Dale Ekbom	[4]Otolaryngology	Mayo Clinic Rochester	Rochester, MN
Eric Edell	[4]Pulmonology	Mayo Clinic Rochester	Rochester, MN
Jan Kasperbauer	[4]Otolaryngology	Mayo Clinic Rochester	Rochester, MN
David Lott	[5]Otolaryngology	Mayo Clinic Scottsdale	Scottsdale, AZ
Donald Donovan	[6]Otolaryngology	Baylor College of Medicine	Houston, TX
Gaelyn Garrett	[1]Otolaryngology	Vanderbilt University Medical Center	Nashville, TN
Guri Sandhu	[7]Otolaryngology	Imperial College Healthcare NHS	London, England
James Daniero	[8]Otolaryngology	University of Virginia Health System	Charlottesville, VA
James Netterville	[1]Otolaryngology	Vanderbilt University Medical Center	Nashville, TN
Joshua Schindler	[9]Otolaryngology	Oregon Health & Science University	Portland, OR
Marshall Smith	[10]Otolaryngology	The University of Utah	Salt Lake City, UT
Paul Bryson	[11]Otolaryngology	Cleveland Clinic	Cleveland, OH
Robert Lorenz	[11]Otolaryngology	Cleveland Clinic	Cleveland, OH
David Francis	[12]Otolaryngology	University of Wisconsin	Madison, WI

For peer review only - http://bmjopen.bmj.com/site/about/guidelines.xhtml

Title

Treatment Options in Idiopathic Subglottic Stenosis: Protocol for a Prospective International Multicenter Pragmatic Trial

Abstract

Introduction. Idiopathic subglottic stenosis (iSGS) is an unexplained progressive obstruction of the upper airway that occurs almost exclusively in adult, Caucasian women. The disease is characterized by mucosal inflammation and localized fibrosis resulting in life-threatening blockage of the upper airway. Because of high recurrence rates, iSGS patients will frequently require multiple procedures following their initial diagnosis. Both the disease and its therapies profoundly affect patients' ability to breathe, communicate and swallow. A variety of treatments have been advanced to manage this condition. However, comparative data on effectiveness and side effects of the unique approaches have never been systematically evaluated. This study will create an international, multi-institutional prospective cohort of iSGS patients. It will compare 3 surgical approaches to determine how well the most commonly used treatments in iSGS "work"; and what quality of life trade-offs are associated with each approach.

Methods and analysis. A prospective pragmatic trial comparing the "Standard of Care" for iSGS at multiple international institutions. Patients with a diagnosis of iSGS without clinical or laboratory evidence of vasculitis, or a history of endotracheal intubation 2 years prior to symptom onset will be included in the study. Prospective evaluation of disease recurrence requiring operative intervention, validated patient reported outcome measures, as well as patient generated health data (mobile peak flow recordings and daily steps taken) will be longitudinally tracked for 36 months. The primary endpoint is treatment effectiveness defined as time to recurrent operative procedure. Secondary endpoints relate to treatment side effects and include patient reported outcome measures in voice, swallowing, breathing, and global quality of life, as well as patient generated health data.

Ethics and dissemination. This protocol was approved by the local IRB Committee of the Vanderbilt University Medical Center in July 2015. The findings of the trial will be disseminated through peer-reviewed journals, national and international conference presentations and directly to iSGS patient via social media-based support groups.

Registration details. http://www.clinicalstrials.gov identifier NCT02481817.

For peer review only

<u>Strengths of this study</u>

- Unique international prospective cohort of the rare disease Idiopathic Subglottic Stenosis (iSGS)
- Methodology evaluates 3 common surgical approaches in iSGS using objective clinical endpoints to enable assessment of their "real-world" effectiveness.
- Study design also integrates longitudinal assessment of validated patient reported outcome measures to compare patient-perceived functional outcomes of 3 common surgeries in iSGS.

<u>Limitations of this study</u>

- Nonrandomized design
- Adequate statistical power relies on sufficient recruitment which can be a challenge in rare disease.

Protocol Title: Treatment Alternatives in Adult Rare Disease; Assessment of Options in Idiopathic Subglottic Stenosis. North American Airway Collaborative PR-02 Study, A Prospective Pragmatic Trial.

Trial registration: http://www.clinicalstrials.gov identifier NCT02481817.
World Health Organization Trial Registration Data Set v1.3.

Protocol version: 05.23.2015, version 2.

Funding: This work was supported by the Patient Centered Outcomes Research Institute (PCORI): Grant ID: 1409-22214

Roles and responsibilities:
Principle Investigators AG & DOF were responsible for study conception and design, planning, conduct and acquisition of data, analysis and interpretation of data, and reporting of the results. AH, JD, DE & PB were responsible for study conception and design. YS, and LB were responsible for acquisition of data, analysis and interpretation of data. EE, JK, DD, RL, DL, GG, JN, JS, GS & MS participated in study conception and design, planning, and conduct and acquisition of data. Patient partner Catherine Anderson was responsible for study conception and design, planning, conduct and acquisition of data.

Patient Center Outcomes Research Institute (PCORI).www.pcori.com
Dr. Layla Lavasani Ph.D.
Senior Program Officer, Clinical Effectiveness and Decision Science
Patient-Centered Outcomes Research Institute
1919 M St. NW Washington, DC 20036
Office: 202-370-9408
llavasani@pcori.org

Study sponsor and funder had no role (and no ultimate authority) in study design; collection, management, analysis, and interpretation of data; writing of the report; the decision to submit the report for publication, including whether they will have ultimate authority over any of these activities

The composition, roles, and responsibilities of the coordinating center, steering committee, data management team can be found at (https://noaac.net/about/leadership-team/)

For peer review only

A. Introduction.

Background and rationale. Idiopathic subglottic stenosis (iSGS) is an unexplained progressive obstruction of the upper airway that occurs almost exclusively in adult, Caucasian women[1 2]. The disease is characterized by mucosal inflammation and localized fibrosis resulting in life-threatening blockage of the upper airway. Although uncommon (with an estimated incidence of 1:400,000 persons per year[3]), both the disease and its therapies profoundly affect patients' ability to breathe[4], communicate[5] and swallow[6]. Dyspnea is the hallmark symptom and the primary cause of death and disability[7]. However, patients can also experience debilitating voice changes[5 8 9] and swallowing problems[10] due to the condition (**Figures 1A, 1B, & 1C**) or its treatment.

People with this disease often require several surgeries per year[11]. A variety of treatments have been advanced to manage this condition[3 4 11] but are generally categorized into: 1) endoscopic dilation of the tracheal stenosis (accomplished with rigid instruments or inflatable balloons(**Figure 2A**). 2) endoscopic resection of the stenosis (with prolonged medical therapy after surgery: **Figure 2B**), or 3) open surgery with resection of the affected tracheal segment with end-to-end anastomosis (**Figure 2C**). Each patient can require repeated surgeries to keep their trachea open, which increases odds of treatment side effects and complications. All approaches have unique associated side effects, which can significantly affect the patient's quality of life. However, comparative data on trade-offs have never been systematically evaluated.

As a product of disease rarity, there is a lack of high-quality data to inform individual patient decision-making in iSGS. Limited evidence on treatment outcomes complicates patient decision-making as they try to balance survival, symptoms, and quality of life considerations. The proposed prospective study is designed to fill this void and leverages and expands upon a previous retrospective multi-institutional investigation, the North American Airway Collaborative RP-01 (NoAAC RP-01)[1]. In that study, nearly 500 iSGS patients from ten expert centers were retrospectively examined for the need for repeat surgery, and the time to recurrent disease (**Figure 3**). Interestingly, the demographics of this condition were quite similar at the separate centers. iSGS appears to nearly universally affect otherwise healthy, 40 – 60 year old Caucasian women (median age 50.3, 95% CI 49.1 – 51.5)[11] The NoAAC RP-01 study also found variation in the standard of care for iSGS at expert centers. Three basic treatment approaches were used (i.e. endoscopic dilation, endoscopic resection with adjuvant medical therapy, and open surgery) despite an absence of randomized controlled trials (RCTs) or other rigorous comparative studies to assess their differential effectiveness at avoiding disease recurrence. In contrast to the retrospective nature of RP-01, the prospective design of the NoAAC PR-02 study will enable rigorous treatment comparisons to determine how well the most commonly used surgeries in iSGS "work".

While evaluating treatment effectiveness is important, it is equally critical that the patient experience with the disease itself and its treatment be systematically characterized. This is imperative since patient and physician perspectives are often significantly discordant[12]. To this end, NoAAC PR-02 will collect patient-reported outcomes (PROs) in the cohort at initial presentation and at *a priori* determined intervals thereafter, (e.g. immediately pre-intervention, as well as 3, 6, and 12 months post-intervention) and conduct qualitative patient interviews (labeled "engagement studios") to better understand the health-related quality of life (HRQOL) and functional outcomes of therapy in iSGS. These endpoints are important to patients and are arguably a primary determinant in decision-making. For example, results show that endoscopic dilation is associated with a higher rate of disease recurrence and thus need for repeated surgery. Meanwhile, open cricotracheal resection is a major surgery with significant immediate perioperative risks and has been associated with alterations in voice[5 8] and swallowing[6]. Open surgery appears to reduce the risk of disease recurrence, but the degree of benefit, and the trade-offs associated with this invasive surgery are questions that demand prospective study.

Explanation for choice of comparators. The most common contemporary treatments for iSGS will be compared. These treatments (*described previously*) were identified during the preliminary RP-01 study. Patients with this condition have scarring with their trachea and present to the healthcare system with symptoms of airway obstruction (stridor) and respiratory distress. They universally require expeditious intervention to survive. Thus, use of a placebo or "no intervention" control is clinically unethical and inappropriate. The most widely employed treatment is endoscopic dilation of the tracheal scar (60% of patients in RP-01). Patients treated using this approach will be the primary comparator "control" group for this study. Outcomes will be compared to two other interventions: 1) endoscopic resection with adjuvant medical therapy and 2) tracheal resection which comprise approximately 20% of iSGS treatments, respectively.

For peer review only - http://bmjopen.bmj.com/site/about/guidelines.xhtml

Objectives. Specific hypotheses under investigation include:

1. There is variation in time to recurrence (TTR) between centers using similar surgical approaches for iSGS

2. There is variation in TTR between centers using different surgical approaches for iSGS.

3. The different surgical approaches are associated with unique tradeoffs in terms of voice, swallowing, breathing, and global quality of life.

Trial design. Prospective Pragmatic Trial investigating treatment effectiveness in the care of idiopathic subglottic stenosis patients

B. Methods: Participants, interventions, and outcomes

Study setting: International academic medical centers. North American Airway Collaborative Participating Sites can be found at https://noaac.net/about/noaac-sites/. All are academic centers that are tertiary referral centers for iSGS and thus have significant experience treating this rare condition[3 11 13-16]. Nearly all iSGS patients are ultimately referred for care and cluster at such high-volume centers, and thus we anticipate enrolling a representative patient cohort. Each research site has appropriate technological infrastructure for data collection and pathologic specimen tracking. The trial will be registered at ClinicalTrials.gov utilizing the World Health Organization Trial Registration Data Set v1.3 (**Appendix 1).**

Eligibility criteria:

Inclusion Criteria:
- >18 years of age.
- The obstructive airway lesion must involve the subglottis.

Exclusion Criteria
- Patients without capacity to consent for themselves
- History of endotracheal intubation or tracheotomy within 2 years of first symptoms.
- History of significant laryngotracheal traumatic injury.
- History of major anterior neck surgery.
- History of neck irradiation.
- History of caustic or thermal injury to the laryngotracheal complex.
- History of a clinically diagnosed vasculitis or collagen vascular disease.
- Positive antinuclear cytoplasmic antibody (ANCA) titers.

Interventions. Once consented (**Appendix 2**) and enrolled, patients will receive standard of care treatment at the respective center (**Appendix 3**). Employing our established digital trial infrastructure patients will be followed longitudinally for symptom changes, need for further treatment, complications, and will have PROs administered at *a priori* determined intervals.

Three interventions being compared include: 1) endoscopic dilation, 2) endoscopic resection of the airway narrowing with subsequent long-term medical therapy, and 3) open neck surgery (**Figure 2**). In endoscopic dilation, the patient undergoes transoral exposure of the tracheal scar with dilation of the scar by either rigid instrument, or controlled radial expansion device (CRE, i.e. balloon dilation). Somewhat similarly, in endoscopic resection the patient undergoes transoral exposure of the tracheal scar, however a CO_2 laser is then utilized to resect a significant portion of the scar, followed by long-term adjuvant medical therapy (Anti-reflux, antibacterial, and inhaled corticosteroid). In open neck surgery (a.k.a tracheal or cricotracheal resection), the trachea is approached via an external incision, the scarred segment of trachea is resected, and the remaining ends sewn back together.

There is significant homogeneity in treatment approach at each participating study. This has been confirmed both through our preliminary RP-01 data and direct correspondence with treating surgeons at these

centers. In fact, study sites use a singular surgical approach to treat 95% of their iSGS patients[3]. Personal communications with treating surgeons further indicate that the rationale for consistency in approach is largely experiential. To monitor and ensure consistency, each study site principal co-investigator will submit a formalized therapeutic protocol delineating their institutional "standard of care" for iSGS patients to ensure treatment and data homogeneity (**Appendix 3**). Centers are expected adhere to their internal "standard of care" in the management of the iSGS patients. After surgical treatment, national KSP will review the operative report for deviation from the formalized therapeutic protocol (describing how and why the procedure differed from local standard) and enter the results into the digital trial interface.

Outcomes. Interviews with both participating providers and iSGS patients were performed to reach consensus on the primary clinical outcomes to be compared across treatment modalities. Both groups were urged to select outcomes based on what data would be most critical and useful to patients and providers in decision-making. Clinical treatment outcomes identified were: 1) time to recurrence (TTR) or need for repeat procedure to open up their airway obstruction. The specific primary outcome to be measured is time to second treatment (2nd– 1st treatment date). This is an appropriate surrogate measure for recurrence since patient-reported symptoms of respiratory distress are the universal trigger for worsening symptoms, which is nearly perfectly correlated with additional treatment at these study sites. Measuring this outcome is feasible within the 3-year timeframe of the proposed. In our preliminary RP-01 study, recurrence occurred a median 8 months (95% CI 7.2-11.3 months) following initial treatment. It was experienced by 80% of patients. The time to recurrence varied both between iSGS patients receiving the same treatment, and also between the three treatments being prospectively compared in the proposed study (**Figure 3**).

Participant timeline: NoAAC PR02 time schedule of enrolment, interventions, and assessments, and visits for participants. (Schematic diagram: **Figure 4**)

Sample size. The primary endpoint of this trial is time to recurrence. The sample size estimation is completed using the 95% confidence interval (CI) method. With the proposed sample size of 300 (endoscopic therapy ≈ 180, open surgery ≈ 60 and endoscopic resection ≈ 60), the half-width of the 95% confidence interval for the time to recurrence function will be less than .25 for endoscopic resection and open surgery groups, and will be less than .15 for the endoscopic dilation group.

Recruitment. Study participants will be recruited via several mechanisms. Direct recruitment by participating clinician providers that diagnose and treat these patients will occur at each of the participating centers. It is expected that the majority of patients will be recruited through this approach. Direct recruitment will also be accomplished via partnered social media based advocacy groups in Facebook® and Yahoo®. Interested stakeholders can also identify this study through its registration with ClinicalTrials.gov, and the National Organization for Rare Disorders (NORD) rare disease database. Once consented and enrolled, patients will undergo standard of care treatment at the respective center and will be followed longitudinally for symptom changes, need for further treatment, complications, and will have PROs administered at *a priori* determined intervals. Given the overall enthusiasm within the iSGS community for our study, and the lack of any other clinical studies in iSGS (indicating an absence of study fatigue), we anticipated a 90-95% response rate for all interval data collections.

C. Methods: Data collection, management, and analysis

Data collection methods. Data collection for the proposed clinical cohort study will include the following case report forms (CRFs), implemented in an electronic data capture (EDC) system. EDC development and implementation details below (**Figure 5**).

- Baseline. At initial patient presentation, baseline data collected will include demographics, medical and surgical history, physical exam findings, and relevant diagnostics (e.g., CT scan findings, GI study values, laboratory values). Relevant to this study and this disease process will be reproductive, rheumatologic, and immunologic history. These data will involve detailed medical record abstraction, which will be performed by the national nurse coordinator. Quality and accuracy will be ensured double-entering 5% of charts and reviewing results with the lead investigators to ensure data robustness.
- Procedure. Details of most recent surgical procedure (i.e. *Index procedure*) will be captured in brief; data elements will include date of procedure (which can predate trial enrollment), institution at which procedure was performed, operative findings (e.g. site and degree of narrowing within the trachea), degree of and indicator of any deviations from local site protocol (i.e. local standard treatment approach).

- Recurrence. At patient recurrence (identified directly by patients on recurring scheduled automated follow-up questionnaire, or by communication from local site investigators), a minimum subset of features captured at baseline will be captured again; in addition, the details of the repeat procedure(s) will be abstracted from local operative report into the EDC and captured as for the initial procedure.
- Patient-reported outcomes (PROs). Four “traditional” validated PRO instruments will be utilized to assess patient quality of life. These relate to voice (VHI-10), dysphagia (EAT-10), breathing (COPD dyspnea), and general quality of life (SF-12). Additionally 4 “Non-traditional” PROs focused on 1) social support[17], participatory decision-making style[18], disease anxiety and burden[19] and fear of disease recurrence[20] will also be administered at the initial visit. Patients will be asked to complete “traditional” and “non-traditional PROs at baseline. The “traditional” PROs will be repeated at routine intervals post-procedure (e.g., at 6, 12, 18, 24 & 36 months). For patients who confirm reliable Internet access and email connectivity, interval PRO completion will be done directly by the patient into the web-based data capture instrument, with automated email reminders to patients at each PRO interval. For patients without Internet access, completion of PROs will be via mailed paper forms, or over the phone with a nurse coordinator; the nurse coordinator then will transfer PRO data from paper to the EDC. In all cases, patient failure to complete a required PRO in a timely manner will prompt an automated email to the nurse coordinator for an escalation of follow-up to minimize missing data.

Harmonization of datasets and use of CDEs: *All data will be collected prospectively; therefore, harmonization of existing datasets will not be necessary. Instead, common data elements (CDEs) to be collected across participating sites will be developed; the following items of documentation will be developed to ensure clarity of CDEs and comparable data collection across sites.*

- Workflow document. The workflow document provides a graphical representation of the timeline and logic of data collection, as well as data relationships to be modeled in the electronic data capture system (Figure 5).
- Data dictionary. The data dictionary describes the database structure, and details each data element, including variable name, label, graphical-user interface (GUI) format, value domain, definition, and any custom notes for implementation.
- GUI mock-ups. Prior to initiating development of the web-based data capture application; mock-ups of the graphical user interface are developed for review with system end-users and other appropriate stakeholders. Although the GUI layout of the EDC may be inferred from the data dictionary, the visual mock-up can provide a more accessible point of review for users, and allow for fine-tuning of project features.
- User manual. A detailed user manual will be developed for the data capture application. The manual will include step-by-step screenshots to guide users through data entry procedures, with highlighted tips for most efficient use of the system.
- Video training. A screencast also will be developed, as a live demonstration of data entry procedures.
- Auxiliary documentation. Auxiliary documentation includes description of user roles/permissions, detailed descriptions of unusually complex functionalities, or similar custom documentation. In addition, all system testing and debugging activity is documented on password-protected electronic boards accessible only to our development team.

Data entry, collection, and quality control

Data collection and management for the proposed project will be performed via a custom web-based electronic data capture system (EDC), implementing each of the CRFs described above in electronic format (eCRFs), and built using the Ruby on Rails (RoR) platform. RoR is an open-source web application development framework for creating rich Internet applications that model complex data and reinforce data integrity through custom validations. The RoR framework for web-based data entry will be linked to a MySQL database for the data storage component. All data entry interfaces will utilize dropdown menus, radio buttons/checkboxes, and other structured variable formats whenever feasible, to enforce consistency of variable values in data entry. Also, to support data quality control, extensive validations will be developed; these validations check records for internal consistency, conformity to any pre-specified data ranges, as well as compliance with any known inter-variable relationships. Support for longitudinal data will be provided through the use of a relational database architecture that models one-to-many relationships.

In addition to the use of structured variables and validations to prevent data entry errors, other mechanisms for data quality control include:

- Testing. The EDC will be tested extensively in both the development/staging and production environments. Automated testing is performed via scripted tests, to verify that observed behavior of the system conforms to expected behavior. Manual testing of all system features will also be employed via the user interface and performed by dedicated testing personnel in order to identify 1) bugs, 2) sub-optimal feature performance,

For peer review only - http://bmjopen.bmj.com/site/about/guidelines.xhtml

and 3) other issues that may negatively impact the user experience and/or integrity of the data collected. Testing results are documented via a standard operating procedure, in which bugs or other comments are posted to a web-based electronic board; each bug is then tracked on this board through the process of debugging, re-testing in the staging environment, deployment to production, and final testing in the production environment; thus, a permanent audit trail of testing is maintained. Additional user-facing testing is accomplished through ad hoc testing sessions, in which a group of volunteers follow a 'hack-a-thon' model in a 'mini-test-a-thon' session, with the intent to identify any remaining system or data entry loopholes that may allow for entry of nonsensical data or loss of data integrity. All testing results are documented and followed through to resolution of the issue.

- Access controls. Access to the EDC will be restricted to authorized personnel. Data entry personnel and other users are provided secure access to the web-based application via standard Internet technologies (i.e., HTTPS), with tiered access permissions appropriate to their study role; such access is granted only when the following criteria are met: 1) request for access is authorized by a study principal investigator or other designated key study personnel and is accompanied by a designation of the user's role, to ensure appropriate level of access; and 2) the user completes training on system use, which may include a live training provided by our group and/or documented completion of a pre-recorded video training. This training helps ensure that all users understand the system and the data elements, to minimize potential for data entry errors. All requests for user access, with documentation of fulfillment of the above criteria, are recorded in the permanent system documentation.
- User-facing validation feature. In addition to automated data validations run upon submission of data, a user-facing validation feature may be implemented to allow for electronic capture of the completion of manual data review for data monitoring purposes. This feature provides a 'Validate' button for each patient record, on a per-form basis; a designated user with appropriate role permissions uses this button to indicate that a form has passed manual review of data entry; the form is then date/time-stamped with the date and time of validation review. If a form is edited post-validation, the time-stamp of previous validation is retained, but the record returns to an un-validated state. An accompanying validation report may be generated from the system user interface, and provides for rapid identification of which forms have completed validation, and which require validation or re-validation; links within the reports take the user directly to the annotated forms.
- 21 CFR part 11 compliance. Systems designed for primary data capture of clinical trial data are 21 CFR part 11 compliant; all members of the database development team have completed training on the requirements of 21 CFR part 11 for electronic data capture systems.
- Review of datasets. Datasets extracted from our data capture systems are subject to a final review at time of extraction for interim review or other analysis, with evaluation of data distributions, any outlier or unexpected values, or other indicators that may suggest a need to review specific data points with the submitting user or institution to confirm data accuracy.

Additional key features of the EDC will include:

- Sophisticated query interface for extraction of data of interest for review or analysis. Our query interface uses a two-part approach that allows authorized users to (1) set filters for extraction of cases that meet criteria of interest and (2) select the data elements to be extracted for all cases meeting the filter criteria. For setting filters, all user-facing database variables populate a dropdown menu for selection of the variable to be filtered once a variable is selected, the value domain for that variable populates a second dropdown menu; these two dropdown menus are linked by selection of the operator of interest (i.e., 'equals', 'does not equal', 'equals any', 'contains', 'does not contain', etc.). Any number of variables may be set as filters, and the search may be set to return the union or intersection set of cases meeting the various criteria. In part 2 of the search interface, the user is presented with a list of all user-facing database variables, and selects, via checkbox, all variables of interest. Search results are returned in a downloadable Excel workbook with each relevant database table represented in a separate worksheet, including foreign table keys as needed to link records across tables. This format preserves the relation among tables, while avoiding creation of a single unwieldy table complicated by potential multiplicity of 1-to-n relationships.
- "Canned" reports. Canned reports for frequently requested summary statistics offer a useful adjunct to the query interface. Canned reports pull data from across database tables and assemble the data into a single unified report, according to the pre-specifications for the report. The reporting features calls an R-Sweave[21] script to assemble the data and generate the summary statistics, then write and output the report to PDF.

Patient Generated Health Data.

One important element of research is an understanding the experiences of individual patients, and one of the ways to learn about those experiences is by collecting patient-generated data. Such information can be obtained in a variety of ways, including during medical visits, through use of smartphones and other electronic devices, and as part of research studies. These techniques will be employed during our study to capture

For peer review only - http://bmjopen.bmj.com/site/about/guidelines.xhtml

patient activity (daily steps) and self-reported peak-flow measurements (**Figure 6A**). An established REDCap-based software platform (**Figure 6B**) will be used to allow patients to input hand-held peak flow data (**Figure 6C**) and provide the ability to track these measurements longitudinally over time.

The REDCap servers are housed in a local data center at Vanderbilt, and all web-based information transmission is encrypted. REDCap was developed specifically around HIPAA-Security guidelines and is recommended to Vanderbilt researchers by both our Privacy Office and Institutional Review Board. REDCap has been disseminated for local use at more than 940 other academic/non-profit consortium partners in 75 countries. Vanderbilt leads the REDCap Consortium, which currently supports more than 99,000 projects and 128,000 users. More information about the consortium and system security can be found at http://www.projectredcap.org/.

Statistical methods

The primary endpoint: The primary objective of this study is to evaluate the time to recurrence (TTR) among three possible treatment groups, i.e., endoscopic dilation, endoscopic resection, and open surgery.

Data analysis plan for the primary endpoint: Demographic information will be tabulated. Descriptive statistics, including means, standard deviations, and ranges for continuous parameters, as well as percent's and frequencies for categorical parameters, will be presented. For the primary objective analysis, i.e., estimating the TTR with 95% confidence interval, the TTR time will be estimated using the Kaplan-Meier method with the 95% confidence interval (CI). The Rothman CI, CI's based on Greenwoods variance, Thomas and Grunkemeier CI and the simultaneous confidence bands by Nair and Hall and Wellner will be reported. The logrank test will be used to compare the equality of survival curves. The generalized Wilcoxon and log-likelihood tests will also be examined, as these tests weight the survival function differently from the logrank test, which gives more weight to later occurring events. The Cox proportional hazards model will be applied to investigate potential prognostic factors, such as age on the TTR data. The adjusted p-values as well as the adjusted 95% confidence intervals from the Cox model will be reported. The adjusted hazard ratios and 95% CIs will be reported.

Secondary endpoints: differential treatment quality of life trade-offs will be prospectively and systematically assessed using both validated "traditional" and "non-traditional" patient reported outcome measures (PRO). 4 "Traditional" PRO will measure disability related to voice (VHI-10[22]), swallowing (EAT-10[23]), breathing (COPD dyspnea[24]), and global quality of life (SF-12[25]) (IR4). 4 "Non-traditional" PROs focused on 1) social support[17], participatory decision-making style[18], disease anxiety and burden[19] and fear of disease recurrence[20] will also be administered at the initial visit. Responses to PROs tend to change in chronic disease states since severity of the measured concept is time-variable. This is particularly true for iSGS patients whose symptoms markedly improve after treatment and revert and worsen before subsequent treatments. To better understand the breadth of patient experience with this condition, PROs will be employed at a priori determined intervals after diagnosis and treatment, (e.g. immediately pre-intervention, 3, 6, and 12, post-intervention), to obtain a more accurate portrait of the survivorship experience related to the different therapeutic modalities.

Data analysis plan for secondary endpoints: The secondary objective of this study is to evaluate the quality of life (QOL) scores, e.g., SF12, Dyspnea index, EAT-10, and VHI10, among three study groups. The 95% confidence interval (CI) method based on the Normal distribution will be applied to estimate the quality of life score among three study groups. The mixed effect model will be applied to exam the correlation between the quality of life score and the study groups.

The strategy to be used for developing multivariable models for QOL involves the following steps: (1) Apply multiple imputation for missing covariate values to make good use of partial information. (2) Choose an appropriate statistical model based on the nature of the response variable. (3) Decide on the allowable complexity of the model (i.e., the number of covariates) based on the effective sample size available. (4) Allow for nonlinear predictor effects using regression splines. (5) Incorporate pre-specified interactions. (6) Check distributional assumptions. (7) Adjust the variance-covariance matrix for multiple imputation. (8) Quantify the clinical utility (discrimination ability) of the model. (9) Internally validate the calibration and discrimination of the model using the bootstrap approach, e.g., .632+ bootstrap, to estimate the model's likely performance on a new set of subjects. The statistical analyses will be completed by either R 3.1.1 or SAS 9.4 statistical program in this project

For peer review only

Statistical strategy for addressing missing data: Given the enthusiasm of the iSGS population for this trial, we estimate a 90-95% response rate for the patient reported outcome measures in the cohort. Acknowledging this, it is possible that bias could be introduced due to missing data. To account for this, we will use two approaches in cases where participants are alive but are missing PRO data: the multiple imputation model based on the Markov chain Monte Carlo method described above[26] and a hierarchical hot-deck imputation approach[27 28]. We will perform sensitivity analyses compare the two methods to assess the validity of the two approaches. It is necessary to assume data are missing at random (MAR) to perform these types of pattern mixture imputation analyses[28]. In the hierarchical hot-deck imputation approach, participants with missing PRO data will be matched with at least 10 other participants with full data at the previous time point on the following variables: to voice (VHI-10), swallowing (EAT-10), breathing (COPD dyspnea), and general quality of life (SF-12), age, race, co-morbid disease, education and income. The order of matching will be determined by the dependent variable being examined. For the purposes of statistical testing, 10 complete data sets will be formed employing the hot-deck imputation approach[28]. Appropriate survey data analysis techniques will be performed using each data set, leading to 10 separate estimates of parameters and their covariances. We will use the mianalyze.relimp function in R to combine the parameter estimates and obtain covariance estimates which account for both within- and between-imputation sources of variation (*MD-5*). Inferences will be based on the combined parameter estimates and appropriate covariance structure. Our group has used this successfully in prior studies[29 30].

Statistical strategy to address heterogeneity of treatment effect: Subgroup analysis is the most commonly used analytic approach for examining HTE[31], and we will utilize an exploratory variant of this analysis in our approach. Definition of subgroups, endpoints, hypotheses, and modeling parameters will be derived in response to the data. An example of this would be the use of a backward model selection approach to identify treatment by covariate interactions. Some of the important types of subgroup variables will be: (1) demographic variables (e.g., age); (2) pathophysiologic variables (e.g., timing after recurrence, disease grade); (3) comorbidities (e.g., presence of diabetes); and (4) concomitant exposures (e.g., hormone replacement therapy, proton pump inhibitors). Additionally, "non-traditional" characteristics that affect patient decision-making (e.g. social support, patient decision-making style, disease related anxiety, baseline quality of life) will be collected to improve risk-adjustment and increase the individualization of the results. Although it is extremely difficult to obtain the sampling properties of subgroup effect estimators (e.g., standard errors), post hoc exploratory subgroup analyses may identify promising hypotheses that will be subject to more rigorous future examination.

Statistical strategy to address confounding by selection bias: Observational studies (like our proposed investigation) that lack randomization of subjects into treatment groups and must address selection bias to properly estimate the effect of treatment. We will apply a propensity scoring method (PSM) and instrumental variable (IV) method to adjust for observed and unobserved confounding, respectively. Propensity scoring will be used to mitigate the expected biases from observed confounding in the proposed observational study. It is a balancing score that effectively makes the distribution of measured baseline covariates similar between treatment groups. This is important because the apparent difference in outcome between those treated with endoscopic dilation versus those treated with endoscopic resection or open anterior neck surgery may depend on characteristics that affected whether or not a patient received a given treatment instead of due to the actual effect of the treatment. This issue is relevant to our study Aims #2 and #3, but for illustrative purposes, the specific analytic approach will be described in the context of Aim #2. Its objective is to determine factors that affect time to stenosis recurrence in patients with idiopathic subglottic stenosis. In this analysis, the dependent variable is time to recurrence and the primary independent variable is treatment type: endoscopic dilation versus endoscopic excision or open anterior neck surgery of the affected portion of the trachea.

There are three basic techniques for propensity score method: matching, stratification, and regression[32]. We plan to use two-step process of stratification and regression. Stratification consists of grouping subjects into strata determined by observed background characteristics; then comparing subjects between treatment groups directly. Propensity scoring on strata is particularly useful when there are large numbers of covariates, as is the case in this study. Conventionally, creation of five strata has been shown to remove 90% of bias due to the stratified variable[33 34]. The propensity score is then estimated using a logistic regression model, in which the treatment status is regressed on observed and stratified baseline characteristics. This allows for the formation of matched sets of patients who underwent endoscopic dilation, endoscopic resection or open anterior neck surgery for their iSGS based on similar propensity score values[35]. In essence, all collected and known confounding covariates will be collectively replaced by a single function of these covariates – the propensity score. Thus, the collection of known predictors is collapsed into a single predictor. Since time to recurrence is a continuous variable, the effect of treatment will be estimated as the difference between the mean time for patients in the endoscopic dilation versus endoscopic resection or open anterior neck surgery groups. Once treatment effect has been estimated using propensity scoring, variance of

outcome effect and statistical significance can be estimated. Analysis of the propensity matched treatment groups can be accomplished by directly comparing outcomes between the three treatment groups. Multivariate regression will be performed to reduce bias due to residual differences in observed baseline covariates between groups.

All known and measurable covariates and confounders will be collected and considered in the propensity score model. Incorporation to the multivariate model will be determined *a priori* based on their potential to confound or modify the association between treatment and time to recurrence, and include **demographics (**age, sex, race, socioeconomic status, geographic location, marital status), **health (**Charlson-Deyo score, body mass index), **endocrinologic history (**age of first menses, number of pregnancies, onset of menopause, use of hormone replacement therapy), **Inflammatory biomarkers** (high sensitivity CRP, mucosal atopic index), **anatomic** characteristics (degree of luminal obstruction), **physiologic** (peak flow rate), **social/behavioral** [Social Support (FSSQ), Quality of life (SF12), Decision-making style (PDMstyle), Fear of Progression (FoP-Q-SF)]. Provider-specific covariates will include the type of subspecialty training program, training location, procedural volume, and treatment criteria for open anterior neck surgery. In addition, the model will include interaction terms for the associations of age with endocrinologic history, Charlson-Deyo score, and facility regions based on statistical evidence of effect modification and theoretical plausibility.

Propensity scoring can only adjust for observed confounding variables such as those listed above not unobserved ones. Therefore, we will employ quantitative and limited variable (QLIM) analysis to adjust for unobserved confounding. The instrumental variable has to meet five specific assumptions: 1) potential outcomes for each patient are unrelated to treatment status of other patients, 2) instrument affects receipt of the treatment of interest, 3) this effect is always in the same direction, 4) instrument assigns treatment randomly, and 5) instrument has an effect on the outcome only through the treatment assignment[36] . The instrument that we plan to use in this analysis is distance from the patient's residence to the treating facility[37 38]. The assumption is that the association between distance to the hospital and time to recurrence is due only to the effect of relative distance on treatment assignment after controlling for observed variables and not directly correlated with time to recurrence. This process will involve a two-stage process that first uses the instrument variable and other covariates to predict the treatment. A second stage estimates the outcome by the predicted treatment (from the first model) and other covariates[39]. Using a two-stage approach has the advantage of incorporating the predicted treatment into the outcome model as it represents the portion of treatment selection related to distance from the treating facility. We will also adjust, when possible, for any instrument-outcome confounding, as confounding can still occur even with the instrumental variable procedure[36]. Other instruments will be considered if distance to treating facility is not found to meet the aforementioned assumptions or is found to significantly confound the outcome.

Patient and Public Involvement.

- *Development of the research question and outcome measures:* This patient-motivated and centered investigation involves systematic iSGS patient and clinician stakeholder engagement, which is required to comprehensively assess iSGS treatment options and their health and quality of life trade-offs. We have engaged participants with a lived understanding of the disease to help define its greatest impact on the patient health and quality of life. Perspective of patients is often quite discrete from that presumed by their clinicians. Patient and clinician stakeholders have been equally represented in formulating research questions, determining important characteristics affecting patient decisions, defining outcomes, identifying treatments and their potential side-effects and trade-offs. A critical patient partner and co-investigator on this proposal, Catherine Anderson, has performed groundbreaking work to engage the iSGS community. She founded the a social-media based iSGS support group, and has used that group as a platform to help understand patient needs, and the patient perspective on therapy and subsequent side effects. Her community of iSGS patients has played an integral role in identifying outcomes that matter to patients: survival, tracheostomy, voice, swallowing and breathing function, and health-related quality of life.

- *Study Design:* All member institutions and their associated patient partners have been equal partners in planning the proposed work. Dr. Gelbard (PI) from Vanderbilt University (the coordinating institution) has been in frequent personal correspondence with each group at meetings in May and September of 2014. These discussions informed study design, logistics, and eligibility requirements. Conversations with patient partner and co-investigator Catherine Anderson have been an integral part of trial design. A formal trial-planning meeting was conducted in London England in August 2014 with Catherine in attendance. She re-enforced the need for patient reported outcome measures in order to more precisely understand the trade-offs inherent in each therapy (endoscopic dilation, endoscopic resection, and open anterior neck surgery).

- *Recruitment:* NoAAC Steering Committee member and co-investigator Catherin Anderson (herself an iSGS patient) founded and directs the largest social media based iSGS support group. This resource will

For peer review only - http://bmjopen.bmj.com/site/about/guidelines.xhtml

provide rapid and inexpensively access to a large audience of patients with potential interest in trial participation, and will be utilized for trial recruitment.

- *Conducting the Study:* Several formal and informal channels will to be available to patients who wish to provide input to move to the highest level of trial management. This ensures patient engagement with the protocol to optimize patient experience and information gleaned. Specifically, regional patient advocates will report directly to NoAAC steering committee members and through direct feedback via the engaged social media support group (via co–investigator and support group director Catherine Anderson). Furthermore, open communication in expected and encouraged between patient partners and each study site clinician co-investigators to enable clear and consistent mechanism for patient participation and feedback regarding the process.

- *Disseminating Study Results:* Approximately 2 months before the end of the study timeline, we will have a final team meeting with members the study team and any interested patient partners. The focus will be a discussion of overall study results and on additional strategies to disseminate study results to appropriate scientific, community, and stakeholder groups. Results will be published within academic journals and presented at national medical meetings to broaden the scope of dissemination to clinician providers that encounter iSGS. They will also be made publically available through open access publication choices, and through patient advocacy sources (e.g. Facebook). One of our NoAAC Steering Committee members (an iSGS patient and co-investigator, Catherin Anderson) founded and directs the largest social media based iSGS support group. This resource allows bi-directional communication, quickly and inexpensively to provide an open channel for patient partner input. We plan to use this resource (along with other complementary outlets) for effectively disseminating information about the intervention to the national audience of iSGS patients.

D. **Methods: Monitoring**

Data monitoring: This is a non-intervention study and there is no DSMB. The Principal Investigator is responsible for monitoring protocol conduct and reporting any adverse events related to study procedures. Given the observational nature of the trial, Adverse Events (AE) reporting will include the reporting of any unanticipated patient confidentiality or data security events if it they are deemed probably or definitely related to being in this study. Although AEs are not anticipated on this study, if they should occur, they will be reported to the participating sites IRB board as well as the Vanderbilt Institutional Review Board by the Principal Investigator.

Harms: Minimal risk is anticipated. There are no investigational treatments under study in this project. Study patients will be asked to answer questions on self-administered hard copy electronic, mailed, or telephone surveys, and self-reported health data (i.e. peak flow breathing measurements). Additionally, limited information from their routine medical care will be obtained. Patients will be informed that they can refuse to answer any of the questions. They will also be told that refusal to participate in the study will not in any way change or alter the care they will receive. Patients will be told that survey data are to be obtained for research purposes only and that no individual results will be reported. The data will be kept strictly confidential. No data of any sort will be released to anyone outside the study for any reason. Individual patients are never identified in publications.

Risks to the participants will be minimized by proper screening of potential candidates and strict adherence to confidentiality rules. In addition, access to the Electronic Data Capture (EDC) system will be strictly restricted to authorized personnel. Data entry personnel and other users are provided secure access to the web-based application via standard Internet technologies (i.e., HTTPS), with tiered access permissions appropriate to their study role; such access is granted only when the following criteria are met: 1) request for access is authorized by a study principal investigator or other designated key study personnel and is accompanied by a designation of the user's role, to ensure appropriate level of access; and 2) the user completes training on system use, which may include a live training provided by our group and/or documented completion of a pre-recorded video training. 3) The user has appropriate training for protection of human subjects. All requests for user access, with documentation of fulfillment of the above criteria, are recorded in the permanent system documentation.

Auditing: The principle Investigator will perform site visits at participating institution during the trial During these visit, he will interview local site KSP for trial conduct and progress. Additionally, bi-annual internal audits of data integrity and completeness will be performed and the data coordinating center. An interim analysis will be performed at 12 months to verify robust data collection

For peer review only - http://bmjopen.bmj.com/site/about/guidelines.xhtml

E. Ethics and dissemination

Research ethics approval: Central institutional review board (REC/IRB) approval will be obtained from the Vanderbilt University Medical Center.

Protocol amendments: We plan to communicate important protocol modifications (eg, changes to eligibility criteria, outcomes, or analyses) to all relevant parties directly (e.g., investigators, and trial participants).

Consent: Local site KSP or the National Nurse Coordinator will obtain informed consent from potential trial participants (**Appendix 2**). Additional consent provisions will be explicitly articulated for collection and use of participant data and biological specimens in ancillary studies.

Confidentiality: How personal information about potential and enrolled participants will be collected, shared, and maintained in order to protect confidentiality before, during, and after the trial

Declaration of interests: Neither the principal investigators, nor the individual site investigators have financial or other competing interests for the proposed trial.

Access to data: The NoAAC study team is committed to promoting the principles of transparency, replication and reproducibility in research. Study documentation (e.g., database design, programming code, and data definitions) would be shared with requesting researchers, and any requesting peer-reviewed journals after PI approval. We will also share all a complete, cleaned, de-identified copy of the final data set with the funding agency (PCORI), and create an access-controlled website for de-identified data abstraction. Access to this resource will require investigator IRB approval along with NoAAC steering committee project approval.

Ancillary and post-trial care: Given the observational nature of this prospective pragmatic trial, there are not provisions for ancillary and post-trial care.

Dissemination policy: Team composition was deliberately designed to include individual experts in affected scientific, community, and stakeholder groups. Results will be published within academic journals and presented at national medical meetings to broaden the scope of dissemination to clinician providers that encounter iSGS. They will also be made publically available through open access sources and direct delivery to patient advocacy sources (e.g. Facebook). As noted by the chief information officer of one of our partnering patient advocacy organizations (the National Organization for Rare Disease: NORD), their organization will help disseminate results through their website and rare disease database.

Authorship eligibility guidelines: The formal authorship guidelines of the North American Airway Collaborative (NoAAC) will be followed for this proposal. There is no planned use of professional writers.

F. Appendices

Appendix 1: World Health Organization Trial Registration Data Set

Appendix 2: Informed consent materials: Model consent form and other related documentation given to participants.

For peer review only - http://bmjopen.bmj.com/site/about/guidelines.xhtml

Appendix 3: Local Site Surgical Protocol: Model protocol form for local participating site surgeons.

Figure Legends:

Figure 1. Diagram of normal trachea (A.), Healthy trachea (B.), & iSGS patient with obstructive tracheal scar (C.).

Figure 2. Treatment approaches for iSGS: 1) endoscopic dilation of the tracheal stenosis (accomplished with rigid instruments or inflatable balloons: A); 2) endoscopic resection of the stenosis (with prolonged medical therapy after surgery: B); or 3) open surgery with resection of the affected tracheal segment with end-to-end anastomosis (C.).

Figure 3. Kaplan Meier Analysis of NoAAC RP01 study results. Percentage of iSGS patients avoiding disease recurrence, stratified by treatment type.

Figure 4. NoAAC PR02 time schedule of enrolment, interventions, and assessments, and visits for participants.

Figure 5. Schematic overview of NoAAC PR02 trial workflow. Data collection for the proposed clinical cohort study will include the case report forms (CRFs), implemented in an electronic data capture (EDC) system.

Figure 6. Handheld peak-flow meter (A.), along with mobile device software (B.), for tracking patient-generated health data (C.).

For peer review only

References:

1. Gelbard A, Donovan DT, Ongkasuwan J, et al. Disease homogeneity and treatment heterogeneity in idiopathic subglottic stenosis. *The Laryngoscope* 2016;126(6):1390-6. doi: 10.1002/lary.25708
2. Gelbard A, Francis DO, Sandulache VC, et al. Causes and consequences of adult laryngotracheal stenosis. *The Laryngoscope* 2015;125(5):1137-43. doi: 10.1002/lary.24956
3. Maldonado F, Loiselle A, Depew ZS, et al. Idiopathic subglottic stenosis: An evolving therapeutic algorithm. *The Laryngoscope* 2014;124(2):498-503. doi: 10.1002/lary.24287 [published Online First: 2013/07/03]
4. Ashiku SK, Kuzucu A, Grillo HC, et al. Idiopathic laryngotracheal stenosis: effective definitive treatment with laryngotracheal resection. *The Journal of thoracic and cardiovascular surgery* 2004;127(1):99-107. doi: 10.1016/j.jtcvs.2002.11.001
5. Bryans L, Palmer AD, Schindler JS, et al. Subjective and objective parameters of the adult female voice after cricotracheal resection and dilation. *The Annals of otology, rhinology, and laryngology* 2013;122(11):707-16. [published Online First: 2013/12/24]
6. Miller CK, Linck J, Willging JP. Duration and extent of dysphagia following pediatric airway reconstruction. *International journal of pediatric otorhinolaryngology* 2009;73(4):573-9. doi: 10.1016/j.ijporl.2008.12.024
7. Nouraei SA, Sandhu GS. Outcome of a multimodality approach to the management of idiopathic subglottic stenosis. *The Laryngoscope* 2013;123(10):2474-84. doi: 10.1002/lary.23949
8. Ettema SL, Tolejano CJ, Thielke RJ, et al. Perceptual voice analysis of patients with subglottic stenosis. *Otolaryngology--head and neck surgery : official journal of American Academy of Otolaryngology-Head and Neck Surgery* 2006;135(5):730-5. doi: 10.1016/j.otohns.2006.06.1249
9. Hatcher JL, Dao AM, Simpson CB. Voice Outcomes After Endoscopic Treatment of Laryngotracheal Stenosis. *The Annals of otology, rhinology, and laryngology* 2014 doi: 10.1177/0003489414551980
10. Miller CK, Kelchner LN, de Alarcon A, et al. Compensatory laryngeal function and airway protection in children following airway reconstruction. *The Annals of otology, rhinology, and laryngology* 2014;123(5):305-13. doi: 10.1177/0003489414525920
11. Gelbard A, Francis DO, Sandulache VC, et al. Causes and consequences of adult laryngotracheal stenosis. *The Laryngoscope* 2014 doi: 10.1002/lary.24956
12. Suarez-Almazor ME, Conner-Spady B, Kendall CJ, et al. Lack of congruence in the ratings of patients' health status by patients and their physicians. *Medical decision making : an international journal of the Society for Medical Decision Making* 2001;21(2):113-21.
13. Hseu AF, Benninger MS, Haffey TM, et al. Subglottic stenosis: a ten-year review of treatment outcomes. *The Laryngoscope* 2014;124(3):736-41. doi: 10.1002/lary.24410
14. Dedo HH, Catten MD. Idiopathic progressive subglottic stenosis: findings and treatment in 52 patients. *The Annals of otology, rhinology, and laryngology* 2001;110(4):305-11.
15. Smith ME, Elstad M. Mitomycin C and the endoscopic treatment of laryngotracheal stenosis: are two applications better than one? *The Laryngoscope* 2009;119(2):272-83. doi: 10.1002/lary.20056

For peer review only

16. Taylor SC, Clayburgh DR, Rosenbaum JT, et al. Clinical manifestations and treatment of idiopathic and Wegener granulomatosis-associated subglottic stenosis. *JAMA otolaryngology-- head & neck surgery* 2013;139(1):76-81. doi: 10.1001/jamaoto.2013.1135
17. Sherbourne CD, Stewart AL. The MOS social support survey. *Social science & medicine* 1991;32(6):705-14.
18. Kaplan SH, Greenfield S, Ware JE, Jr. Assessing the effects of physician-patient interactions on the outcomes of chronic disease. *Medical care* 1989;27(3 Suppl):S110-27.
19. Broadbent E, Petrie KJ, Main J, et al. The brief illness perception questionnaire. *Journal of psychosomatic research* 2006;60(6):631-7. doi: 10.1016/j.jpsychores.2005.10.020
20. Mehnert A, Herschbach P, Berg P, et al. [Fear of progression in breast cancer patients--validation of the short form of the Fear of Progression Questionnaire (FoP-Q-SF)]. *Zeitschrift fur Psychosomatische Medizin und Psychotherapie* 2006;52(3):274-88.
21. F. L. Sweave: Dynamic generation of statistical reports using literate data analysis. . In: Rönz WHaB, ed. Compstat 2002 - Proceedings in Computational Statistics, pages 575-580. Heidelberg: Physica Verlag 2002:pages 575-80.
22. Rosen CA, Lee AS, Osborne J, et al. Development and validation of the voice handicap index-10. *The Laryngoscope* 2004;114(9):1549-56. doi: 10.1097/00005537-200409000-00009
23. Belafsky PC, Mouadeb DA, Rees CJ, et al. Validity and reliability of the Eating Assessment Tool (EAT-10). *The Annals of otology, rhinology, and laryngology* 2008;117(12):919-24.
24. Nouraei SA, Randhawa PS, Koury EF, et al. Validation of the Clinical COPD Questionnaire as a psychophysical outcome measure in adult laryngotracheal stenosis. *Clinical otolaryngology : official journal of ENT-UK ; official journal of Netherlands Society for Oto-Rhino-Laryngology & Cervico-Facial Surgery* 2009;34(4):343-8. doi: 10.1111/j.1749-4486.2009.01969.x
25. Gandek B, Ware JE, Aaronson NK, et al. Cross-validation of item selection and scoring for the SF-12 Health Survey in nine countries: results from the IQOLA Project. International Quality of Life Assessment. *Journal of clinical epidemiology* 1998;51(11):1171-8.
26. FE H. Regression Modeling Strategies, with Applications to Linear Models, Survival Analysis and Logistic Regression. . New York: Springer 2001.
27. Little RJ, Wang Y. Pattern-mixture models for multivariate incomplete data with covariates. *Biometrics* 1996;52(1):98-111.
28. DB. R. Multiple imputation for nonresponse in surveys. . New York: John Wiley & Sons 1987.
29. Penson DF, McLerran D, Feng Z, et al. 5-year urinary and sexual outcomes after radical prostatectomy: results from the Prostate Cancer Outcomes Study. *The Journal of urology* 2008;179(5 Suppl):S40-4. doi: 10.1016/j.juro.2008.03.136
30. Penson DF, McLerran D, Feng Z, et al. 5-year urinary and sexual outcomes after radical prostatectomy: results from the prostate cancer outcomes study. *The Journal of urology* 2005;173(5):1701-5. doi: 10.1097/01.ju.0000154637.38262.3a
31. Dorresteijn JA, Visseren FL, Ridker PM, et al. Estimating treatment effects for individual patients based on the results of randomised clinical trials. *Bmj* 2011;343:d5888. doi: 10.1136/bmj.d5888
32. D'Agostino RB, Jr. Propensity score methods for bias reduction in the comparison of a treatment to a non-randomized control group. *Statistics in medicine* 1998;17(19):2265-81.
33. Rosenbaum PR, Rubin DB. Difficulties with regression analyses of age-adjusted rates. *Biometrics* 1984;40(2):437-43.
34. Cochran WG. The effectiveness of adjustment by subclassification in removing bias in observational studies. *Biometrics* 1968;24(2):295-313.
35. Rosenbaum PR, Rubin DB. The bias due to incomplete matching. *Biometrics* 1985;41(1):103-16.
36. Garabedian LF, Zaslavsky AM, Soumerai SB. Instrumental variable analyses for observational comparative effectiveness research: the paired availability design. *Annals of internal medicine* 2014;161(11):841. doi: 10.7326/L14-5029-2
37. Rassen JA, Brookhart MA, Glynn RJ, et al. Instrumental variables I: instrumental variables exploit natural variation in nonexperimental data to estimate causal relationships. *Journal of clinical epidemiology* 2009;62(12):1226-32. doi: 10.1016/j.jclinepi.2008.12.005

For peer review only

38. Rassen JA, Brookhart MA, Glynn RJ, et al. Instrumental variables II: instrumental variable application-in 25 variations, the physician prescribing preference generally was strong and reduced covariate imbalance. *Journal of clinical epidemiology* 2009;62(12):1233-41. doi: 10.1016/j.jclinepi.2008.12.006
39. Angrist J, G. Imbens, and D. Rubin. Identification of Causal Effects Using Instrumental Variables. *Journal of the American Statistical Association* 1996;91:444-55.

For peer review only

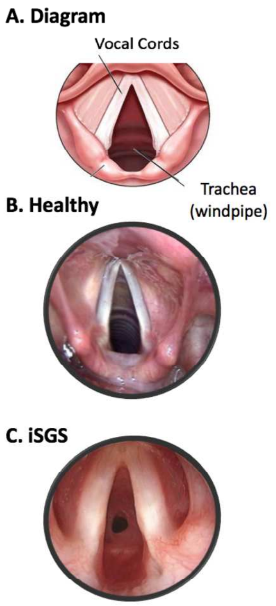

Figure 1. Diagram of normal trachea (A.), Healthy trachea (B.), & iSGS patient with obstructive tracheal scar (C.).

254x190mm (300 x 300 DPI)

w only

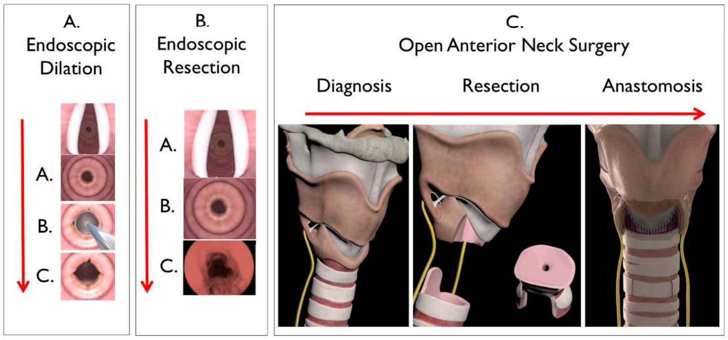

Figure 2. Treatment approaches for iSGS: 1) endoscopic dilation of the tracheal stenosis (accomplished with rigid instruments or inflatable balloons: A); 2) endoscopic resection of the stenosis (with prolonged medical therapy after surgery: B); or 3) open surgery with resection of the affected tracheal segment with end-to-end anastomosis (C.).

254x190mm (300 x 300 DPI)

only

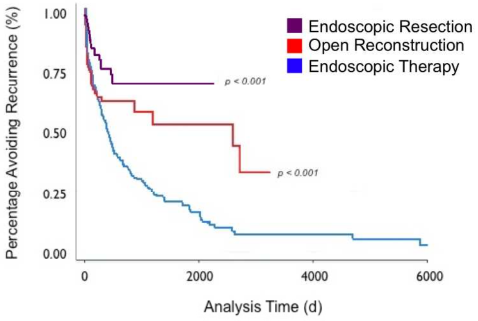

Figure 3. Kaplan Meier Analysis of NoAAC RP01 study results. Percentage of iSGS patients avoiding disease recurrence, stratified by treatment type.

254x190mm (300 x 300 DPI)

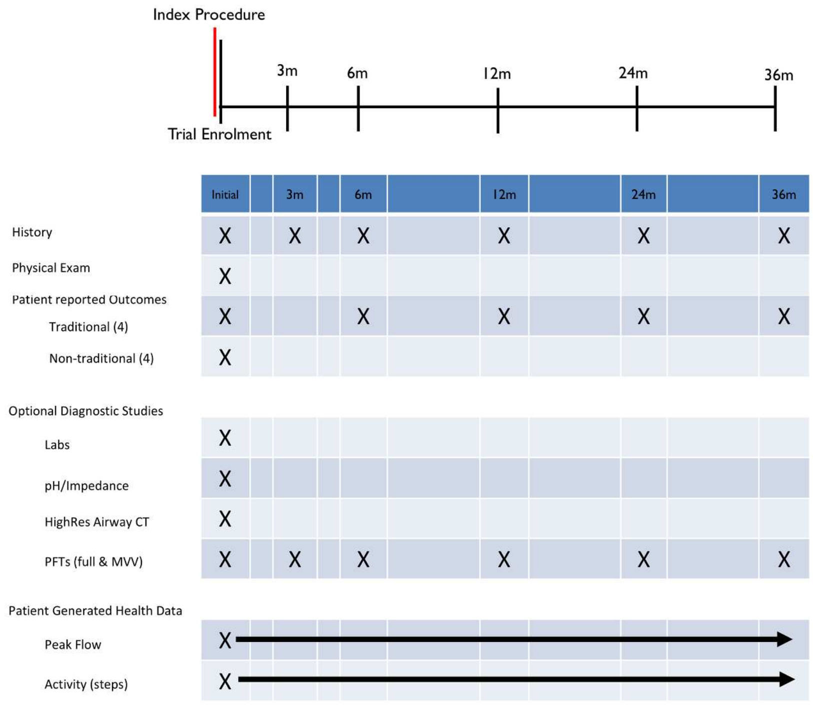

Figure 4. NoAAC PR02 time schedule of enrolment, interventions, and assessments, and visits for participants.

254x190mm (300 x 300 DPI)

w only

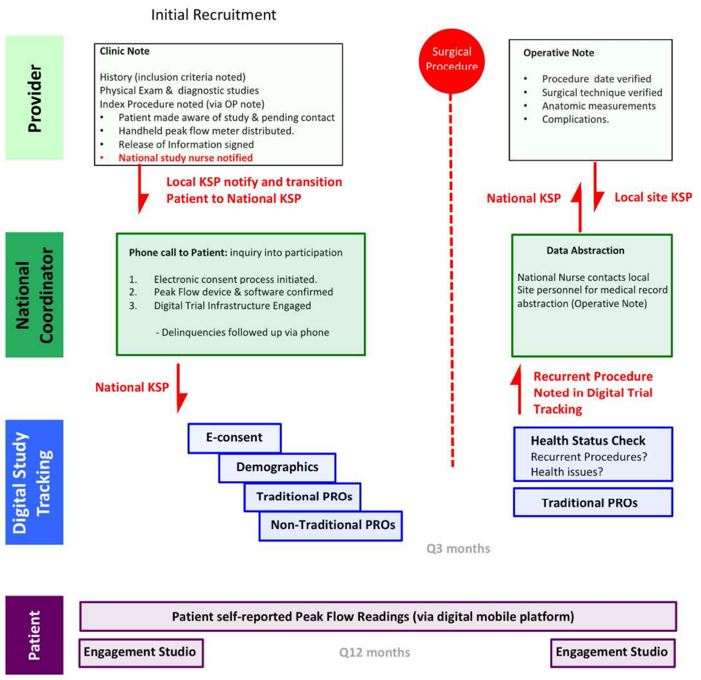

Figure 5. Schematic overview of NoAAC PR02 trial workflow. Data collection for the proposed clinical cohort study will include the case report forms (CRFs), implemented in an electronic data capture (EDC) system.

254x190mm (300 x 300 DPI)

w only

A. B. C.

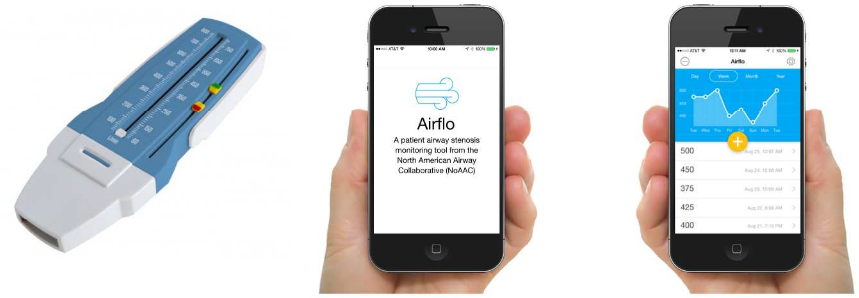

Figure 6. Handheld peak-flow meter (A.), along with mobile device software (B.), for tracking patient-generated health data (C.).

254x190mm (300 x 300 DPI)

w only

Appendix 1.

Data Category	Information
Trial Identifying Number	http://www.clinicalstrials.gov identifier NCT02481817.
Date of Registration	June 25, 2015
Secondary Identifying Numbers	NoAAC PR02
Source of Monetary Support	Patient Centered Outcomes Research Institute (PCORI), Grant ID: 1409-22214
Primary Sponsor	Patient Centered Outcomes Research Institute (PCORI)
Secondary Sponsor	North American Airway Collaborative (NoAAC)
Contact for Public Queries	NoAAC National Trial Coordiator. email: coordinator@noaac.net
Contact for Scientific Queries	NoAAC Director Dr. Alexander Gelbard. email: director@noaac.net
Public Title	Treatment Alternatives in Adult Rare Disease; Assessment of Options in Idiopathic Subglottic Stenosis
Scientific Title	Treatment Alternatives in Adult Rare Disease; Assessment of Options in Idiopathic Subglottic Stenosis North American Airway Collaborative PR-02 Study (NoAAC PR-02 Study)
Countries of Recruitment	International
Health Condition Studied	Idiopatic Subglottic Stenosis (iSGS)
Intervention(s)	Comparison of 3 main surigcal techniques to improve breathing.<br>1. Endoscopic dilation, 2. Endoscopic resection, and 3. Open surgery (tracheal/cricotracheal resection)
Key Inclusion and Exclusion Criteria	Ages eligible for study: ≥18years; Sexes eligible for study:both; Accepts healthy volunteers: no
	Inclusion criteria: Adult patients (≥ 18 years), obstructive airway lesion must involve the subglottis.
	Exclusion criteria: Patients without capacity to consent for themselves. Patients with history endotracheal intubation or tracheotomy within 2 years of first symptoms. Patients with a history of significant laryngotracheal trauma, neck irradiation, caustic or thermal injury to the laryngotracheal complex, or major anterior neck surgery. Patients with a clinical diagnosis of vasculitis or collagen vascular disease, patients with a positive antinuclear cytoplasmic antibody (ANCA) titer.
Study Type	Observational
	Allocation: non-randomized; Intervention model: Pragmatic
	Purpose: Treatment Efficacy
Date of First Enrollment	July 1, 2015
Sample Size	Proposed sample size of 300 (endo dilation ≈ 180, endo resection ≈ 60, open surgery ≈ 60)
Primary Outcome(s)	Treatment effectiveness defined as time to recurrent operative procedure.
Key Secondary Outcomes	Secondary endpoints relate to treatment side effects and include patient reported outcome measures in voice, swallowing, breathing, and global quality of life, as well as patient generated health data.
Ethics Review	This protocol was approved by the IRB Committee of the Vanderbilt University July 2015

NoAAC PR02 - ECONSENT

Study Title: Treatment Alternatives in Adult Rare Disease; Assessment of Options in Idiopathic Subglottic Stenosis (NoAAC PR-02 Study) Version Date 09-15-17

Name of Participant:

____
(First Last)

Age of Participant:

____

1.) What is the purpose of this study?

You are being asked to take part in this research study because you have been diagnosed with Idiopathic Subglottic Stenosis (iSGS). iSGS is a narrowing of your trachea which is the breathing tube from your mouth to your lungs. Your doctor will decide the best course of treatment for you, and we would like to gather information from your chart while you are being treated for this disease. We hope to learn more about common treatments for iSGS, how well they work, and how this disease impacts the quality of your life.

We would also like to collect blood and tissue samples from study patients to help us better understand the causes of iSGS. Our goal is to provide ways to prevent and better manage this disease.

We plan to enroll about 800 people in this study at Vanderbilt. There will be about a total of 1100 people in this study at about 45 sites.

2.) What will happen and how long will you be in the study?

All of the visits and procedures that you have done to treat your disease are part of your regular medical care. They will take place if you are in this study or not. If you agree to be in this study, we will:

** review your medical record to get data about your medical history and health. We will look at information about your diagnosis, tests, and treatments done for it. We will also ask you some questions to try to understand why iSGS occurs.

** ask you to use a handheld asthma monitoring device at home to record your peak flow measurements each day. We will give you one to use during the study.

** ask you to complete eight surveys at several times during the study. If you agree, you will complete these when you first enter the study, after a procedure, at routine follow-up visits (about every 3 months), and if your disease recurs. These will be done for research only and may take less than one hour of your time. We will pay you for each set of surveys that you complete. The surveys will ask questions about your voice, eating and swallowing, overall health status, breathing, etc. We will talk to you about whether you want to complete these in person, by mail, email, or electronically into the research database.

** contact you to discuss your health status and let you know about any other research studies you might be eligible for.

** ask you to take part in 3 focus group interviews with other study participants. These group interviews will be done one time each year and will take about one hour. A focus group is a small group discussion led by a group leader. The leader will ask questions about what it is like to have iSGS and how it affects your life and give the group time to answer them. We encourage everyone to take part in the discussion. The interview will be tape recorded. We will transfer the audio to written notes and study these notes. We will keep the tapes notes in protected computers at Vanderbilt and only the study doctor and his research team will have access to these files. We will talk to you about this more as we get closer to scheduling these interviews.

** ask if we can collect and run tests on some of the tissue from your airway. This would only be done when you are having a biopsy or surgical procedure for your disease. At that time, we would get a small piece of excess tissue study it in the lab.

** ask if we can collect and run tests on your blood. If you agree, we will take about 2.5 tablespoons of blood from a vein in your arm using a needle, at a time when you are having blood drawn for other routine reasons. A research lab will process the blood.

We will record all of this information in a protected database at Vanderbilt, and only the study doctor and his research staff will have access to this database. You will be in the study for about one year.

For peer review only

Genetic/Specimen information

One purpose of this study is to look at genes (DNA) and how they affect health and disease. Genes are the instruction manual for your body. The genes you get from your parents decide what you look like and how your body behaves. They can also tell us a persons risk for certain diseases and how they will respond to treatment.

You are being asked to give blood and tissue samples for genetic research. What we learn about you from these samples will not be put in your health record. Your test results will not be shared with you or your doctor. No one else (like a relative, boss, or insurance company) will be given your test results. Health insurance companies and group health plans may not request your genetic information that comes from this research. Collecting these samples will take about less than 5 minutes of your time.

One risk of giving samples for this research may be the release of your name that could link you to the stored samples and/or the results of the tests run on your samples. This may cause problems with insurance or getting a job.

Health insurance companies and group health plans may not use your genetic information when making decisions regarding your eligibility or premiums. Employers with 15 or more employees may not use your genetic information that comes from this research when making a decision to hire, promote, or fire you or when setting the terms of your employment.

To prevent this, these samples will be given a code. Only the study staff will know the code. The name that belongs to the code will be kept in a locked file or in a computer with a password. Only Dr. Gelbard and his research team will have access to your name.

Your samples will be used to make DNA that will be kept for an unknown length of time (maybe years) for future research. The samples will be destroyed when they are no longer needed.

Your samples may be used to make new products or tests. These may have value and may be developed and owned by the study staff, Vanderbilt University, and/or others. If this happens, there are no plans to provide money to you.

Your samples and information about you may be made available to others to use for research. To protect your privacy, we will not release your name. You will not receive any benefit as a result of the tests done on your samples. These tests may help us learn more about the causes, risks, treatments, or how to prevent this and other health problems. Giving samples for research is your free choice and you may be in the study even if you do not want your samples used or stored for gene research. Giving samples for research is your free choice and you may be in the study even if you do not want your samples used or stored for future research.

At any time, you may ask to have your sample destroyed. You should contact Dr. Gelbard at pager 615-835-9624 to have your sampled destroyed and no longer used for research. We will not be able to destroy research data that has already been gathered using your samples. Also, if your identity was removed from the samples, we will not be able to locate and destroy them.

There will be no costs to you for any of the tests done on your samples. You will not be paid for the use of your samples.

Please check Yes or No to the questions below:

My blood/tissue sample may be used for gene research.	◯ Yes<br>◯ No
My blood/tissue sample may be stored/shared for future gene research in laryngotracheal stenosis disorders.	◯ Yes<br>◯ No
My blood/tissue sample may be stored/shared for future gene research in other health problems (such as cancer, heart disease, etc.)	◯ Yes<br>◯ No

For peer review only

3.) Costs to you if you take part in this study:

There is no cost to you for taking part in this study.

4.) Side effects and risks that you can expect if you take part in this study:

One possible risk to you is the release of information from health records. The chance that this data will be given to someone else by mistake is very small. Ask your doctor for more information about the risks and side effects of procedures that are part of your routine medical care.

Blood samples: You may feel bothered or pained from the needle stick. You may have a bruise or the site may get infected. It is rare, but some people faint.

Additional Information:

5.) Risks that are not known: While we do not know of any risks involved in this study, there may be unknown risks with being in any research study. You will be told of any important findings we learn about related to this study if we think it may change your mind about being in the study.

6.) Payment in case you are injured because of this research study:
If you are injured because you are in this study, you can get reasonable, immediate, and necessary medical care for your injury at Vanderbilt without charge to you. There are no plans for Vanderbilt to pay for the costs of care beyond your injury, or to give you money for such injury.

7.) Good effects that might result from this study:a) The benefits to science and humankind that might result from this study. We hope that we will learn more about the causes for iSGS and better ways to treat people in the future. b) The benefits you might get from being in this study. There is no direct benefit to you from being in this study.

8.) Other treatments you could get if you decide not to be in this study:
This is not a treatment study. If you decide not to be part of the study, it will not affect your regular medical care.

9.) Payments for your time spent taking part in this study or expenses: You will be paid $40 for each set of surveys you complete for being in this study.

10.) Reasons why the study doctor may take you out of this study: There is no reason that the study doctor would take you out of this study.

11.) What will happen if you decide to stop being in this study? Taking part in this study is voluntary. If you decide to stop being part of the study, you should tell the study doctor.

12.) Who to call for any questions or in case you are injured: If you should have any questions about this research study or if you feel you have been hurt by being a part of this study, please feel free to contact Dr. Gelbard at 615-835-9624. If you cannot reach the research staff, please page the study doctor at by calling 615-322-5000 and ask the operator to page him.

For additional information about giving consent or your rights as a person in this study, to discuss problems, concerns, and questions, or to offer input, please feel free to call the Vanderbilt University Institutional Review Board Office at (615) 322-2918 or toll free at (866) 224-8273.

13.) Clinical Trials Registry: A description of this clinical trial will be available on www.clinicaltrials.gov, as required by U.S. Law. This Web site will not include information that can identify you. At most, the Web site will include a summary of the results. You can search this Web site at any time.

For peer review only - http://bmjopen.bmj.com/site/about/guidelines.xhtml

Information on Confidentiality, Authorization to Use/Disclose Protected Health Information, and Agreement to Participate in Study.

14.) Confidentiality: You will be assigned a study ID number that will be attached to your records to keep the information confidential. Your records will be kept in a password-protected database. Only the study doctor and his research team will have access to this database.

Vanderbilt may share your information, without identifiers, to others or use it for other research projects not listed in this form. Vanderbilt, Dr. Gelbard and his staff will comply with any and all laws regarding the privacy of such information. There are no plans to pay you for the use or transfer of this de-identified information.

Proceed to review of confidentiality, use/disclosure of protected health information authorization and agreement to participate in study?

◯ Yes
◯ No

15.) Authorization to Use/Disclose Protected Health Information

All efforts, within reason, will be made to keep your protected health information (PHI) private. PHI is your health information that is, or has been gathered or kept by Vanderbilt as a result of your healthcare. This includes data gathered for research studies that can be traced back to you. Using or sharing ("disclosure") such data must follow federal privacy rules. By signing the consent for this study, you are agreeing ("authorization") to the uses and likely sharing of your PHI. If you decide to be in this research study, you are also agreeing to let the study team use and share your PHI as described below.

As part of the study, Dr. Gelbard and his study team may share the results of your study and/or non-study linked diagnostic tests, physical exams, operative reports, etc., as well as parts of your medical record, to the groups named below. These groups may include people from the Federal Government Office for Human Research Protections and the Vanderbilt University Institutional Review Board. Federal privacy rules may not apply to these groups; they have their own rules and codes to assure that all efforts, within reason, will be made to keep your PHI private.

The study results will be kept in your research record for at least six years after the study is finished. At that time, the research data that has not been put in your medical record will be kept for an unknown length of time. Any research data that has been put into your medical record will be kept for an unknown length of time.

Unless told otherwise, your consent to use or share your PHI does not expire. If you change your mind, we ask that you contact Dr. Gelbard in writing and let him know that you withdraw your consent. His mailing address is:

Dr. Alexander Gelbard
Vanderbilt Voice Center
Vanderbilt University Medical Center
1215 21st Ave. South
7209 Medical Center East, South Tower
Nashville, TN 37232-8605

At that time, we will stop getting any more data about you. But, the health data we stored before you withdrew your consent may still be used for reporting and research quality.

If you decide not to take part in this research study, it will not affect your treatment, payment or enrollment in any health plans or affect your ability to get benefits. You will get a copy of this form after it is signed.

Statement by person agreeing to be in this study:

◯ I have read this consent form and the research study has been explained to me verbally. All my questions have been answered, and I freely and voluntarily choose to take part in this study.

Patient/Volunteer Name:

____
(First Last)

For peer review only

Please provide your signature by choosing one of the two methods below - typing your name in the box, or providing a 'wet' signature.

Participant/Volunteer signature - typed name in box

____
(please type your full name in this box confirming your signature)

Participant/Volunteer 'wet' signature

____
(please provide your signature using your cursor or finger)

Date:

____

Consent obtained by (please enter full name and title):

____
(Completed by study personnel at time of consent.)

Date:

____

For peer review only

Treatment Alternatives in Adult Rare Disease; Assessment of Options in Idiopathic Subglottic Stenosis. North American Airway Collaborative PR-02 Study, A Prospective Pragmatic Trial.

Local Site Investigator Surgical Protocol

NoAAC
the north american airway collaborative

Provider
Name
Academic Rank (*circle one*):
- Instructor
- Assistant Professor
- Associate Professor
- Professor

Institution
Fellowship training (*circle one*):
- Pediatric Oto,
- Laryngology
- Head & Neck
- None

Years out of training (*circle one*):
- 1-5
- 5-10
- >10

Support Staff
Administrative Assistant:

Research Coordinator:

iSGS: Provider Specific Management
Initial Work-up (*circle all that apply*):
- Labs
- PFTs
- GI (pH, impedance and/or endoscopy)
- CT scan

Criteria for intervention (*circle all that apply*):
- Patient Symptoms,
- Physical Exam
- Objective measures
  - (ie. PFTs or Peak Flow meter)

Open Technique:
Number of Open Cases/year ____
Criteria for recommendation for open surgery
Describe:

Surgical Technique (*circle one*):
- Cricotracheal Resection
- Laryngotracheoplasty
- Other

Endoscopic Technique:
Anesthesia (*circle one*):
- Spontaneous Ventilation
- Jet Ventilation
- LMA
- Ventilating Bronchoscope

Scar management (*circle one*):
- CO2 laser incisions
- cold knife
- none
- CO2 laser resection of scar
- other (explain)

Dilation Technique (*circle one*):
- Balloon
- Bougie
- Pediatric Bronchoscope
- No dilation

Adjuvant Therapy (*circle one*):
- PPI
- Inhaled Corticosteroid
- Antibiotics (ie Bactrim or Azithro)
- Other - describe
- Transcutaneous Steroids
- None

For peer review only

Reporting checklist for protocol of a clinical trial.

Based on the SPIRIT guidelines.

Instructions to authors

Complete this checklist by entering the page numbers from your manuscript where readers will find each of the items listed below.

If your work does not currently contain an item consider adding it. If you are certain that an item does not apply to your work, write 'n/a'. If you like, you can provide a short reason.

Upload this checklist as an extra file when you submit to a journal.

In your methods section, say that you used the SPIRIT reporting guidelines, and cite them as:

Chan A-W, Tetzlaff JM, Altman DG, Laupacis A, Gøtzsche PC, Krleža-Jerić K, Hróbjartsson A, Mann H, Dickersin K, Berlin J, Doré C, Parulekar W, Summerskill W, Groves T, Schulz K, Sox H, Rockhold FW, Rennie D, Moher D. SPIRIT 2013 Statement: Defining standard protocol items for clinical trials. Ann Intern Med. 2013;158(3):200-207

		Reporting Item	Page Number
Title	#1	Descriptive title identifying the study design, population, interventions, and, if applicable, trial acronym	3
Trial registration	#2a	Trial identifier and registry name. If not yet registered, name of intended registry	3
Trial registration: data set	#2b	All items from the World Health Organization Trial Registration Data Set	3
Protocol version	#3	Date and version identifier	3
Funding	#4	Sources and types of financial, material, and other support	3
Roles and responsibilities: contributorship	#5a	Names, affiliations, and roles of protocol contributors	3

For peer review only

Roles and responsibilities: sponsor contact information	#5b	Name and contact information for the trial sponsor	3
Roles and responsibilities: sponsor and funder	#5c	Role of study sponsor and funders, if any, in study design; collection, management, analysis, and interpretation of data; writing of the report; and the decision to submit the report for publication, including whether they will have ultimate authority over any of these activities	3
Roles and responsibilities: committees	#5d	Composition, roles, and responsibilities of the coordinating centre, steering committee, endpoint adjudication committee, data management team, and other individuals or groups overseeing the trial, if applicable (see Item 21a for data monitoring committee)	3
Background and rationale	#6a	Description of research question and justification for undertaking the trial, including summary of relevant studies (published and unpublished) examining benefits and harms for each intervention	4
Background and rationale: choice of comparators	#6b	Explanation for choice of comparators	4
Objectives	#7	Specific objectives or hypotheses	5
Trial design	#8	Description of trial design including type of trial (eg, parallel group, crossover, factorial, single group), allocation ratio, and framework (eg, superiority, equivalence, non-inferiority, exploratory)	5
Study setting	#9	Description of study settings (eg, community clinic, academic hospital) and list of countries where data will be collected. Reference to where list of study sites can be obtained	5
Eligibility criteria	#10	Inclusion and exclusion criteria for participants. If applicable, eligibility criteria for study centres and	5

For peer review only

		individuals who will perform the interventions (eg, surgeons, psychotherapists)	
Interventions: description	#11a	Interventions for each group with sufficient detail to allow replication, including how and when they will be administered	5
Interventions: modifications	#11b	Criteria for discontinuing or modifying allocated interventions for a given trial participant (eg, drug dose change in response to harms, participant request, or improving / worsening disease)	5
Interventions: adherance	#11c	Strategies to improve adherence to intervention protocols, and any procedures for monitoring adherence (eg, drug tablet return; laboratory tests)	6
Interventions: concomitant care	#11d	Relevant concomitant care and interventions that are permitted or prohibited during the trial	6
Outcomes	#12	Primary, secondary, and other outcomes, including the specific measurement variable (eg, systolic blood pressure), analysis metric (eg, change from baseline, final value, time to event), method of aggregation (eg, median, proportion), and time point for each outcome. Explanation of the clinical relevance of chosen efficacy and harm outcomes is strongly recommended	6
Participant timeline	#13	Time schedule of enrolment, interventions (including any run-ins and washouts), assessments, and visits for participants. A schematic diagram is highly recommended (see Figure)	6
Sample size	#14	Estimated number of participants needed to achieve study objectives and how it was determined, including clinical and statistical assumptions supporting any sample size calculations	6
Recruitment	#15	Strategies for achieving adequate participant enrolment to reach target sample size	6
Allocation: sequence generation	#16a	Method of generating the allocation sequence (eg, computer-generated random numbers), and list of any	n/a

For peer review only

		factors for stratification. To reduce predictability of a random sequence, details of any planned restriction (eg, blocking) should be provided in a separate document that is unavailable to those who enrol participants or assign interventions	
Allocation concealment mechanism	#16b	Mechanism of implementing the allocation sequence (eg, central telephone; sequentially numbered, opaque, sealed envelopes), describing any steps to conceal the sequence until interventions are assigned	n/a
Allocation: implementation	#16c	Who will generate the allocation sequence, who will enrol participants, and who will assign participants to interventions	n/a
Blinding (masking)	#17a	Who will be blinded after assignment to interventions (eg, trial participants, care providers, outcome assessors, data analysts), and how	n/a
Blinding (masking): emergency unblinding	#17b	If blinded, circumstances under which unblinding is permissible, and procedure for revealing a participant's allocated intervention during the trial	n/a
Data collection plan	#18a	Plans for assessment and collection of outcome, baseline, and other trial data, including any related processes to promote data quality (eg, duplicate measurements, training of assessors) and a description of study instruments (eg, questionnaires, laboratory tests) along with their reliability and validity, if known. Reference to where data collection forms can be found, if not in the protocol	6
Data collection plan: retention	#18b	Plans to promote participant retention and complete follow-up, including list of any outcome data to be collected for participants who discontinue or deviate from intervention protocols	6
Data management	#19	Plans for data entry, coding, security, and storage, including any related processes to promote data quality (eg, double data entry; range checks for data values).	7

For peer review only

		Reference to where details of data management procedures can be found, if not in the protocol	
Statistics: outcomes	#20a	Statistical methods for analysing primary and secondary outcomes. Reference to where other details of the statistical analysis plan can be found, if not in the protocol	9
Statistics: additional analyses	#20b	Methods for any additional analyses (eg, subgroup and adjusted analyses)	9
Statistics: analysis population and missing data	#20c	Definition of analysis population relating to protocol non-adherence (eg, as randomised analysis), and any statistical methods to handle missing data (eg, multiple imputation)	9
Data monitoring: formal committee	#21a	Composition of data monitoring committee (DMC); summary of its role and reporting structure; statement of whether it is independent from the sponsor and competing interests; and reference to where further details about its charter can be found, if not in the protocol. Alternatively, an explanation of why a DMC is not needed	11
Data monitoring: interim analysis	#21b	Description of any interim analyses and stopping guidelines, including who will have access to these interim results and make the final decision to terminate the trial	11
Harms	#22	Plans for collecting, assessing, reporting, and managing solicited and spontaneously reported adverse events and other unintended effects of trial interventions or trial conduct	11
Auditing	#23	Frequency and procedures for auditing trial conduct, if any, and whether the process will be independent from investigators and the sponsor	11
Research ethics approval	#24	Plans for seeking research ethics committee / institutional review board (REC / IRB) approval	12

For peer review only

Protocol amendments	#25	Plans for communicating important protocol modifications (eg, changes to eligibility criteria, outcomes, analyses) to relevant parties (eg, investigators, REC / IRBs, trial participants, trial registries, journals, regulators)	12
Consent or assent	#26a	Who will obtain informed consent or assent from potential trial participants or authorised surrogates, and how (see Item 32)	12
Consent or assent: ancillary studies	#26b	Additional consent provisions for collection and use of participant data and biological specimens in ancillary studies, if applicable	12
Confidentiality	#27	How personal information about potential and enrolled participants will be collected, shared, and maintained in order to protect confidentiality before, during, and after the trial	12
Declaration of interests	#28	Financial and other competing interests for principal investigators for the overall trial and each study site	12
Data access	#29	Statement of who will have access to the final trial dataset, and disclosure of contractual agreements that limit such access for investigators	12
Ancillary and post trial care	#30	Provisions, if any, for ancillary and post-trial care, and for compensation to those who suffer harm from trial participation	12
Dissemination policy: trial results	#31a	Plans for investigators and sponsor to communicate trial results to participants, healthcare professionals, the public, and other relevant groups (eg, via publication, reporting in results databases, or other data sharing arrangements), including any publication restrictions	12
Dissemination policy: authorship	#31b	Authorship eligibility guidelines and any intended use of professional writers	12
Dissemination policy: reproducible research	#31c	Plans, if any, for granting public access to the full protocol, participant-level dataset, and statistical code	12

For peer review only

Informed consent materials	#32	Model consent form and other related documentation given to participants and authorised surrogates	Appendix 2
Biological specimens	#33	Plans for collection, laboratory evaluation, and storage of biological specimens for genetic or molecular analysis in the current trial and for future use in ancillary studies, if applicable	Appendix 2

The SPIRIT checklist is distributed under the terms of the Creative Commons Attribution License CC-BY-ND 3.0. This checklist was completed on 07. February 2018 using http://www.goodreports.org/, a tool made by the EQUATOR Network in collaboration with Penelope.ai

For peer review only
