# Supplementary material for: Treatment options in idiopathic subglottic stenosis: protocol for a prospective international multicentre pragmatic trial
Source: BMJ Open. 2018 Apr 10;8(4):e022243. doi: 10.1136/bmjopen-2018-022243 (PMC5898326; doi:10.1136/bmjopen-2018-022243)
Supplement: Reviewer comments [file bmjopen-2018-022243.reviewer_comments.pdf]

PEER REVIEW HISTORY

BMJ Open publishes all reviews undertaken for accepted manuscripts. Reviewers are asked to complete a checklist review form (**http://bmjopen.bmj.com/site/about/resources/checklist.pdf**) and are provided with free text boxes to elaborate on their assessment. These free text comments are reproduced below.

ARTICLE DETAILS

**TITLE (PROVISIONAL)**	Treatment Options in Idiopathic Subglottic Stenosis: Protocol for a Prospective International Multicenter Pragmatic Trial
**AUTHORS**	Gelbard, Alexander; Shyr, Yu; Berry, Lynne; Hillel, Alexander; Ekbom, Dale; Edell, Eric; Kasperbauer, Jan; Lott, David; Donovan, Donald; Garrett, C. Gaelyn; Sandhu, Guri; Daniero, James; Netterville, James; Schindler, Josh; Smith, Marshall; Bryson, Paul; Lorenz, Robert; Francis, David

This article was not externally reviewed at BMJ Open. Protocols that have been independently assessed prior to submission to BMJ Open are usually fast-tracked to publication on the grounds that further substantial changes will not be possible. This independent assessment will usually be external, independent review for both a substantial grant award from a non-commercial or government funder and ethics approval.
